# Single-cell CyTOF profiling reveals alterations in B, T and macrophage subsets during murine hepatic aging

**DOI:** 10.3389/fimmu.2026.1787641

**Published:** 2026-04-28

**Authors:** Zheng Ding, Bing Fang, Jing Peng, Siyu Wang, Xiaomin Tian, Guixi Chen, Yuqiu Wei, Yuebin Gao, Yixuan Li, Jiazeng Sun

**Affiliations:** 1 Key Laboratory of Precision Nutrition and Food Quality, Department of Nutrition and Health, China Agricultural University, Beijing, China; 2 College of Biological Sciences, China Agricultural University, Beijing, China; 3 Beijing Life Science Academy, Beijing, China; 4 Henan University of Technology, Zhengzhou, China

**Keywords:** B cell depletion, Cytometry by Time-of-Flight (CyTOF), hepatic immune landscape, liver aging, naive B cells

## Abstract

**Introduction:**

Population aging is a global demographic shift closely associated with immune dysregulation, which significantly increases the risk of chronic liver diseases. As a crucial immune and metabolic organ, the liver relies on a complex immune network consisting of innate and adaptive cell subsets to maintain homeostasis and defend against invading pathogens. However, the remodeling mechanism of the hepatic immune landscape with aging remains incompletely understood.

**Methods:**

We investigated the changes in hepatic immune cell subsets and related molecular profiles during aging, focusing on the identification and characterization of key immune cell populations and their associated gene expression patterns in aged livers.

**Results:**

We identified Igd+ B cells in the hepatic immune compartment, which showed a significant reduction in aged livers. This reduction was accompanied by the enrichment of B cell signal transduction pathways and downregulation of genes related to cell migration and receptor binding. Meanwhile, aged livers exhibited selective expansion of multiple CD4+ T cell subsets (Th1, Th2, Th17, Treg) and an increase in resident-derived pro-inflammatory M1 macrophages, whereas CD8+ T cells, double-negative T cells, and most innate lymphoid cell subsets remained stable. Our study delineated age-associated alterations in hepatic B, T, and macrophage subsets as a characteristic feature of murine hepatic immune aging.

**Discussion:**

These findings clarify the characteristics of the hepatic immune landscape during aging, providing a valuable reference for future studies on liver aging and the development of strategies to address age-related liver immune dysregulation.

## 1 Introduction

The worldwide demographic panorama is experiencing an unparalleled transformation toward population aging, with the share of people aged 60 years and over forecasted to increase twofold by 2050 (1, 2). With aging, the incidence of various liver diseases increases, and aging also exacerbates their progression (3–5). In human studies, the aged liver is reduced in size due to decreased blood flow and bile flow, and exhibits diminished regenerative capacity (6, 7). The human liver undergoes age-related changes in the global transcriptome and microRNA profiles, epigenetic alterations tend to stabilize with advanced age, and telomere length is shortened in an age-dependent manner (8, 9). Despite substantial advances in geriatric medicine, the molecular mechanisms underlying the interplay between aging, immune dysregulation, and liver chronic disease progression remain incompletely elucidated (10–12).

The liver serves as a critical frontline immune organ, strategically positioned to sense and eliminate gut-derived pathogens (12, 13). Notably, in contrast to lymphoid organs that favor adaptive immunity, hepatic immune responses are dominated by innate immune cells (14–16). The liver encompasses a heterogeneous array of innate immune cells—including Kupffer cells (tissue-resident macrophages), dendritic cells, and granulocytes—resident within the parenchyma and sinusoids, while a diverse repertoire of lymphocytes (T cells, B cells and NK cells) and unconventional subsets also populates this organ (17–19). Research on age-related changes in the hepatic immune system remains limited and incomplete. To date, only one human study has mentioned hepatic immune cells in aging. However, the study focused on global changes in the liver and did not address alterations in immune cells (20). In mice, previous studies have focused primarily on macrophages (Kupffer cells). Hepatic macrophages in aged mice exhibit an enhanced pro-inflammatory phenotype alongside subcellular changes like more electron-dense deposits (21–24). They secrete more inflammatory cytokines and chemokines, and reduced cytoskeleton elements and contribute to the development and progression of chronic liver disease (25–27). Neutrophils are recruited to the liver of aged mice, thereby inducing hepatic inflammatory infiltration and triggering liver injury (27–29). Liver T cells exhibit senescent and exhausted phenotypes and are associated with metabolic diseases and PD-1+CD4+ population was present in old mice liver, spleen, peritoneum and lungs (30, 31), However, studies on other immune cells and their subtypes in the liver of aged mice remain extremely rare. A lack of comprehensive insights into how the hepatic immune system remodels during aging restricts in-depth studies.

To address this critical knowledge gap, our research employed Cytometry by Time-of-Flight (CyTOF) as the core technical platform (32). Distinguished by its ability to simultaneously quantify dozens of protein markers at single-cell resolution, CyTOF offers an unparalleled advantage in directly capturing cellular phenotypic characteristics and functional states from the protein level (33).

Our data identified three key age-related alterations in hepatic immune subsets: naive B cells underwent marked reduction; multiple CD4^+^ T cell subsets exhibited selective expansion; and resident-derived pro-inflammatory M1 macrophages were significantly elevated. These changes collectively impair hepatic immune surveillance and metabolic homeostasis, offering novel insights into the mechanisms driving age-related liver immune dysfunction.

## 2 Materials and methods

### 2.1 Animals

A total of 12 C57BL/6 mice (Vital River) were included in the study, with 6 young (3 months old) and 6 aged (24 months old) counterparts; males and females were equally distributed in each group. The mice were housed under specific pathogen-free (SPF) conditions at the Animal Facility of China Agricultural University. Parameters included a constant temperature of 22 ± 2 °C, 50 ± 5% relative humidity, and a 12-hour light/dark cycle (light phase: 07:00–19:00). Sterilized diet and filtered water were supplied ad libitum throughout the study. All procedures involving laboratory animals were approved by and performed in compliance with the institutional animal care protocols. Consistent with the 3Rs ethical framework for animal experimentation, measures were taken to optimize experimental design, thereby minimizing animal discomfort and reducing the number of mice required for statistical validity.

### 2.2 Liver collection

Mice were anesthetized with 3–4% isoflurane (inhaled, delivered in 100% oxygen at a flow rate of 2–3 L/min) for induction, followed by maintenance with 1–2% isoflurane (inhaled, delivered in 100% oxygen at a flow rate of 1–2 L/min) to ensure a surgical plane of anesthesia (assessed by the absence of pedal withdrawal reflex). Deep anesthesia was achieved via intraperitoneal injection of sterile 2.5% Avertin solution (Cat. IR9184, Beijing Solarbio) at a dose of 2.5 mg/10 g body weight (0.1 mL per 10 g body weight). The abdominal cavity was immediately opened under deep anesthesia with sterile surgical instruments under a laminar flow hood to maintain sterility. Livers from mice were collected starting at 4:00 PM, and all liver samples were harvested by 5:00 PM on the same day. The liver was carefully dissected free from adjacent tissues using sterile surgical instruments. The gallbladder was gently removed to avoid bile contamination. After completion of the experimental procedures, mice were euthanized via abdominal aortic exsanguination while still under deep anesthesia, which was performed to minimize animal distress and suffering in accordance with institutional ethical guidelines.

### 2.3 Preparation of cell suspensions

Mouse liver tissues were carefully transferred to a sterile Petri dish containing pre-chilled phosphate-buffered saline (PBS) to prevent tissue desiccation. Using sterile surgical scissors, the liver tissues were minced into uniform small pieces (approximately 1–2 mm^3^) to maximize the contact area between the tissue and the digestive solution. The minced liver fragments were then transferred to a 50 mL conical centrifuge tube, and an appropriate volume of Liver Dissociation Kit (Miltenyi) working solution was added in accordance with the manufacturer protocol. The tube was tightly capped and incubated in water bath at 37 °C with constant gentle agitation (120–150 rpm) for 1 hour to ensure thorough enzymatic digestion of the hepatic extracellular matrix and tissue dissociation. Upon completion of digestion, the resulting tissue homogenate (dissociated tissue soup) was sequentially filtered through a 70 μm sterile cell strainer placed on a new centrifuge tube; the strainer was gently pressed with a sterile plunger to facilitate the passage of single cells and minimize cell loss. The filtered cell suspension was centrifuged at 300 × g for 5 min at 4 °C to pellet cells, with subsequent supernatant discard. The cell pellet was resuspended in pre-cooled ACK lysis buffer (ammonium-chloride-potassium buffer), and the mixture was placed on ice for incubation at 5 min to selectively lyse contaminating red blood cells (RBCs) without compromising the viability of hepatic immune cells. The lysis reaction was terminated by adding 3 volumes of pre-cooled PBS, and the suspension was re-centrifuged at 300 × g, 5 min, 4 °C to harvest the purified cell pellet.

### 2.4 Mass cytometry staining and data acquisition

Cells were initially washed to eliminate residual cellular debris and non-specific binding contaminants. The cell pellet was then resuspended in 100 μL of 250 nM cisplatin solution (Fluidigm) and incubated on ice for 5 min, a step that permits the selective labeling of dead cells for exclusion during subsequent data analysis. Following cisplatin incubation, cells were subjected to centrifugation and the supernatant was discarded. The cell pellet was subsequently resuspended in Fc receptor blocking buffer and incubated on ice to abrogate non-specific antibody binding mediated by Fc receptors. Thereafter, cells were stained with a pre-optimized antibody cocktail for 30 min, with gentle vortexing at 10-min intervals to guarantee homogeneous staining. The antibodies utilized in this study is provided in **Supplementary Table 1**.

After surface staining, cells were rinsed twice with ice-cold FACS buffer (BD), with centrifugation performed between each wash to eliminate unbound surface antibodies. The resulting cell pellet was then fixed in 200 μL of Maxpar Fix and Perm Buffer (Fluidigm) containing 250 nM^191/193^ Ir, followed by incubation at 4 °C in the dark; this step preserved cellular integrity while enabling DNA staining for subsequent cell cycle profiling and doublet exclusion.

Post-overnight fixation, cells were washed with ice-cold FACS buffer. The pellet was resuspended in ice-cold permeabilization buffer (eBioscience) and incubated on ice for 20 min to disrupt the cell membrane, thereby facilitating intracellular antibody infiltration. Cells were subsequently stained with an antibody cocktail for 0.5 h on ice in the dark. Finally, stained cells were washed twice with ice-cold FACS buffer, resuspended in deionized water, and spiked with a 20% aliquot of EQ calibration beads (Fluidigm) for instrument normalization. Samples were then acquired on a Helios mass cytometer (Fluidigm) for high-dimensional single-cell immunophenotypic analysis.

### 2.5 CyTOF data analysis

First, leveraging specific mass-tagged barcodes, a doublet-filtering strategy was applied to decode and separate the data of each individual sample from the raw dataset (34). Bead-based normalization was performed to standardize FCS files derived from separate experimental batches (35). Manual gating was subsequently performed on the dataset using FlowJo software to eliminate cellular debris, dead cells, and cell doublets, thus isolating viable, single immune cells. Subsequently, the X-shift clustering algorithm was deployed across the full cell population to categorize cells into discrete phenotypic subsets based on marker expression magnitudes, and each cluster was assigned a cell type identity according to its signature marker expression profile, as depicted in a cluster-versus-marker heatmap (36). Then, the t-distributed stochastic neighbor embedding (t-SNE) dimensionality reduction algorithm was employed to project the dataset into two dimensions, thereby visualizing the spatial distribution of individual clusters, the expression profiles of target markers, and the heterogeneity across experimental groups or sample types.

### 2.6 scRNA-seq data analysis

The single-cell transcriptomic profiles of murine liver tissue were curated from the Single-cell Aging Atlas, a component of the Aging Atlas platform (https://bigd.big.ac.cn/aging/index), hosted by the National Genomics Data Center (NGDC). As an open-access, registration-free resource, the platform enables direct retrieval of scRNA-seq matrices through its interactive module under the path: Home → The Single-cell Aging Atlas → mouse_exercise_tissues. The mice used in this study were C57BL/6J male mice, divided into 2-month-old and 16-month-old groups, with 11 mice in each group. From this dataset, we extracted gene-level expression counts, performed differential expression analysis, and subjected the resulting signature gene set to functional pathway enrichment—thereby interrogating molecular pathways modulated by exercise in the context of hepatic aging.

### 2.7 Statistical analysis

GraphPad Prism software was used for all statistical analyses and figure generation. Quantification data are presented as mean ± SD. Unpaired Student’s t-tests were utilized to compare the difference between young and old mice, and no other statistical methods were employed. Statistical significance was assigned when the *p*-value was less than 0.05, with different significance levels indicated in figures.

## 3 Results

### 3.1 B cell proportion decreased significantly in major population gating of aged mouse livers

To characterize age-related alterations in the hepatic immune landscape, we performed single-cell mass cytometry (CyTOF) profiling of immune cells from livers of young (3 months, n = 6) and old (24 months, n = 6) mice (**Supplementary Figure 1a**). Isolated liver tissues were processed into single-cell suspensions (viability >85%, viable cells >10^6^), which met CyTOF acquisition requirements. Cells were stained with a panel of metal-conjugated antibodies targeting key surface and intracellular markers to achieve comprehensive immunophenotyping. After antibody staining, the single-cell suspensions were loaded into the CyTOF instrument for high-dimensional data acquisition. Raw CyTOF data were processed through a rigorous computational pipeline (**Supplementary Figure 1b**). Dead cells, debris, and non-cellular beads were excluded based on characteristic signals of Cisplatin and Event_length. Residual interfering beads were further removed via a “beads-” gating strategy. Intact cells were defined by positive DNA1 signals to exclude singlets and doublets, thus ensuring the integrity of the target cell population. This stringent gating generated a clean dataset of viable immune cells for subsequent analysis. Following CD45^+^ cell gating, the average yield of immune cells per mouse liver sample exceeded 1×10^5^ cells, which was sufficient to meet technical requirements for downstream immune cell subset analysis.

**Figure 1:**
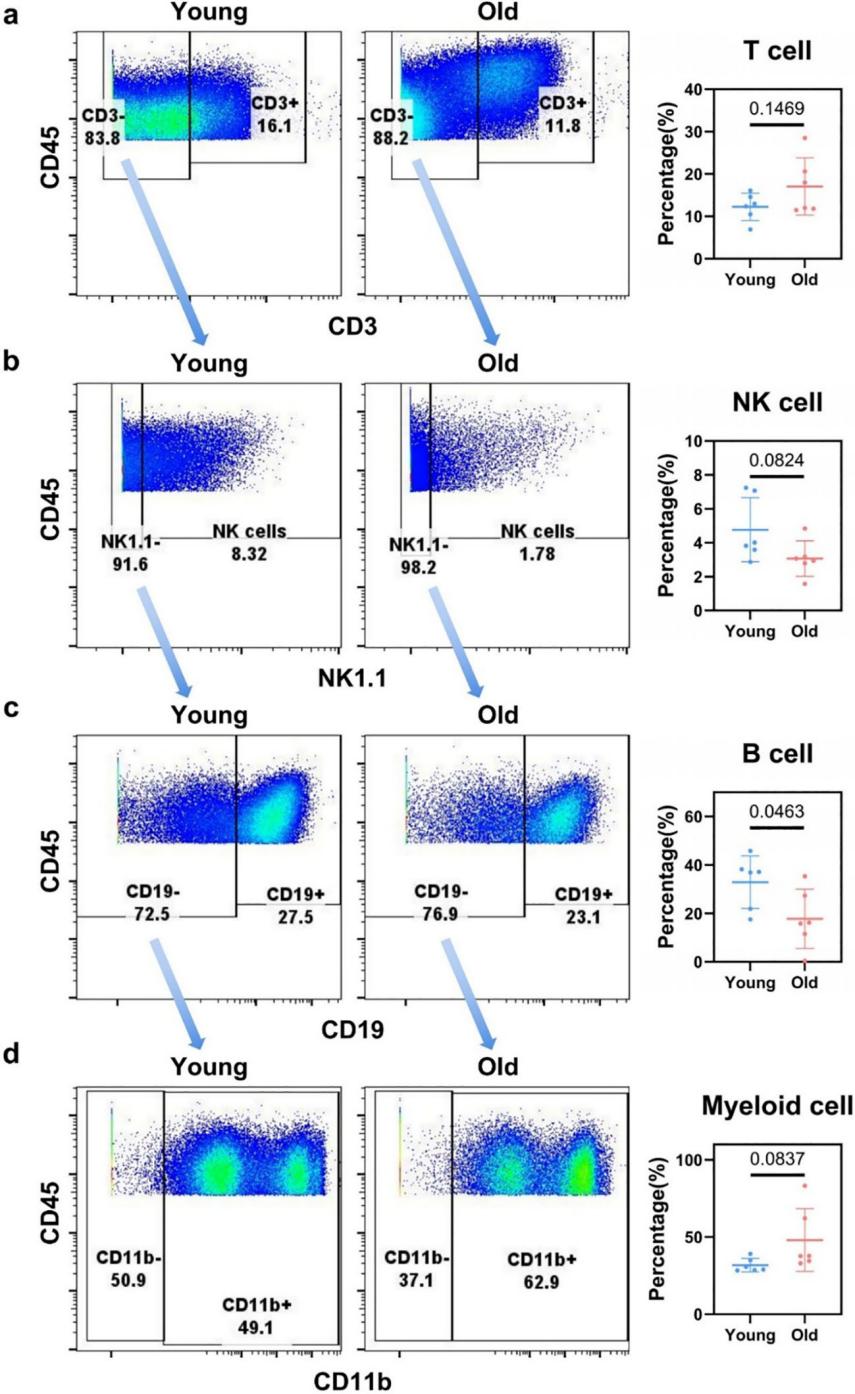
Proportion of major hepatic immune cells in young vs. old mice. **(a)** Flow cytometry analysis for identifying CD3^+^ T cells within the CD45^+^ immune cell population in the livers of young and aged mice, with data on the proportion of CD3^+^ T cells in each group. **(b)** Flow cytometry - based gating to define CD3^–^NK1.1^+^ NK cells among CD45^+^ cells in the livers of young and aged mice, along with the corresponding proportion data of NK cells. **(c)** Flow cytometry gating strategy for isolating CD19^+^ B cells from CD3^–^NK1.1^–^ CD45^+^ cells in the livers of young and aged mice, presenting the proportion of CD19^+^ B cells. **(d)** Flow cytometry gating for identifying CD19^–^CD11b^+^ myeloid cells within CD45^+^ cells in the livers of young and aged mice, accompanied by the proportion data of myeloid cells.

To precisely delineate immune cell subsets, we employed a manual gating strategy to identify diverse populations (**Supplementary Figure 2**) based on the expression patterns of canonical surface markers and functional markers. The detailed gating strategy is provided in the following sections. Through manual gating (**Supplementary Figure 2**), we identified five major categories of immune cells in the liver, namely T cells, NK cells, B cells, myeloid cells, and innate lymphoid cells (ILCs). Following flow cytometry gating of CD45^+^ cells, the target immune cell fraction was collected (**Supplementary Figures 1b**, **S2**). T cells were gated on CD3^+^ expression for identification, and statistical analysis revealed no significant difference in their proportion between young and aged livers (*p* = 0.1469, **Figure 1a**). NK cells were gated as NK1.1^+^CD3^–^ and statistical analysis confirmed that the NK cell proportion between young and aged mice was not significant (*p* = 0.0824, **Figure 1b**). Myeloid cells, identified through CD19^–^/CD11b^+^ gating, also showed lacked significant change (*p* = 0.0837, **Figure 1d**). Notably, in contrast to the non-significant changes observed in the above immune cell subsets, CD19^+^ B cells—identified via gating on the NK1.1^–^ subset—exhibited a striking and statistically significant reduction in aged murine livers (*p* = 0.0463, **Figure 1c**). Specifically, the proportion of B cells plummeted by approximately 50% in aged mice compared with young controls, dropping from 32.90% in the young group to 17.75% in the aged group. Collectively, we report for the first time that the proportion of B cells in the aged liver undergoes a significant reduction at the level of major immune cells.

Statistical significance for **Figure 1** was defined as *p* < 0.05 and *p* < 0.01 representing different significance levels.

### 3.2 The proportions of CD4^+^ T cell subtypes are increased in the aged livers

**Figure 2:**
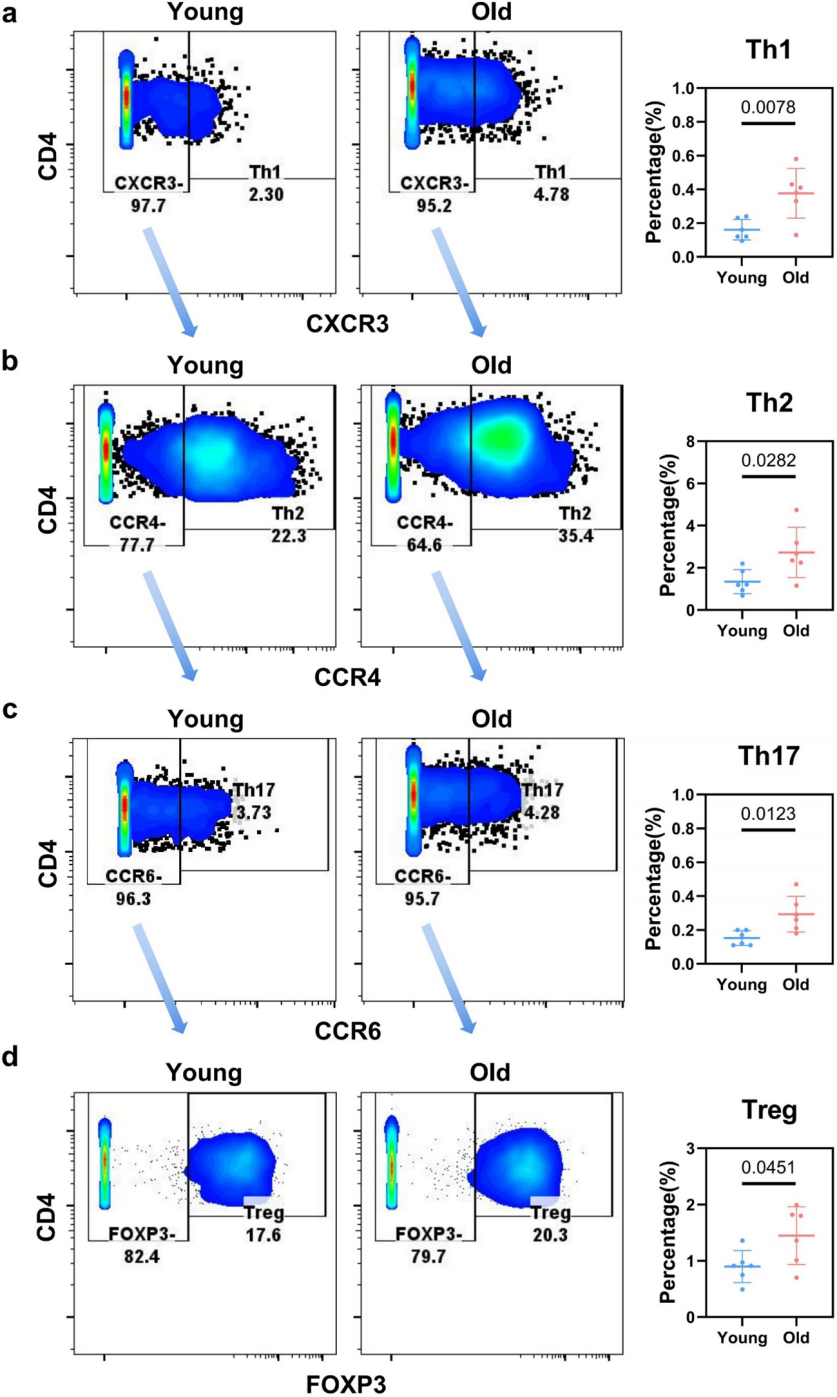
Flow cytometry gating of CD4^+^ cell subtypes in livers of toung vs. old mice. **(a)** Flow cytometry plots (CD4 vs. CXCR3 gating) showing the distribution of CXCR3^–^ and Th1 (CXCR3^+^) subsets, with quantification of Th1 cell percentage. **(b)** Flow cytometry plots (CD4 vs. CCR4 gating) showing the distribution of CCR4^–^ and Th2 (CCR4^+^) subsets, with quantification of Th2 cell percentage. **(c)** Flow cytometry plots (CD4 vs. CCR6 gating) showing the distribution of CCR6^–^ and Th17 (CCR6^+^) subsets, with quantification of Th17 cell percentage. **(d)** Flow cytometry plots (CD4 vs. FOXP3 gating) showing the distribution of FOXP3^–^ and Treg (FOXP3^+^) subsets, with quantification of Treg cell percentage.

Following the gating sequence, we first analyzed NK cells and T cells. CD49b-based gating classified CD3^–^ NK cells into iNK and mNK subsets: iNK proportions were comparable between groups (*p* = 0.2353), while mNK proportions declined significantly (*p* = 0.0080, **Supplementary Figure 3**), potentially reducing anti-tumor and antiviral activity (37). After excluding NK1.1^+^ NKT cells from CD3^+^ subsets by gating, NKT cells exhibited low abundance and no age-related proportional difference (*p* = 0.4018, **Supplementary Figure 4a**). Within the NK1.1^–^ cell fraction, we gated on TCRgd^+^ cells to define the γδ T cell population; flow cytometric quantification revealed that these cells constituted only a minimal proportion, with no significant age-related difference detected (**Supplementary Figure 4b**). After gating out γδ T cells, CD4 and CD8 markers were used to distinguish T cell subsets (**Supplementary Figure 4c**). We found that the proportion of CD4^+^CD8^+^ double-positive T cells (DPT) in mouse livers more than doubled in aged mice, increasing from 0.1240% to 0.2733%. Although the results did not reach statistical significance, the increase in DPT cells has been associated with the pathogenesis of various liver diseases (38, 39). Despite the lack of significant variation in the percentage of CD4^+^ T cells, multiple CD4^+^ T cell subsets exhibited a marked and significant elevation in aged livers (**Figures 2a–d**; **Supplementary Figures S5a**, **S6a–b**). Th2 and Treg cells represented the major components of the hepatic CD4^+^ T cell pool, and the expansion of these subsets during liver aging may lead to a shift toward heightened immune tolerance in the hepatic microenvironment. The proportions of Th1 and Th2 cells showed no significant difference in aged livers (**Supplementary Figure 5c**). T follicular helper (Tfh) cells were also detected in the liver, with statistical significance observed in aged livers (**Supplementary Figure 5b**). Therefore, subsets of T cells increased in the senescent livers, which can contribute to the induction of hepatic immune suppression.

Statistical significance for **Figure 2** was defined as *p* < 0.05 and *p* < 0.01 representing different significance levels.

### 3.3 CD8^+^ T and DNT cell subsets are stable in the liver of aged mice

Based on gating for CD8^+^, the CD8^+^ T cells accounted for a relatively low proportion in mouse livers, increasing from 2% in young mice to 3.5% in aged mice, with no statistical significance observed (**Supplementary Figure 7a**). Subsequently, CD8^+^ T cells were further subdivided into effector, memory, and naive CD8^+^ T cells based on the expression (**Supplementary Figure 4b**). Among these subsets, the vast majority were effector CD8^+^ T cells, whose mean proportion increased from 1.122% in young mice to 2.540% in aged mice, with no statistically significant difference observed (**Supplementary Figure 7b**). Likewise, no statistically significant alterations were observed in the percentages of other CD8^+^ T cell subsets within the senescent liver (**Supplementary Figure 7**). Results from gating on CD4^–^CD8^–^ cells showed that the proportion of double-negative T (DNT) cells remained stable in aged livers (**Supplementary Figure 7c**). Based on CD25 and CD44 expression, four subsets of DNT cells were further gated, namely DNT1 (CD44^+^CD25^–^), DNT2 (CD44^+^CD25^+^), DNT3 (CD44^–^CD25^+^), and DNT4 (CD44^–^CD25^–^) (**Supplementary Figure 7d**). No statistically significant changes were observed in the four DNT cell subsets within the senescent liver (**Supplementary Figure 7**). Taken together, the proportions of CD8^+^ T cells, DNT cells, and their subsets were maintained at stable levels in the aging liver.

### 3.4 Enhanced pro-inflammatory phenotype of macrophages in aging livers

**Figure 3:**
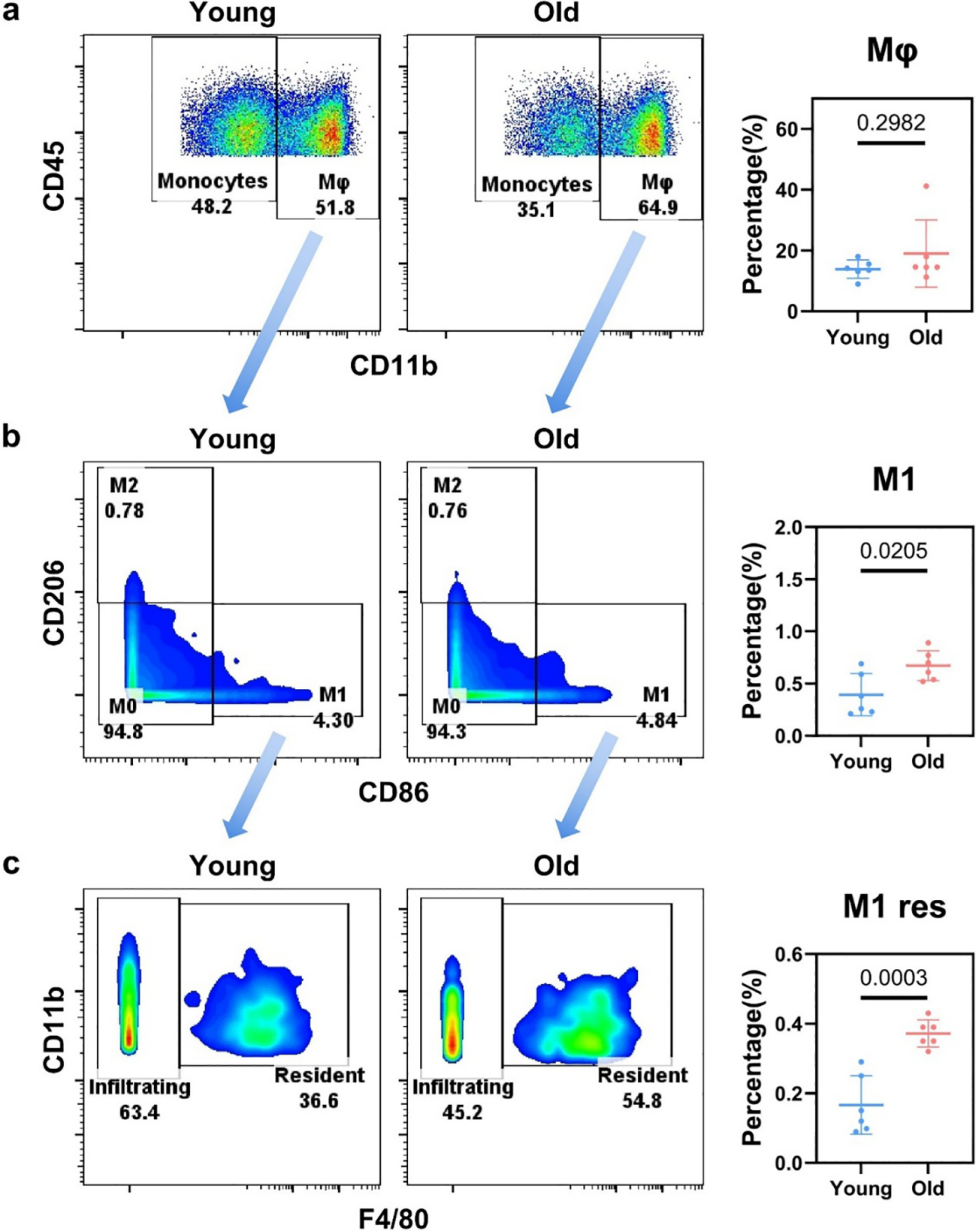
Flow cytometry gating for macrophage subsets in the liver of young and aged mice. **(a)** Flow cytometry plots (CD45 vs. CD11b gating) showing the distribution of Monocytes and Mφ (macrophage) populations in young and old mice, with quantification of Mφ percentage. **(b)** Flow cytometry plots (CD206 vs. CD86 gating) showing the distribution of M0, M1, and M2 macrophage subsets, with quantification of M1 subset percentage. **(c)** Flow cytometry plots (CD11b vs. F4/80 gating) showing the distribution of Infiltrating and Resident M1 (M1 res) populations, with quantification of M1 res percentage.

**Figure 4:**
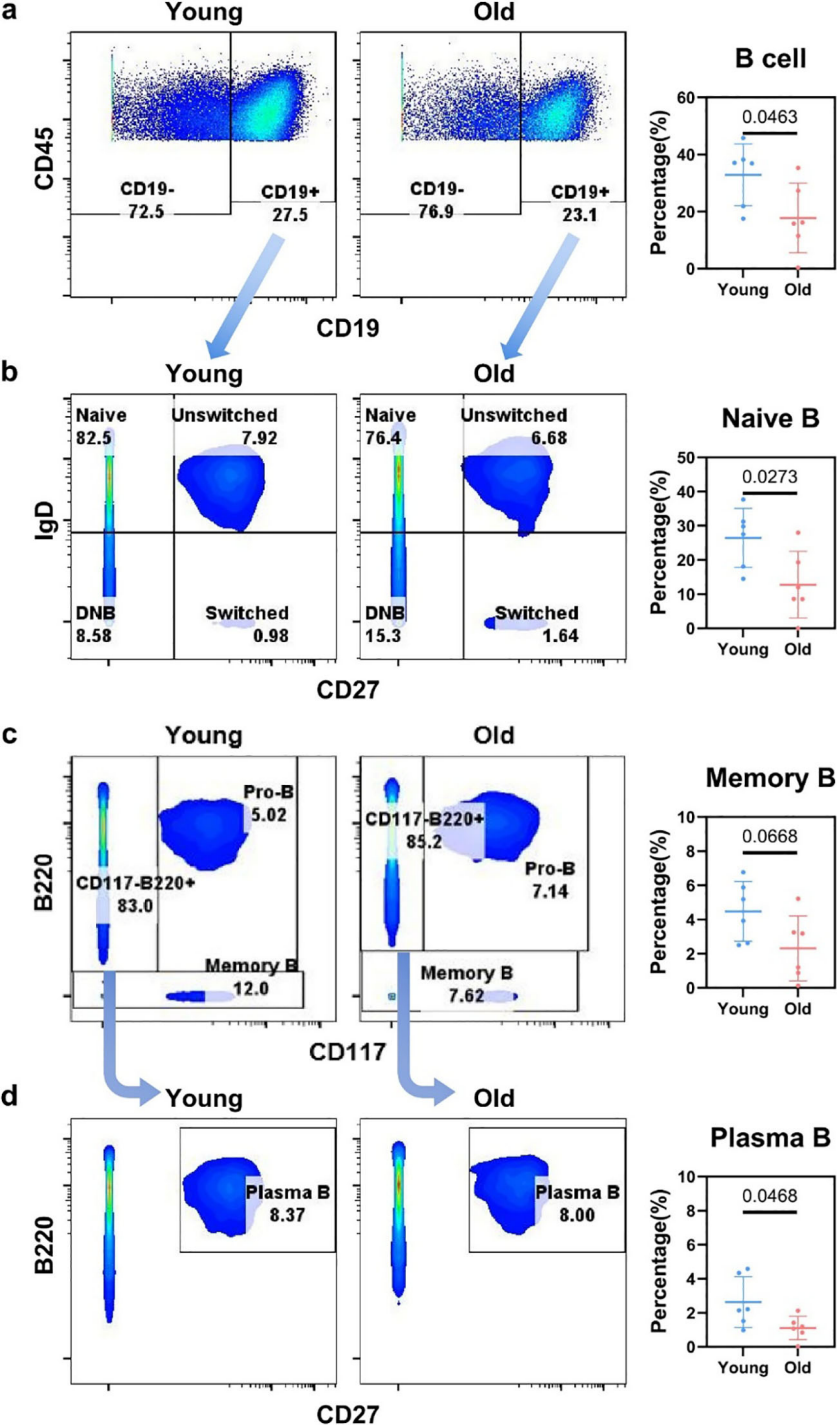
Flow cytometry gating for B cell subsets in the liver of young and aged mice. **(a)** Flow cytometry plots (CD45 vs. CD19 gating) showing the distribution of CD19^–^ and B cell (CD19^+^) populations, with quantification of B cell percentage. **(b)** Flow cytometry plots (IgD vs. CD27 gating) showing the distribution of Naive B, Unswitched B, Switched B, and DNB subsets, with quantification of Naive B cell percentage. **(c)** Flow cytometry plots (B220 vs. CD117 gating) showing the distribution of CD117^–^B220^+^, Pro-B, and Memory B subsets, with quantification of Memory B cell percentage. **(d)** Flow cytometry plots (B220 vs. CD27 gating) showing the distribution of B cell subsets and Plasma B population, with quantification of Plasma B cell percentage.

For the gating of myeloid cells, infiltrating neutrophils were first identified based on Ly6G positivity (**Supplementary Figure 9a**). There was no significant change in the proportion of neutrophils in the liver between young and aged mice. From the Ly6G^–^ subset, conventional type 1 dendritic cells (cDC1) and conventional type 2 dendritic cells (cDC2) were sequentially gated based on CD8α and CD4 expression; plasmacytoid dendritic cells (pDC) were further isolated via gating with CD317 (**Supplementary Figures 9b–d**). Among all dendritic cell subsets, only cDC2 exhibited a statistically significant increase with aging (**Supplementary Figures 9b–d**). As a key antigen-presenting cell, its core functions include expressing costimulatory molecules to activate naive CD4^+^ T cells, and driving Th2 cell differentiation (40). Accordingly, the upregulated cDC2 subset may contribute to the elevated proportion of Th2 cells in aged livers. Subsequent to dendritic cell profiling, monocytes and macrophages were isolated via gating according to differential CD11b expression level, defined as CD11b^low^and CD11b^high^ populations, respectively (**Figure 3a**). The results showed that the proportions of monocytes and macrophages did not undergo significant changes in the aging liver (**Figure 3a**; **Supplementary Figure S10**). Thereafter, further gating was conducted to distinguish the three macrophage phenotypes (M0, M1, M2) according to CD86 and CD206 expression profiles (**Figure 3b**; **Supplementary Figure S10**). Statistical analyses revealed that the proportion of M1 macrophages increased significantly from 0.3933% to 0.6717% (**Figure 4b**). Further, to clarify the origin of M1 macrophages, we conducted F4/80-mediated gating to differentiate resident macrophages (res) from those of monocytic origin (**Figure 3c**). Unexpectedly, in contrast to established notions, the increase in M1 macrophages originated from resident macrophages instead of monocytic precursors (**Figure 3c**; **Supplementary Figure S10**). Overall, with the exception of the increased proportions of two low-abundance subsets—M1 macrophages and cDC2—the composition of myeloid cell subsets remained largely stable during senescence.

Statistical significance for **Figure 3** was defined as *p* < 0.05, *p* < 0.01 and *p* < 0.001 representing different significance levels.

### 3.5 B cell subsets show reduced enrichment in the livers of aged mice

Next, we gated on CD19-positive B cells from the CD3-negative cell subset. As previously described, B cells represented the sole major cell type with a statistically significant reduction (**Figures 1c**, **4a**). Based on the expression of IgD and CD27, B cells were further classified into four subsets, namely naive B cells, unswitched memory B cells, switched memory B cells, and double-negative B cells (**Figure 4b**). We found that IgD+CD27- B cells decreased significantly from 26.47% in young mice to 12.75% in aged mice (**Figure 4b**). In previous studies, naive B cells were thought to be predominantly localized in lymphoid tissues and organs, with little to no mention of their presence in the liver. Naive B cells are defined as a subset of quiescent B lymphocytes that have not been exposed to antigens, expressing surface IgD molecules but lacking markers such as CD27 (41). Previous studies have not reported the presence of naive B cells in the liver. These naive B cells residing in the liver may contribute to the liver’s barrier function, whereas their marked reduction in the aging liver may indicate an increased risk of infection. While the populations of naive B cells and unswitched B cells were reduced, no alterations were observed in the numbers of other B cell subsets (**Supplementary Figure 11**). We further gated pro-B cells, memory B cells, and plasma B cells; however, the numbers of these cells did not increase but instead showed a decreasing trend (**Figures 4c, d**; **Supplementary Figure S11**). Collectively, the hepatic B cell compartment—especially naive B cells—underwent a marked proportional decline, which resulted from a numerical reduction instead of phenotypic conversion to alternative B cell subsets.

Statistical significance for **Figure 4** was defined as *p* < 0.05.

### 3.6 IgD represents a defining feature of hepatic B cells, with its expression declining progressively with age

**Figure 5:**
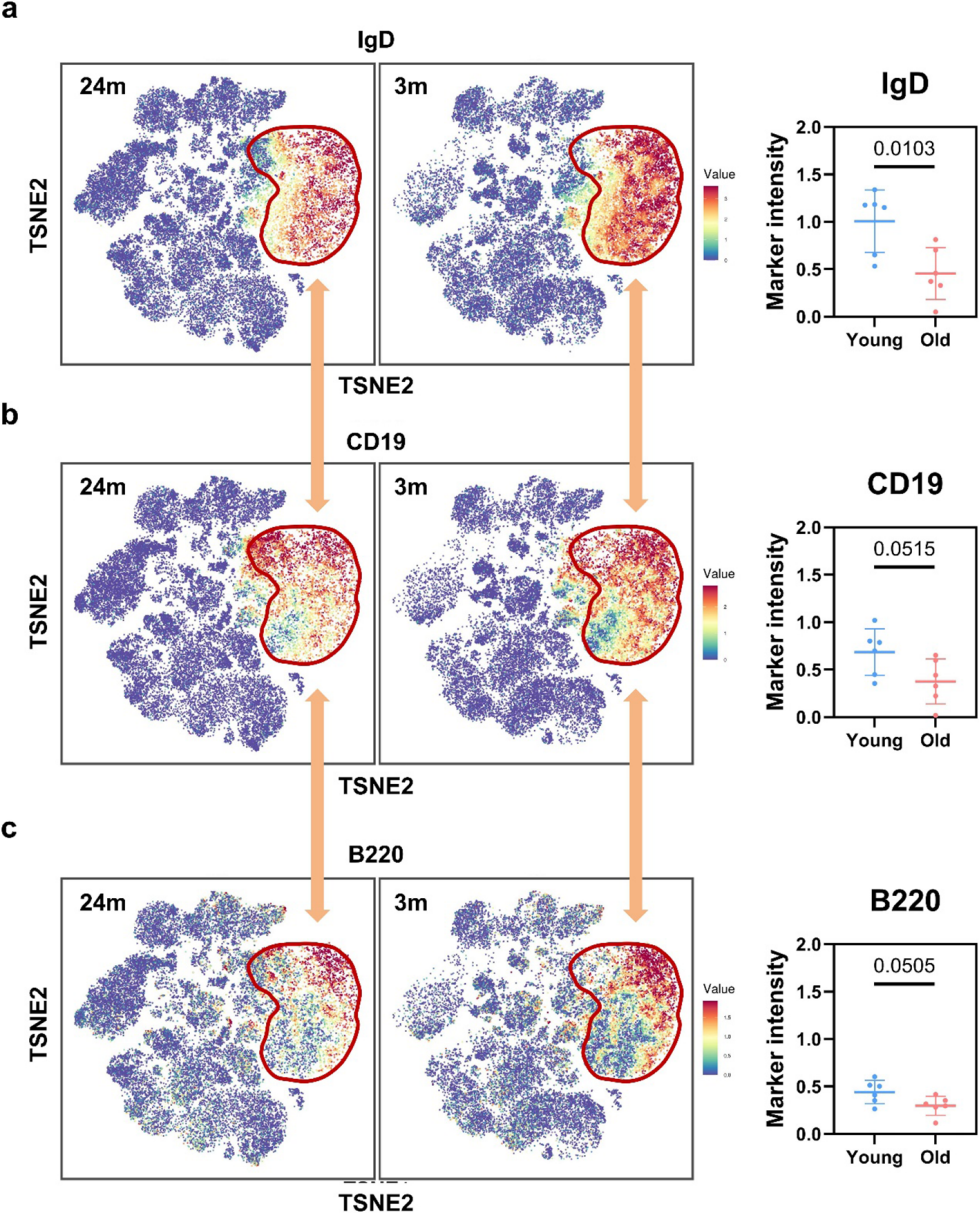
t-SNE-based analysis and quantification of IgD, CD19, and B220 expression in hepatic B cells. **(a)** TSNE plots (left) show IgD expression intensity (blue = low, red = high) in gated B cells (red boundary) from young (3m) and aged (24m) murine livers; the right panel quantifies IgD intensity (box plot: median/range) between groups. **(b)** TSNE plots (left) display CD19 expression intensity (blue = low, red = high) in gated B cells (red boundary) from young (3m) and aged (24m) murine livers; the right panel quantifies CD19 intensity (box plot: median/range) between groups. **(c)** TSNE plots (left) illustrate B220 expression intensity (blue = low, red = high) in gated B cells (red boundary) from young (3m) and aged (24m) murine livers; the right panel quantifies B220 intensity (box plot: median/range) between groups.

To uncover additional cellular phenotypic information, t-SNE dimensionality reduction analysis was applied to our CyTOF data, thereby providing a more straightforward visualization of key liver-resident cell subsets (**Supplementary Figure 12a**). Through t-SNE dimensionality reduction and clustering analysis, we resolved 29 immune cell subsets; notably, clusters C02 to C09 exhibited robust CD19 expression and were thus designated as B cell populations (**Supplementary Figures 12a–b**). IgD was highly expressed across all B cell subsets in the liver (**Supplementary Figure 12b**), and its expression level exceeded that of CD19; this high expression pattern was validated by both t-SNE analysis and manual gating strategies (**Figures 5a, b**; **Supplementary Figures S12b–c**). In addition to IgD, we also identified the co-expression of B220 in hepatic B cells, albeit with lower relative expression levels to IgD (**Figures 5a–c**; **Supplementary Figures S12b–d**). IgD and B220, but not CD19, were significantly downregulated in aged liver (**Figures 5a–c**). Results further demonstrated that the reduction in IgD expression was statistically more pronounced than that of B220, and IgD^+^ cells accounted for a larger proportion of the total B cell in t-SNE plots (**Figures 5a-c**). Furthermore, we detected IgD and IgG levels using ELISA, and the results showed that IgD levels in the liver of aged mice were significantly lower than those in young mice (**Supplementary Figure 14**). Meanwhile, hepatic IgG levels showed increased concentration, suggesting a potential transition of IgD-expressing naive B cells to IgG-expressing mature B cells in the aging liver. Our findings demonstrate that IgD serves as the primary characteristic marker of liver B cells, and the attenuation of this marker in aged livers is likely indicative of hepatic B cell dysfunction.

Statistical significance for **Figure 5** was defined as *p* < 0.05.

### 3.7 Altered gene expression is observed in the hepatic B cell during aging

**Figure 6:**
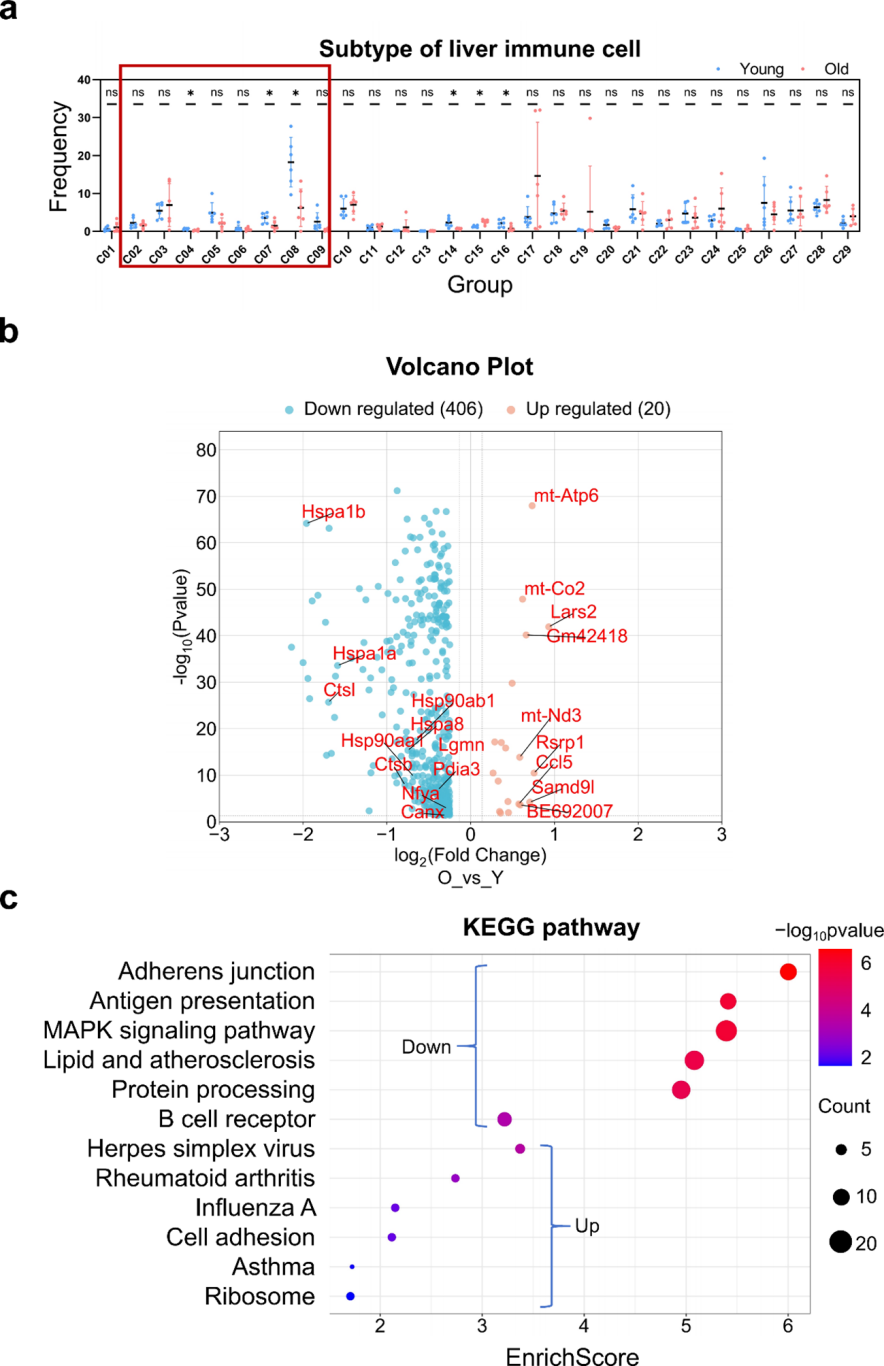
Analysis of hepatic immune cell subtype frequency, differential molecules and KEGG pathway enrichment. **(a)** Frequency of liver immune cell subtypes. Dot plot illustrating the frequency of 29 liver immune cell subtypes (C01–C29) across experimental groups. The red box demarcates B subtypes C02–C09 as B cell subsets. *: p < 0.05, statistically significant difference. ns: no significant difference. **(b)** Volcano plot depicting differential molecular abundance. 406 blue points correspond to downregulated molecules and 20 red points correspond to upregulated molecules. Select prominent molecules (e.g., Hspa1b, Hspa1a) are annotated for clarity. **(c)** KEGG pathway enrichment analysis. Pathways are ordered by EnrichScore; dot color intensity indicates -log^10^ p-value (red = higher value; blue = lower value), and dot size denotes the number of genes associated with each pathway. Blue brackets indicate pathways enriched in downregulated or upregulated molecules.

To characterize changes in B cell subsets identified via t-SNE analysis, we compared the proportional shifts of all immune cell subsets in the livers of aged subjects. T-SNE-based dimensionality reduction distinguished 29 cell subsets, with B cell subsets displaying the most significant alterations, which corroborated the outcomes of our manual gating (**Figure 6a**; **Supplementary Figures S12a–b**). Both manual gating and t-SNE dimensionality reduction analysis revealed a reduced proportion of B cells in aged livers, suggesting impairment of hepatic B cell subsets (**Figure 4a**, **6a**). Therefore, we investigated whether hepatic B cells in aged mice undergo other alterations in addition to changes in their proportion. We analyzed the differentially expressed genes (DEGs) of B cells from previously published single-cell database of livers from aged mice (42). Consistent with our speculation, B cells in the aging liver are predominantly characterized by reduced gene expression, with 406 genes downregulated and only 20 genes upregulated (**Figure 6b**). We further investigated the classification of DEGs in hepatic B cells from aged livers. KEGG pathway analysis revealed that the downregulated DEGs in hepatic B cells from aged livers were correlated with antigen presentation, protein processing, and B cell receptor, among which the DEGs involved in antigen presentation were visualized in the volcano plot (**Figures 6b, c**). In contrast, the number of upregulated DEGs was too small to be enriched in signaling pathways (**Figure 6c**). Based on KEGG pathway analysis, antigen presentation, protein processing in the endoplasmic reticulum, and B cell receptor signaling pathways were identified as the most significantly enriched terms, implying potential regulatory roles in the alterations of hepatic B cells during aging.

Statistical significance for **Figure 6** was defined as *p* < 0.05.

## 4 Discussion

The liver, as a pivotal organ integrating metabolic functions and immune defense, harbors a sophisticated immune landscape that undergoes profound remodeling during aging. This study employed high-dimensional single-cell mass cytometry to systematically dissect age-related alterations in hepatic immune cell subsets, uncovering novel insights into the mechanisms underlying age-associated liver immune dysfunction. Our findings identify impaired B cell subsets, reshaped CD4^+^ T cell subsets and enhanced pro-inflammatory macrophage polarization as prominent features of hepatic immune aging in mice, providing a basis for developing interventions for age-related liver diseases.

Our data demonstrate that liver-resident IgD+ B cells account for the dominant B cell subset in young livers. The ~50% reduction in naive B cells in aged livers, along with the decreasing trend in memory B cells and plasma B cells, indicates a systemic impairment of the hepatic B cell lineage rather than phenotypic conversion to other subsets. Alternatively, AGING ATLAS single-cell sequencing data demonstrated B cell signal transduction pathway enrichment in aged livers, with downregulated genes enriched in cell migration and receptor binding pathways. Reduced naive B cells may impair hepatic immunity to gut pathogens, while the potential B cell dysfunction could disrupt tolerance and homeostasis.

The hepatic T cell landscape also exhibits age-related remodeling. Th1, Th2, Th17, and Treg cells were significantly increased in aged livers. Th2 and Treg cells, as major components of the hepatic CD4^+^ T cell pool, play critical roles in mediating immune tolerance. Their expansion may shift the hepatic microenvironment toward heightened tolerance, potentially limiting excessive inflammatory responses but also impairing anti-tumor and anti-pathogen immunity. Additionally, the DPT cells, although not statistically significant, are noteworthy given their association with the pathogenesis of chronic liver diseases such as hepatitis B and C. In contrast, CD8^+^ T cells and DNT cells, along with their subsets, remained stable during aging, indicating a selective remodeling of T cell populations rather than global dysregulation. This specificity suggests that distinct T cell subsets may have divergent roles in age-related liver pathology, with CD4^+^ T cell subsets emerging as key mediators of immune microenvironment alterations.

The myeloid cell compartment, particularly macrophages, exhibited enhanced pro-inflammatory phenotypes in aged livers. While the overall proportions of monocytes and macrophages did not change significantly, the proportion of M1 pro-inflammatory macrophages (CD86^+^CD206^–^) increased significantly, originating from resident macrophages rather than monocytic infiltrates—contrary to the conventional view that inflammatory macrophages in aging tissues are primarily derived from circulating monocytes. This finding implies that intrinsic activation of liver-resident macrophages (Kupffer cells) drives age-related hepatic inflammation. Concurrently, cDC2 were the only dendritic cell subset that increased with aging. As key antigen-presenting cells that promote Th2 cell differentiation, the expansion of cDC2 may contribute to the elevated Th2 cell proportion in aged livers, forming a pro-tolerance inflammatory loop. Notably, two aged mice exhibited extreme neutrophil infiltration (>30%), indicating overt local inflammation, which aligns with the role of M1 macrophages in recruiting neutrophils via pro-inflammatory cytokines. Collectively, the pro-inflammatory shift in myeloid cells—characterized by M1 macrophage accumulation and cDC2 expansion—creates a chronic inflammatory microenvironment that exacerbates liver damage and fibrosis during aging.

Several limitations inherent to the present study warrant acknowledgment. First, due to the limited number of mice employed in this study, as well as inherent species-specific disparities in immune cell composition and aging trajectories, results may not fully recapitulate human hepatic immune aging. Second, the precious aging liver samples have been fully used for mass cytometry, so future studies are needed to further investigate the causes of these cellular changes. Third, as the single-cell transcriptomic data may differ from those in the present study in terms of mouse strain background, exact age, conditions, tissue processing protocols, and sequencing pipelines, the results are not correlated with those of this study and are only provided for supplementary reference. Fourth, due to the limited sample size, some p-values hover near the 0.05 threshold, which may result in false positives and false negatives. We also detected ILCs in the liver, although none of these subsets exhibited alterations in aging (**Supplementary Figure 13**). Future studies should integrate single-cell RNA sequencing with functional assays to elucidate the transcriptional and functional dynamics of hepatic immune cells during aging, and validate key findings in human liver samples.

In conclusion, our study delineates the remodeling landscape of hepatic immune cells during aging, and characterizes the age-associated alterations in B cell, CD4^+^ T cell and macrophage subsets in the murine liver. These alterations collectively disrupt hepatic immune homeostasis, increasing the risk of chronic liver diseases in older individuals. These results may provide support for future studies on liver aging and its associated immune changes. Further investigation into the molecular mechanisms underlying these immune alterations will deepen our understanding of liver aging and facilitate the development of interventions for older adults at high risk of liver disease.

## Data Availability

The datasets presented in this study can be found in online repositories. The names of the repository/repositories and accession number(s) can be found in the article/**Supplementary Material**.
