# Supplementary material for: Single-cell CyTOF profiling reveals alterations in B, T and macrophage subsets during murine hepatic aging
Source: Front Immunol. 2026 Apr 28;17:1787641. doi: 10.3389/fimmu.2026.1787641 (PMC13160732; doi:10.3389/fimmu.2026.1787641)
Supplement: Supplementary file 1 [file DataSheet1.docx]

Table S1 Antibody information

| **List** | **Label** | **marker** | **clone** | **dilution** | **Brand** | **Catalog number** |
| --- | --- | --- | --- | --- | --- | --- |
| **1** | **89Y** | **CD45** | **30-F11** | **400** | **Biolegend** | **103102** |
| **2** | **115ln** | **CD3ε** | **145-2C11** | **50** | **Biolegend** | **100302** |
| **3** | **141Pr** | **CD192(CCR2)** | **475301** | **100** | **R&D** | **MAB55381-100** |
| **4** | **142Nd** | **CD11c** | **N418** | **100** | **Biolegend** | **117302** |
| **5** | **143Nd** | **CD172a(SIRPα）** | **P84** | **100** | **Biolegend** | **144002** |
| **6** | **144Nd** | **CD117(c-kit)** | **2B8** | **200** | **Biolegend** | **105802** |
| **7** | **145Nd** | **CD69** | **H1.2F3** | **100** | **Biolegend** | **104502** |
| **8** | **146Nd** | **CD27** | **LG.3A10** | **100** | **Biolegend** | **124202** |
| **9** | **147Sm** | **CD86** | **GL-1** | **200** | **Biolegend** | **105002** |
| **10** | **148Nd** | **Ly-6C** | **HK1.4** | **800** | **Biolegend** | **128002** |
| **11** | **149Sm** | **CX3CR1** | **SA011F11** | **400** | **Biolegend** | **149002** |
| **12** | **150Nd** | **CD25** | **3C7** | **50** | **Biolegend** | **101902** |
| **13** | **151Eu** | **CD44** | **IM7** | **100** | **Biolegend** | **103002** |
| **14** | **152Sm** | **CD19** | **6D5** | **400** | **Biolegend** | **115502** |
| **15** | **153Eu** | **IgD** | **11-26c.2a** | **400** | **Biolegend** | **405702** |
| **16** | **154Sm** | **CD62L** | **MEL-14** | **200** | **Biolegend** | **104402** |
| **17** | **155Gd** | **CD103** | **2E7** | **200** | **Biolegend** | **121402** |
| **18** | **156Gd** | **CD194(CCR4)** | **2G12** | **100** | **Biolegend** | **131202** |
| **19** | **157Gd** | **CD39** | **5F2** | **50** | **Biolegend** | **135702** |
| **20** | **158Gd** | **CD45R(B220)** | **RA3-6B2** | **200** | **Biolegend** | **103202** |
| **21** | **159Tb** | **F4/80** | **Cl:A3-1** | **400** | **Biorad** | **MCA497G** |
| **22** | **160Gd** | **CD206(MMR)** | **C068C2** | **100** | **Biolegend** | **141702** |
| **23** | **161Dy** | **CD64(FcγRI)** | **X54-5/7.1** | **100** | **Biolegend** | **139302** |
| **24** | **162Dy** | **CD183(CXCR3)** | **CXCR3-173** | **200** | **Biolegend** | **126502** |
| **25** | **163Dy** | **Ly-6G** | **1A8** | **800** | **Biolegend** | **127602** |
| **26** | **164Dy** | **CD163** | **S15049I** | **50** | **Biolegend** | **155302** |
| **27** | **165Ho** | **FOXP3** | **FJK-16s** | **50** | **eBioscience** | **14-5773-82** |
| **28** | **166Er** | **TCR γ/δ** | **GL3** | **100** | **Biolegend** | **118140** |
| **29** | **167Er** | **CD49b(pan-NK cells)** | **DX5** | **200** | **Biolegend** | **108902** |
| **30** | **168Er** | **CD317(BST-2)** | **44E9R** | **800** | **R&D** | **MAB8660** |
| **31** | **169Tm** | **TCR β chain** | **H57-597** | **400** | **Biolegend** | **109202** |
| **32** | **170Er** | **CD161(NK-1.1)** | **PK136** | **800** | **Biolegend** | **108702** |
| **33** | **171Yb** | **CD279(PD-1)** | **29F.1A12** | **200** | **Biolegend** | **135202** |
| **34** | **172Yb** | **CD127(IL-7Rα)** | **A7R34** | **100** | **Biolegend** | **135002** |
| **35** | **173Yb** | **CD278(ICOS)** | **C398.4A** | **200** | **Biolegend** | **313502** |
| **36** | **174Yb** | **CD196(CCR6)** | **29-2L17** | **800** | **Biolegend** | **129802** |
| **37** | **175Lu** | **Siglec-F** | **E50-2440** | **200** | **BD** | **552125** |
| **38** | **176Yb** | **MHC II(I-A/I-E)** | **M5/114.15.2** | **400** | **Biolegend** | **107602** |
| **39** | **197Au** | **CD4** | **RM4-5** | **800** | **Biolegend** | **100576** |
| **40** | **198pt** | **CD8a** | **53-6.7** | **400** | **Biolegend** | **100746** |
| **41** | **209Bi** | **CD11b** | **M1/70** | **800** | **Biolegend** | **101247** |


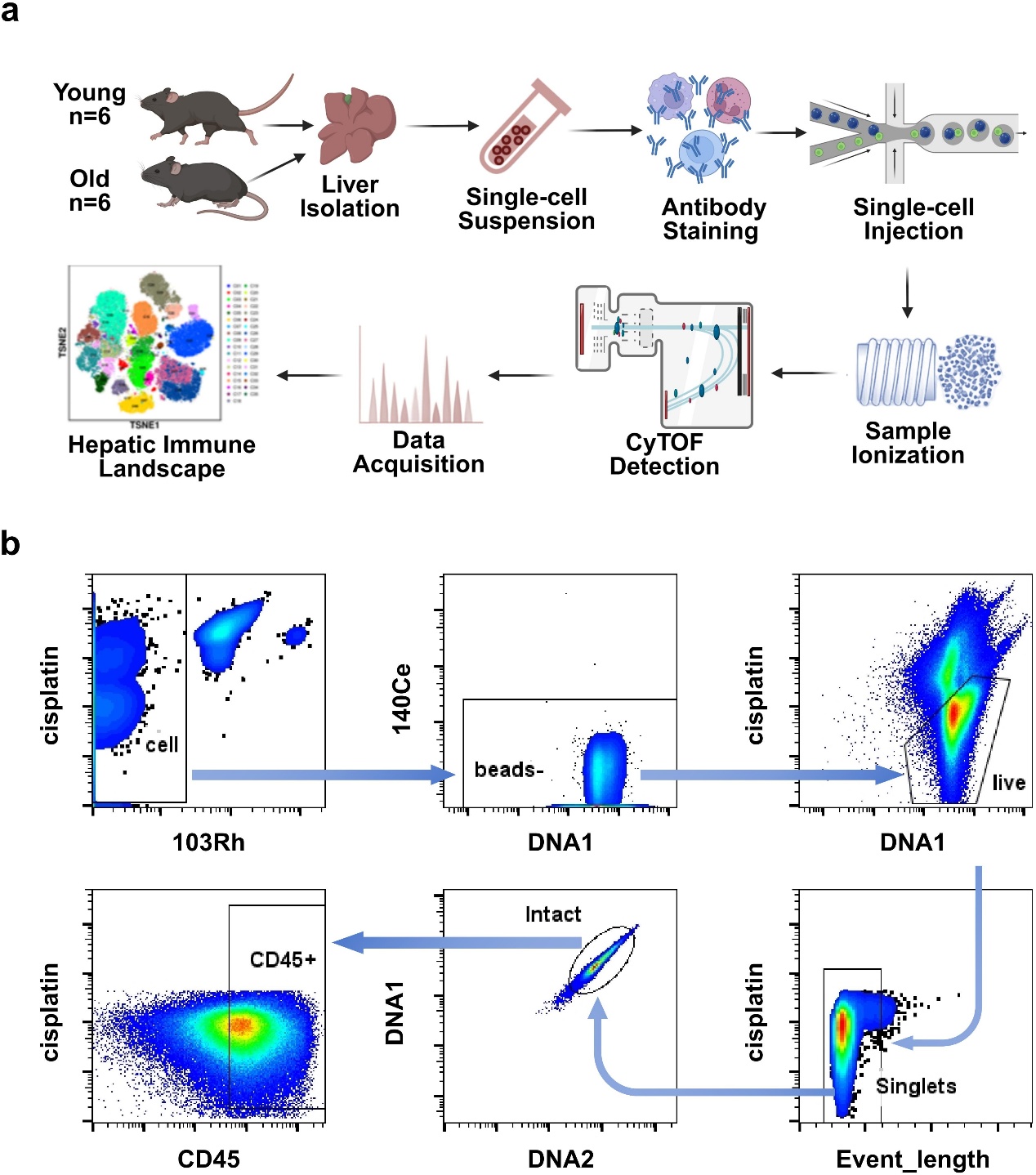


Fig. S1 Workflow of hepatic immune profiling by mass cytometry (CyTOF) and single-cell data quality control strategy.

(a) Schematic overview of the experimental pipeline for hepatic immune cell profiling. Liver tissues were isolated from young (3 months old, n=6) and aged (24 months old, n=6) mice, respectively. After processing into single-cell suspensions, the cells were stained with a panel of metal-conjugated antibodies targeting key surface and intracellular markers. The stained single-cell suspensions were then injected into the CyTOF instrument for ionization, detection, and high-dimensional data acquisition to characterize the hepatic immune landscape.

(b) Gating strategy for single-cell data quality control. Raw CyTOF data were sequentially filtered to exclude dead cells, debris, and residual non-cellular beads using signals from Cisplatin (internal standard for instrument normalization), Event_length (reflecting particle size and complexity), and a "beads-" gate. Intact cells were identified by positive DNA1 (a marker of cellular DNA content) signal, followed by exclusion of singlets and doublets to ensure cell population integrity. Finally, CD45⁺ immune cells were gated for subsequent subset analysis.


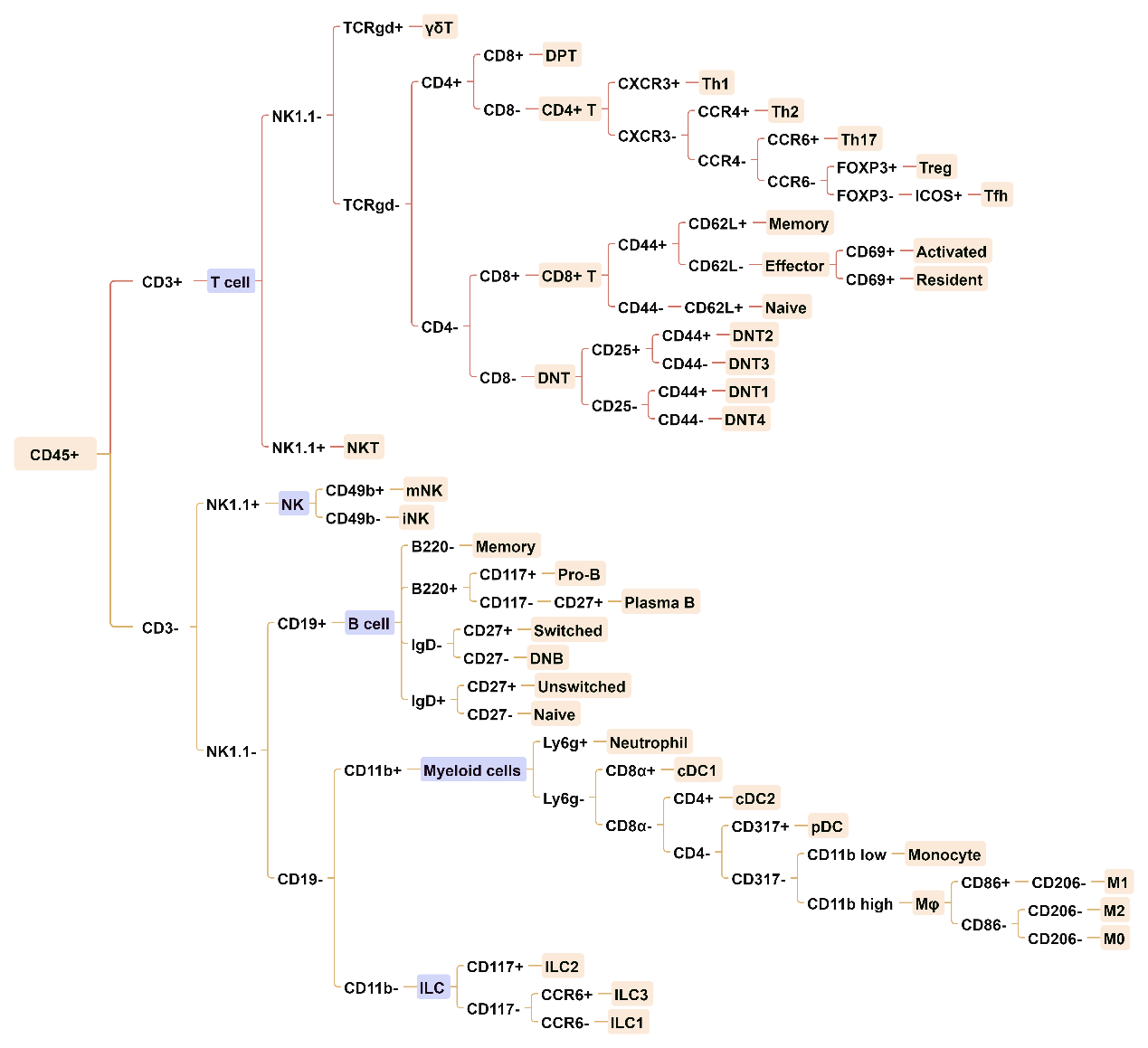


Fig. S2 Flow Cytometry Gating of Immune Cell Subtypes in Livers of Young vs. Old Mice.

The uncolored labels represent marker names, and the colored labels represent cell names: γδT (γδ T cell), DPT (Double-positive T cell), Th1 (T helper 1 cell), Th2 (T helper 2 cell), Th17 (T helper 17 cell), Treg (Regulatory T cell), Tfh (Follicular helper T cell), DNT1/2/3/4 (Double-negative T cell (subtypes 1/2/3/4)), NK (Natural killer cell), mNK (Mature natural killer cell), iNK (Immature natural killer cell), DNB (Double-negative B cell), Myeloid cells (Myeloid lineage cells), cDC (Conventional dendritic cell), pDC (Plasmacytoid dendritic cell), Mφ (Macrophage), M1 (M1-type macrophage), M2 (M2-type macrophage), M0 (M0-type macrophage), ILC (Innate lymphoid cell), and the nomenclature of other cells is presented in full spelling.


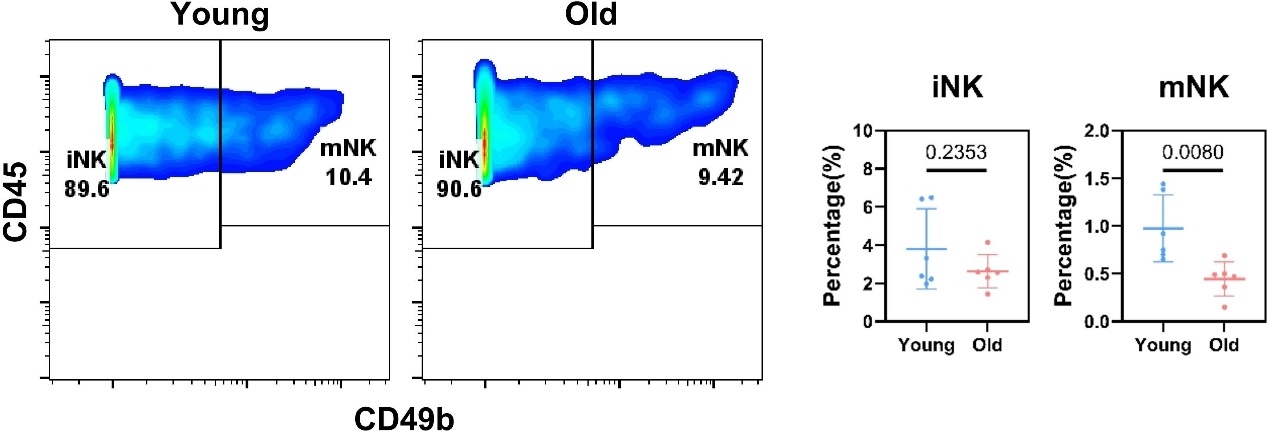


Fig. S3 Flow cytometry plots and quantification for differentiating mature (CD49b⁺) and immature (CD49b⁻) NK cell subsets among CD3⁻NK1.1⁺ cells in the livers of young and aged mice. Quantification data are presented as mean ±SD, and statistical significance for Fig. S3 was determined by unpaired Student’s t-test, with significance levels defined as *p* < 0.05 and *p* < 0.01 as indicated.


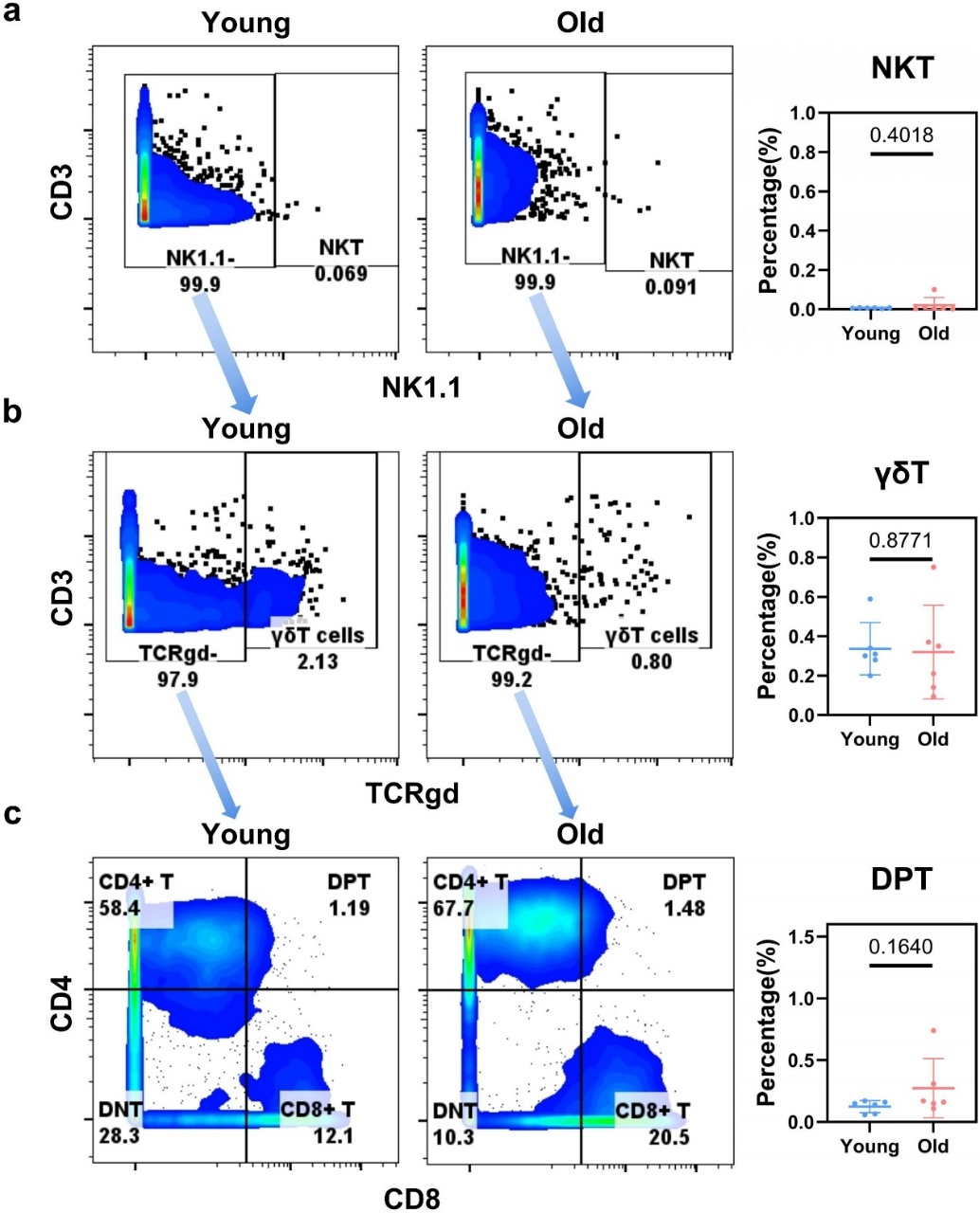


Fig. S4 Flow Cytometry Gating of CD3-Positive Cell Subtypes in Livers of Young vs. Old Mice

(a) Flow cytometry plots and quantification of NKT cell (CD3⁺NK1.1⁺) proportions within hepatic CD45⁺ immune cells in young and aged mice.

(b) Flow cytometry plots and quantification of γδT cell (CD3⁺NK1.1⁻TCRγδ⁺) proportions within hepatic CD45⁺ immune cells in young and aged mice.

(c) Flow cytometry plots and quantification of CD4⁺CD8⁺ double - positive T cell (DPT) proportions within hepatic CD3⁺NK1.1⁻TCRγδ⁻ CD45⁺ immune cells in young and aged mice.

Quantification data are presented as mean ± SD, and statistical significance for Fig. S4 was determined by unpaired Student’s t-test, with significance levels defined as *p* < 0.05 and *p* < 0.01 as indicated.


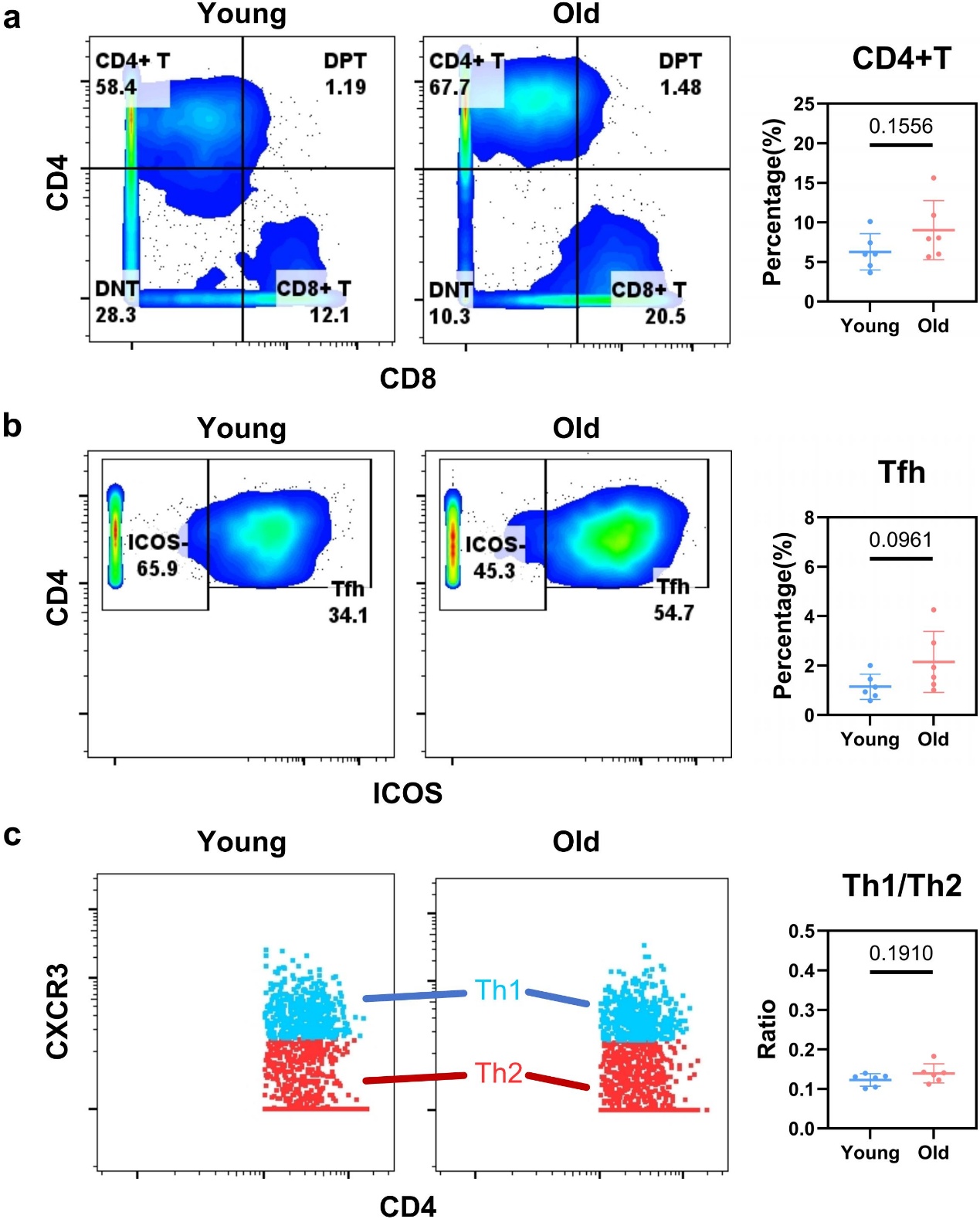


Fig. S5 Flow Cytometry Gating of CD4⁺ T Cells and Subtypes in Livers of Young vs. Old Mice

(a) Flow cytometry plots (CD4 vs. CD8 gating) showing the distribution of CD4⁺T, CD8⁺T, double-positive T (DPT), and double-negative T (DNT) cells in hepatic CD3⁺ immune cells from young and aged mice, with quantification of CD4⁺T cell proportions.

(b) Flow cytometry plots (CD4 vs. ICOS gating, within FOXP3⁻ hepatic CD4⁺T cells) identifying follicular helper T (Tfh) cells in young and aged mice, with quantification of Tfh cell proportions.

(c) Flow cytometry plots (CD4 vs. CXCR3 gating) distinguishing Th1 and Th2 subsets in hepatic CD4⁺T cells from young and aged mice, with quantification of the Th1/Th2 ratio.

Quantification data are presented as mean ± SD, and statistical significance for Fig. S5 was determined by unpaired Student’s t-test, with significance levels defined as *p* < 0.05 as indicated.


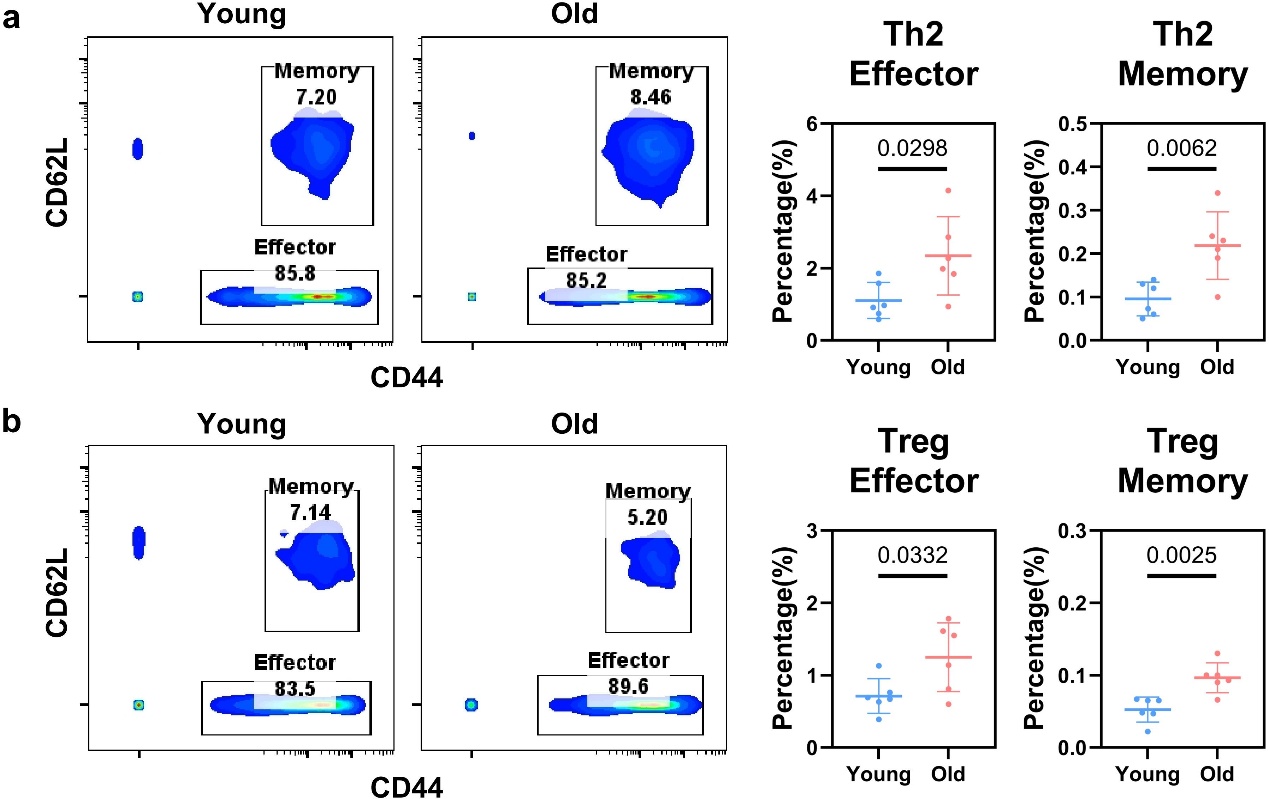


Fig. S6 Flow Cytometry Gating for Th2 cell and Treg cell subsets in the liver of young and aged mice

(a) Flow cytometry plots (CD44 vs. CD62L gating) showing the distribution of Effector (CD44⁺CD62L⁻) and Memory (CD44⁺CD62L⁺) subsets in Th2 cells from young and old mice, with quantification of Th2 Effector and Th2 Memory cell percentages.

(b) Flow cytometry plots (CD44 vs. CD62L gating) showing the distribution of Effector (CD44⁺CD62L⁻) and Memory (CD44⁺CD62L⁺) subsets in Treg cells from young and old mice, with quantification of Treg Effector and Treg Memory cell proportions.

Quantification data are presented as mean ± SD, and statistical significance for Fig. S6 was determined by unpaired Student’s t-test, with significance levels defined as *p* < 0.05 and *p* < 0.01 as indicated.


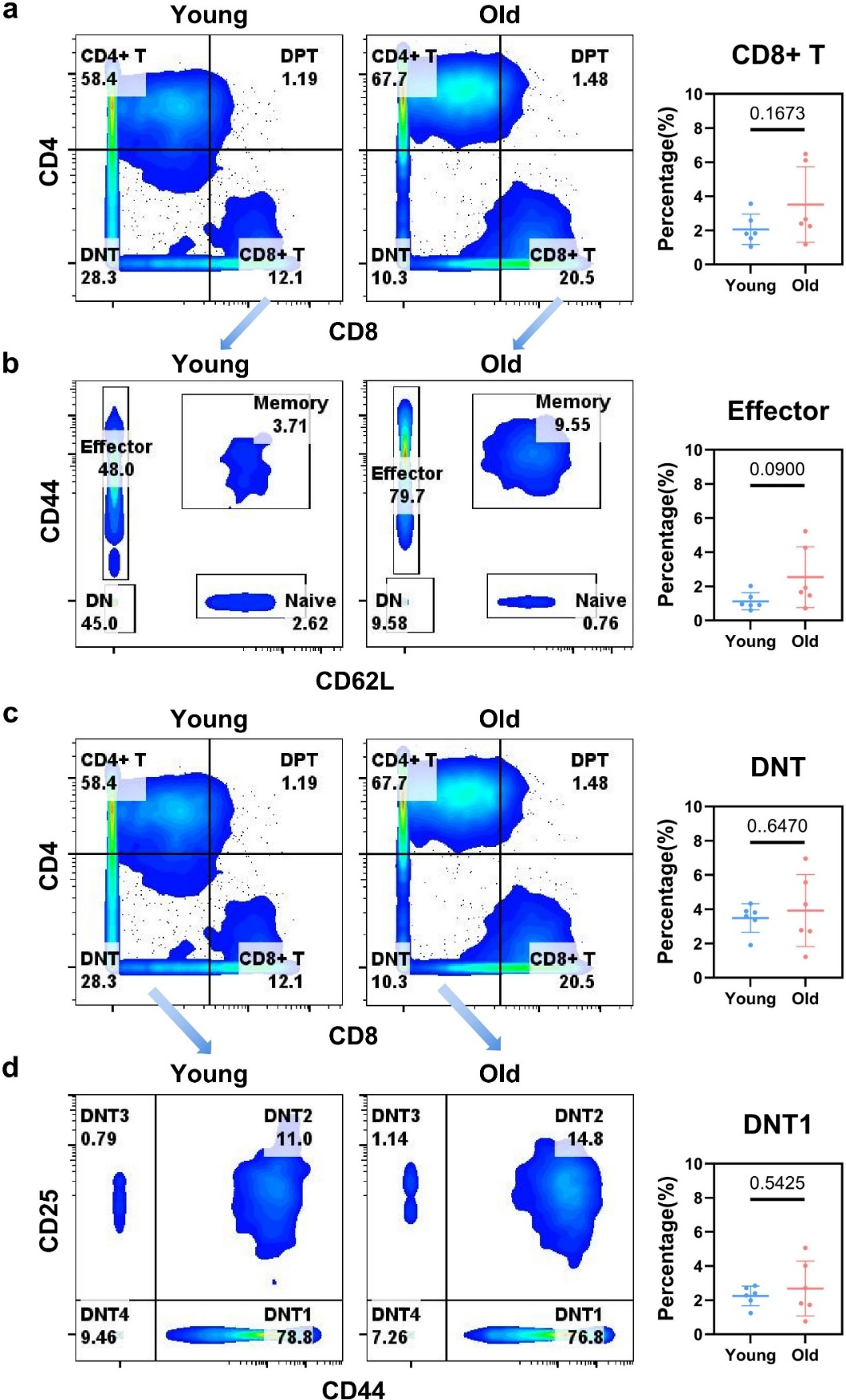


Fig. S7 Flow Cytometry Gating for CD8⁺ T and DNT Cell Subsets in the Liver of Young and Aged Mice

(a) Flow cytometry plots (CD4 vs. CD8 gating) showing the distribution of CD4⁺T, CD8⁺T, double-positive T (DPT), and double-negative T (DNT) cells in T cells from young and old mice, with quantification of CD8⁺T cell percentage.

(b) Flow cytometry plots (CD44 vs. CD62L gating) showing the distribution of Effector (CD44⁺CD62L⁻), Memory (CD44⁺CD62L⁺), Naive (CD44⁻CD62L⁺), and DN subsets in T cells from young and old mice, with quantification of Effector cell percentage.

(c) Flow cytometry plots (CD4 vs. CD8 gating) showing the distribution of CD4⁺T, CD8⁺T, DPT, and DNT cells in T cells from young and old mice, with quantification of DNT cell percentage.

(d) Flow cytometry plots (CD25 vs. CD44 gating) showing the distribution of DNT1, DNT2, DNT3, and DNT4 subsets in DNT cells from young and old mice, with quantification of DNT1 cell percentage.

Quantification data are presented as mean ± SD, and statistical significance for Fig. S7 was determined by unpaired Student’s t-test, with significance levels defined as *p* < 0.05 and *p* < 0.01 as indicated.


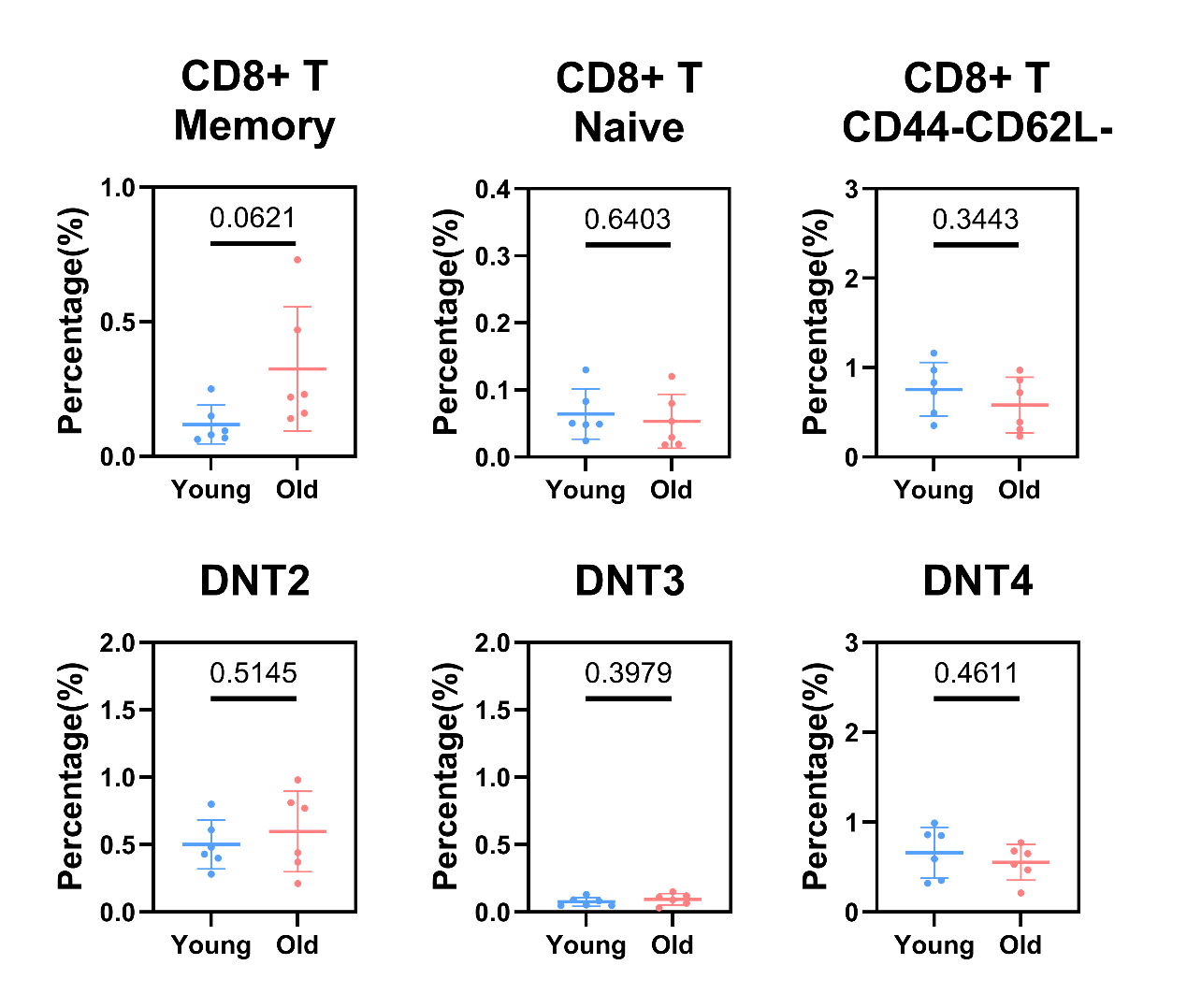


Fig. S8 CD8⁺ T and DNT Cell Subsets in the Liver of Young and Aged Mice. Quantification plots showing the percentage of (top row) CD8⁺ T cell subsets (Memory, Naive, CD44⁻CD62L⁻) and (bottom row) DNT subpopulations (DNT2, DNT3, DNT4) in young and old groups.

Quantification data are presented as mean ± SD, and statistical significance for Fig. S8 was determined by unpaired Student’s t-test, with significance levels defined as *p* < 0.05 and *p* < 0.01 as indicated.


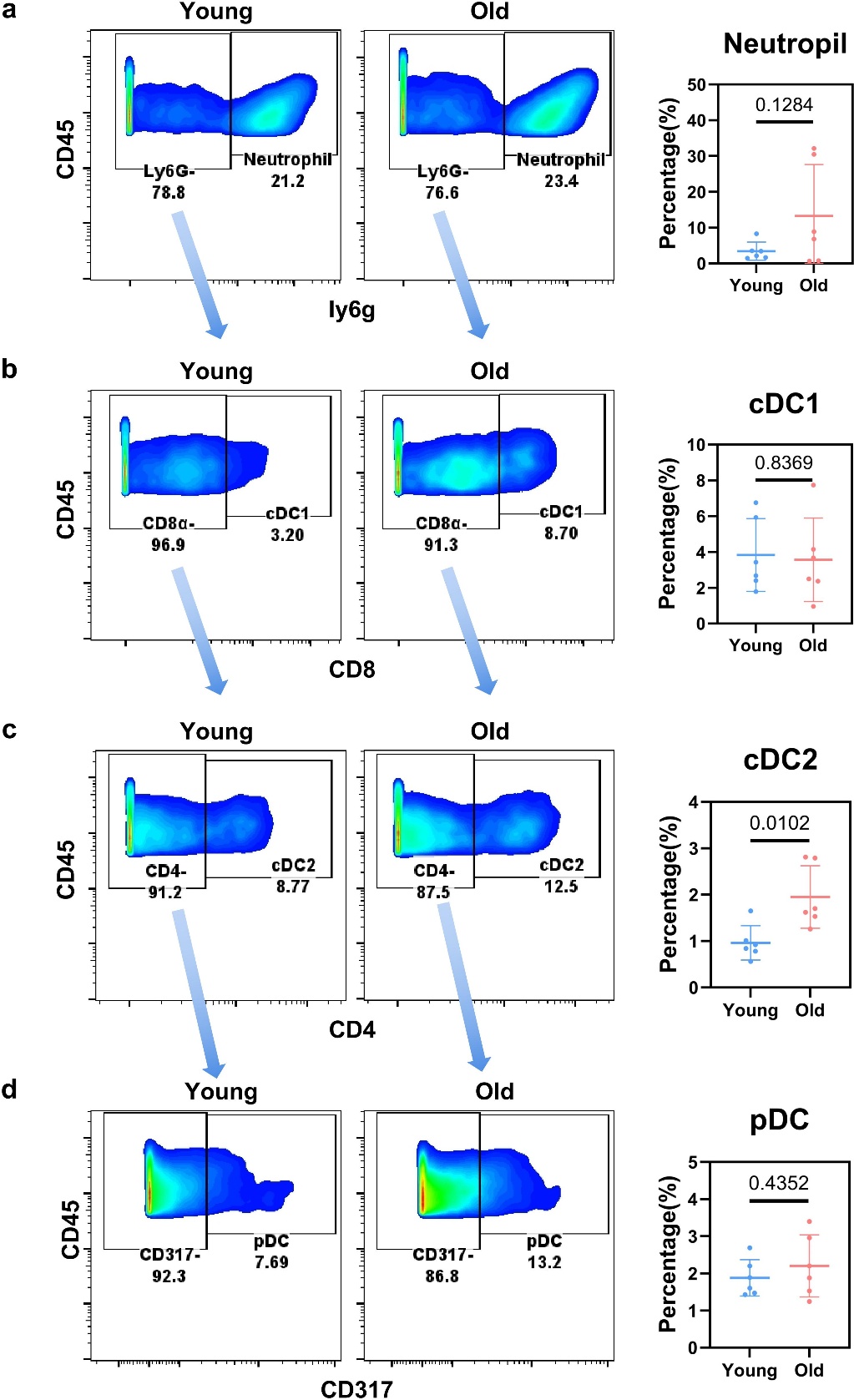


Fig. S9 Flow Cytometry Gating for Neutrophil and Dendritic Cell Subsets in the Liver of Young and Aged Mice

(a) Flow cytometry plots (CD45 vs. Ly6G gating) showing the distribution of Ly6G⁻ and Neutrophil (Ly6G⁺) populations in young and old mice, with quantification of Neutrophil percentage.

(b) Flow cytometry plots (CD45 vs. CD8α gating) showing the distribution of CD8α⁻ and cDC1 (CD8α⁺) populations in young and old mice, with quantification of cDC1 percentage.

(c) Flow cytometry plots (CD45 vs. CD4 gating) showing the distribution of CD4⁻ and cDC2 (CD4⁺) populations in young and old mice, with quantification of cDC2 percentage.

(d) Flow cytometry plots (CD45 vs. CD317 gating) showing the distribution of CD317⁻ and pDC (CD317⁺) populations in young and old mice, with quantification of pDC percentage.

Quantification data are presented as mean ± SD, and statistical significance for Fig. S9 was determined by unpaired Student’s t-test, with significance levels defined as *p* < 0.05 and *p* < 0.01 as indicated.


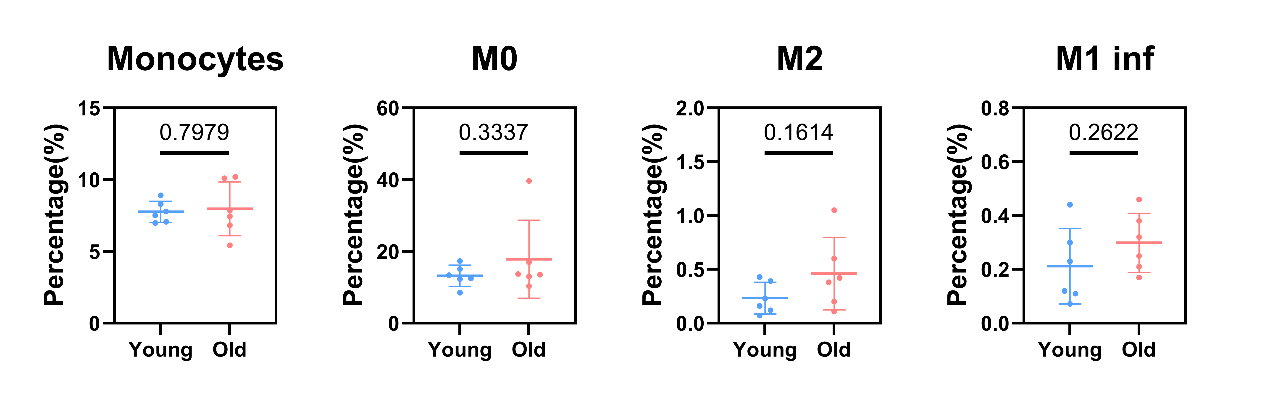


Fig. S10 Monocytes and Macrophage Subsets analysis in the Liver of Young and Aged Mice

Quantification plots showing the percentage of immune cell subsets (Monocytes, M0 macrophage, M2 macrophage, M1 infiltrating (M1 inf)) in young and old groups.

Quantification data are presented as mean ± SD, and statistical significance for Fig. S10 was determined by unpaired Student’s t-test, with significance levels defined as *p* < 0.05 and *p* < 0.01 as indicated.


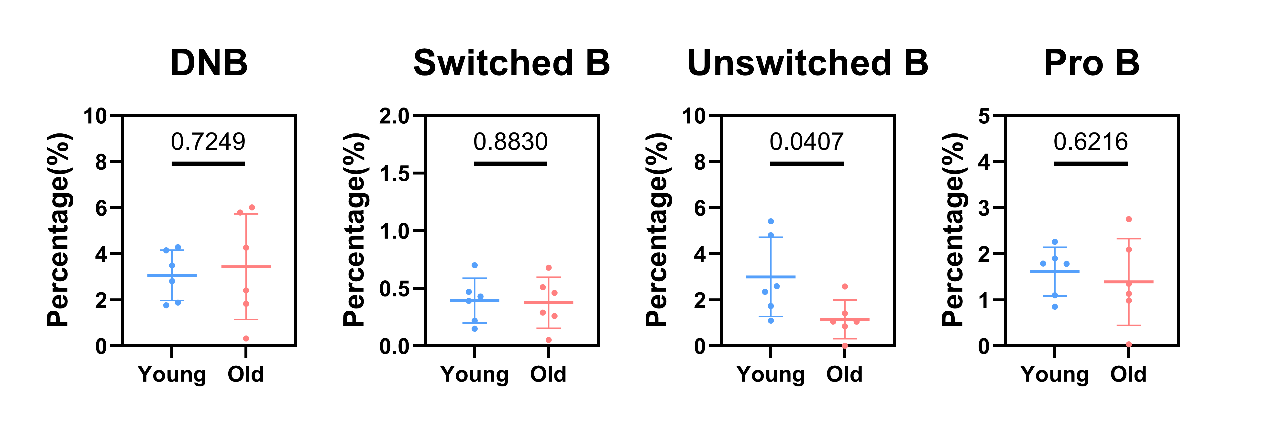


Fig. S11 B Cell Subsets analysis in the Liver of Young and Aged Mice. Quantification plots showing the percentage of B cell subsets (DNB, Switched B, Unswitched B, Pro B) in young and old groups.

Quantification data are presented as mean ± SD, and statistical significance for Fig. S10 was determined by unpaired Student’s t-test, with significance levels defined as *p* < 0.05 as indicated.


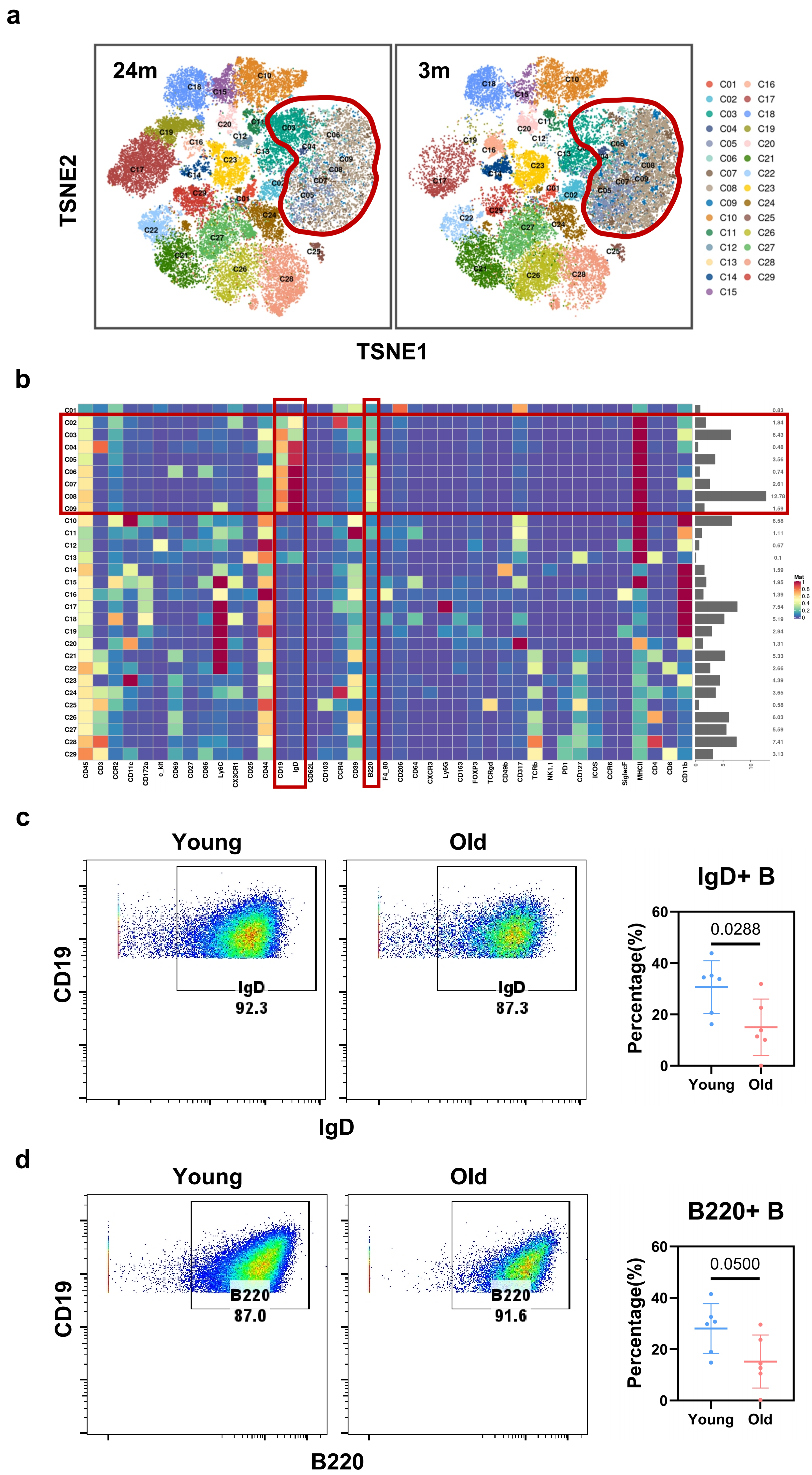


Fig. S12 t-SNE-Based Analysis of Immune Cell Subsets in Livers of Young and Aged Mice

(a) t-SNE plots showing the distribution of 29 cell clusters (C01–C29) in 24m (left) and 3m (right) groups. Red dashed line highlights a cluster-enriched region, with each color representing a distinct cell cluster.

(b) Heatmap displaying the relative expression of marker genes across all clusters in 24m (left) and 3m (right) groups. Red box indicates a cluster-specific gene expression pattern, with color intensity representing normalized expression level.

(c) Box plots showing the frequency of each cell cluster (C01–C29) in 24m (red) and 3m (blue) groups. Red boxes highlight clusters (C04–C05, C07–C08) with significant frequency differences between groups.

Quantification data are presented as mean ± SD, and statistical significance for Fig. S12 was determined by unpaired Student’s t-test, with significance levels defined as *p* < 0.05 (*) and *p* < 0.01 (**) as indicated.


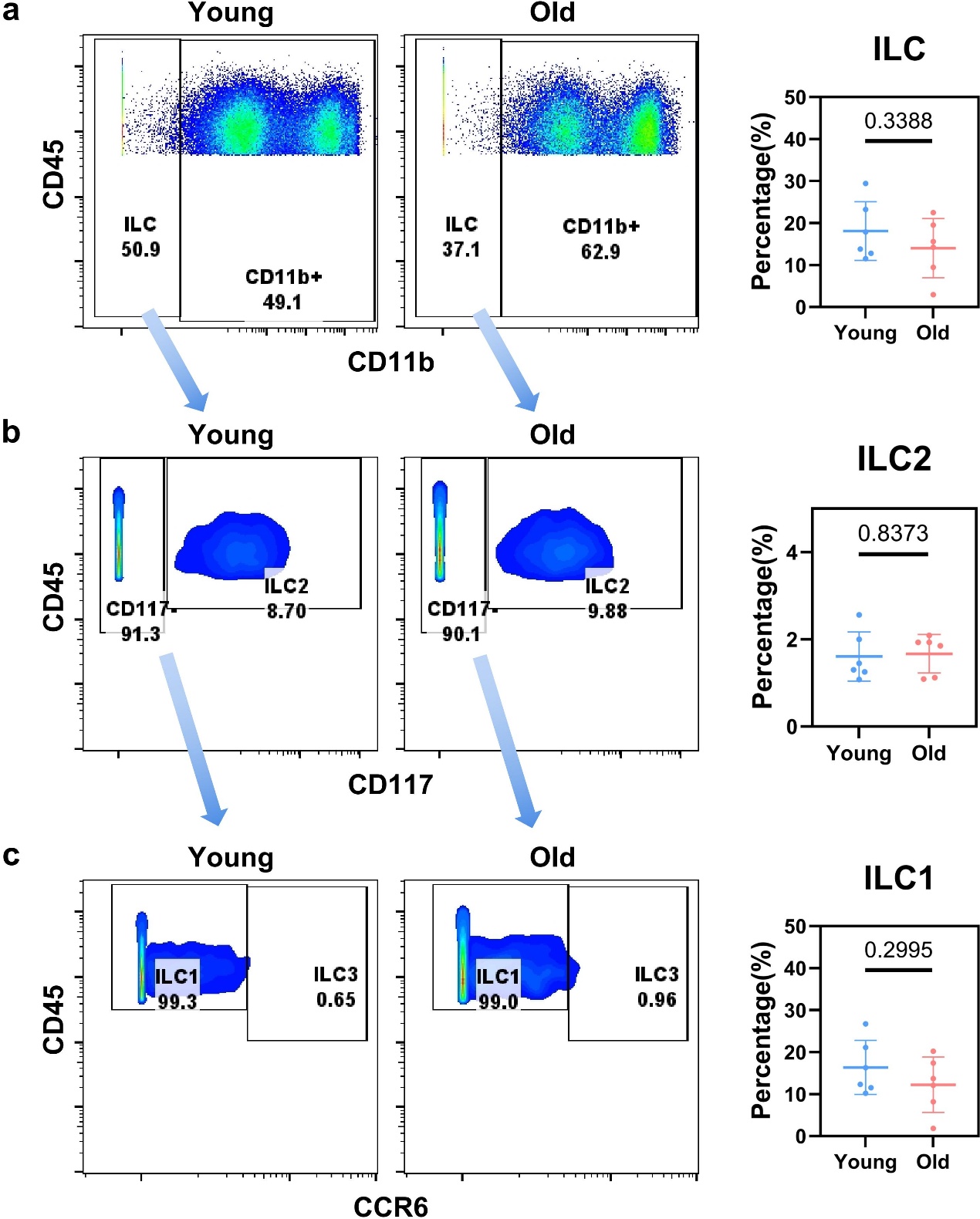


Fig. S13 Flow Cytometry Gating for Innate Lymphoid Cell Subsets in the Liver of Young and Aged Mice

(a) Flow cytometry plots (CD45 vs. CD11b gating) showing the distribution of Innate Lymphoid Cell (ILC) and CD11b⁺ populations in young and old mice, with quantification of ILC percentage.

(b) Flow cytometry plots (CD45 vs. CD117 gating) showing the distribution of CD117⁻ and ILC2 (CD117⁺) populations in young and old mice, with quantification of ILC2 percentage.

(c) Flow cytometry plots (CD45 vs. CCR6 gating) showing the distribution of ILC1 and ILC3 populations in young and old mice, with quantification of ILC1 percentage.


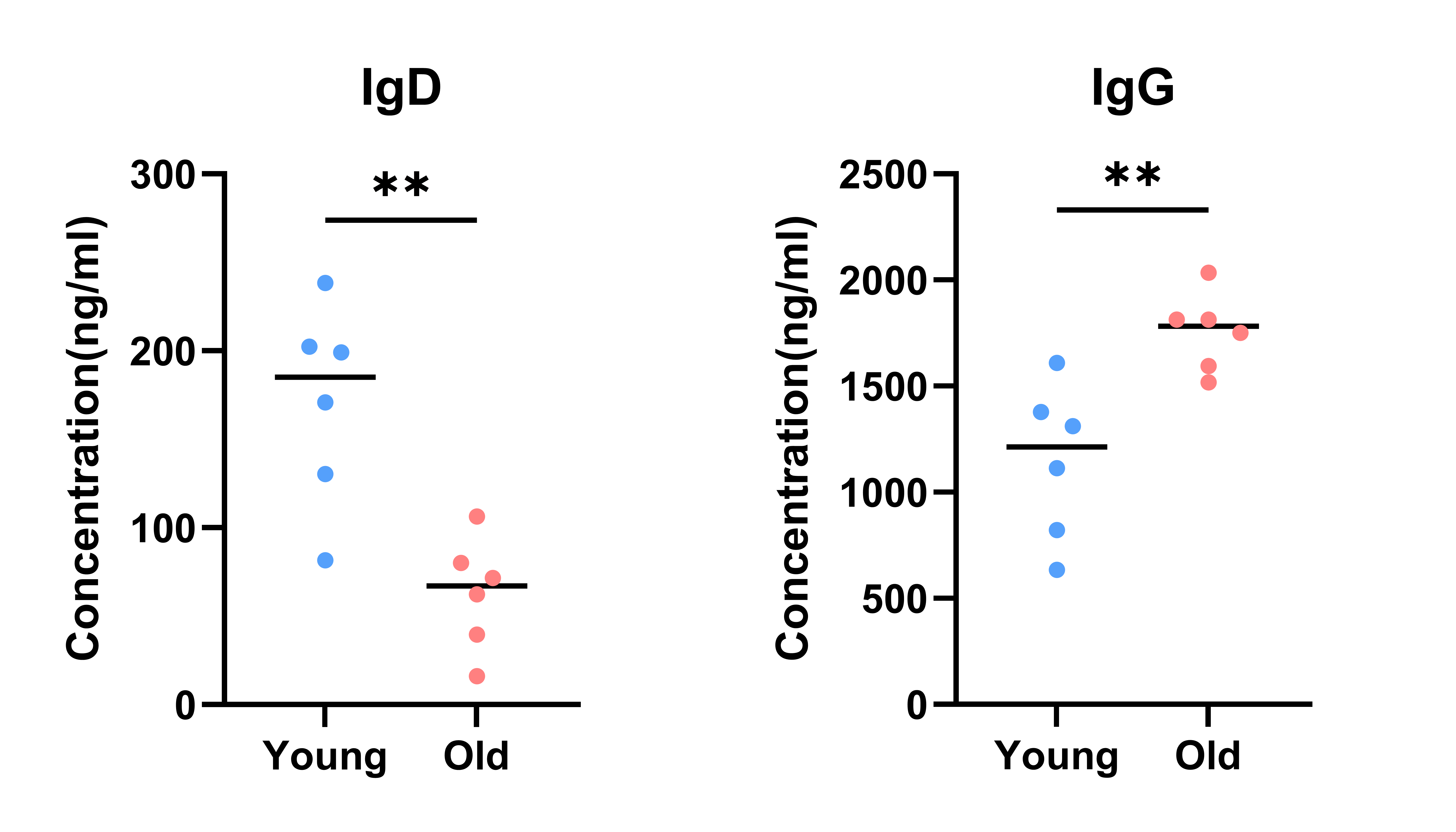


Fig. S14 ELISA detection of IgD and IgG levels in the livers of young and aged mice. Quantification data are presented as mean ± SD, and statistical significance for Fig. S14 was determined by unpaired Student’s t-test, with significance levels defined as *p* < 0.01 (**) as indicated.
