# Supplementary figures and images for: Single-cell CyTOF profiling reveals alterations in B, T and macrophage subsets during murine hepatic aging
Source: Front Immunol. 2026 Apr 28;17:1787641. doi: 10.3389/fimmu.2026.1787641 (PMC13160732; doi:10.3389/fimmu.2026.1787641)

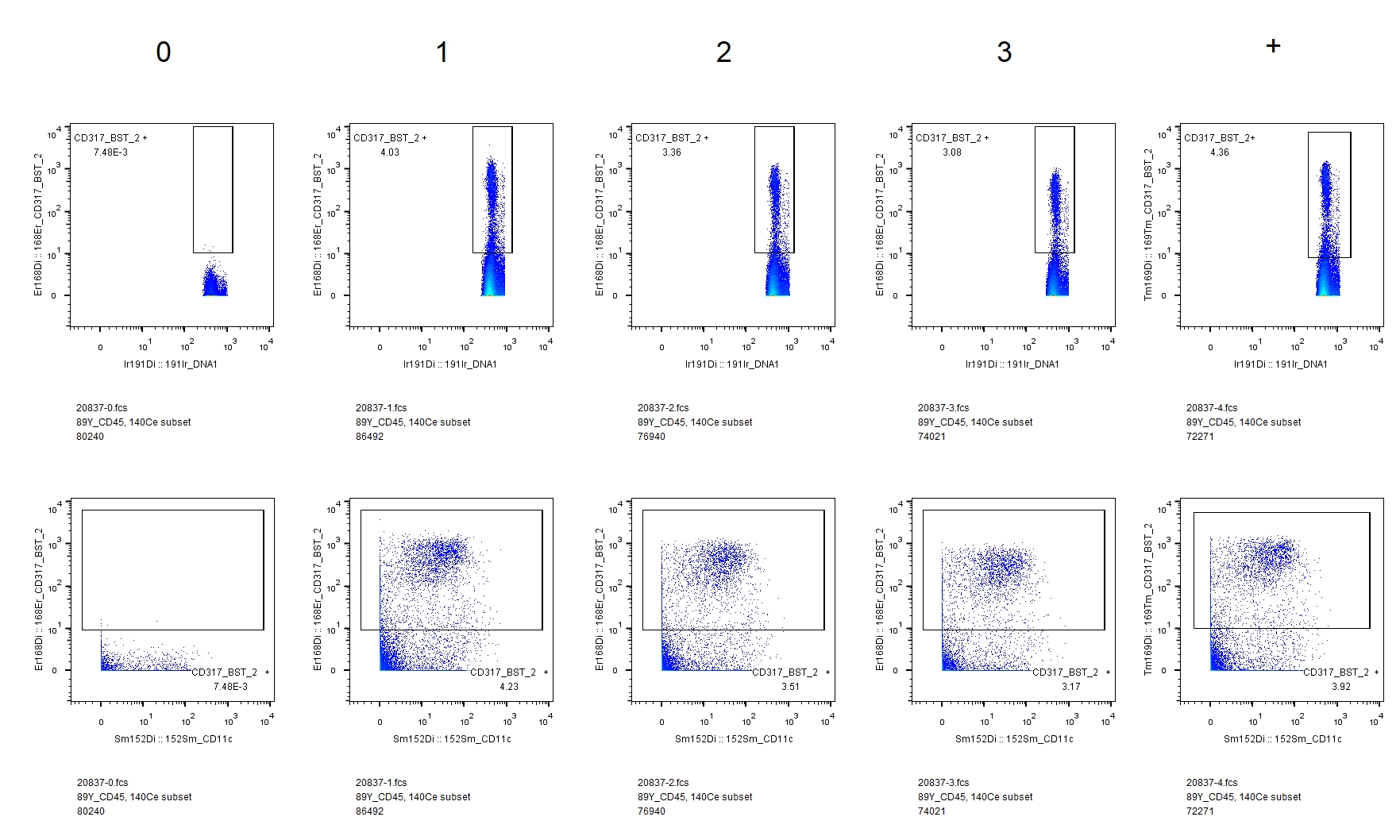

Supplement: Supplementary file 2 [file SupplementaryFile1.zip › 抗体测试结果图/020837-168-CD317-BST.jpg]

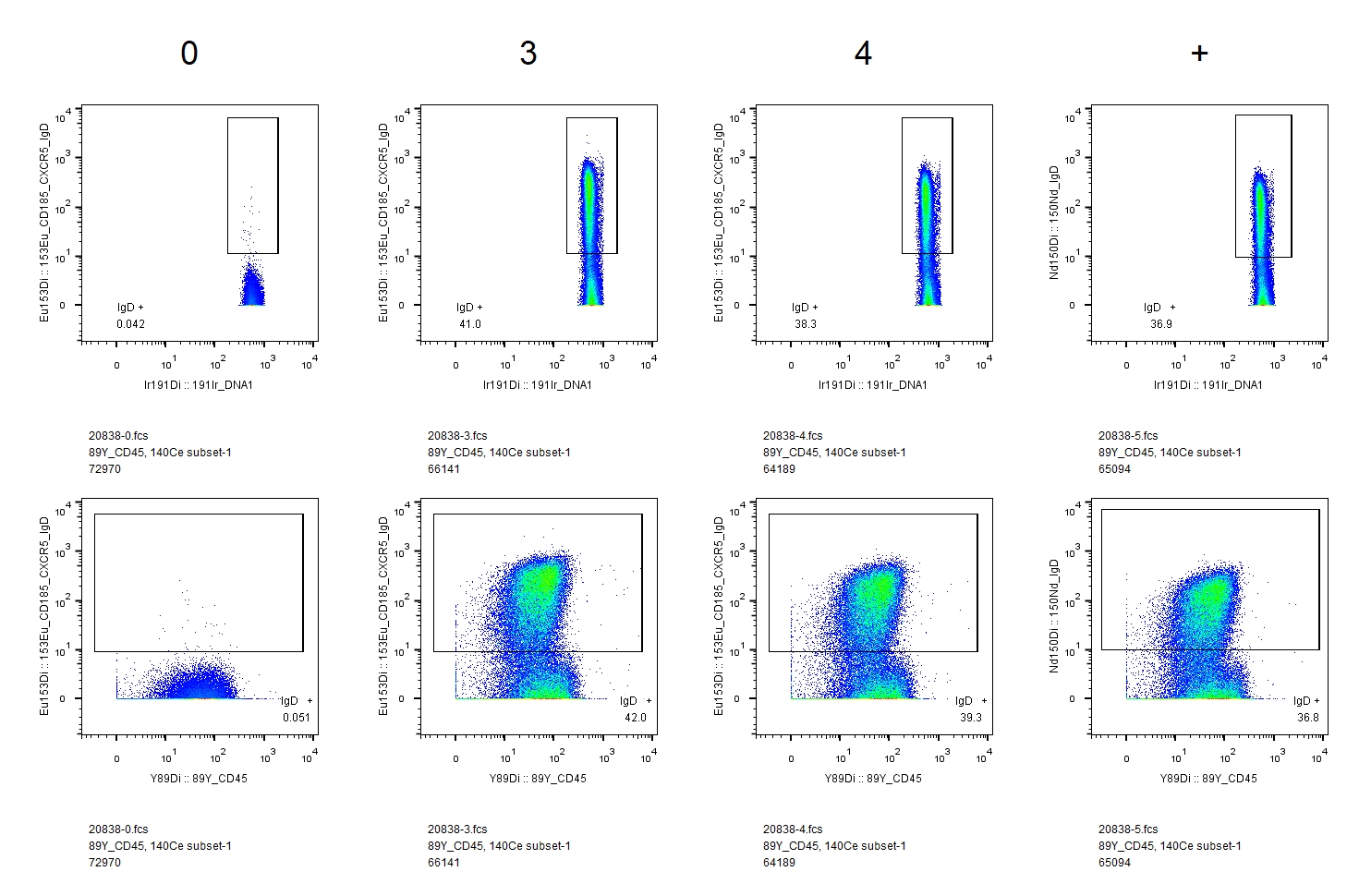

Supplement: Supplementary file 2 [file SupplementaryFile1.zip › 抗体测试结果图/020838-153-IgD.jpg]

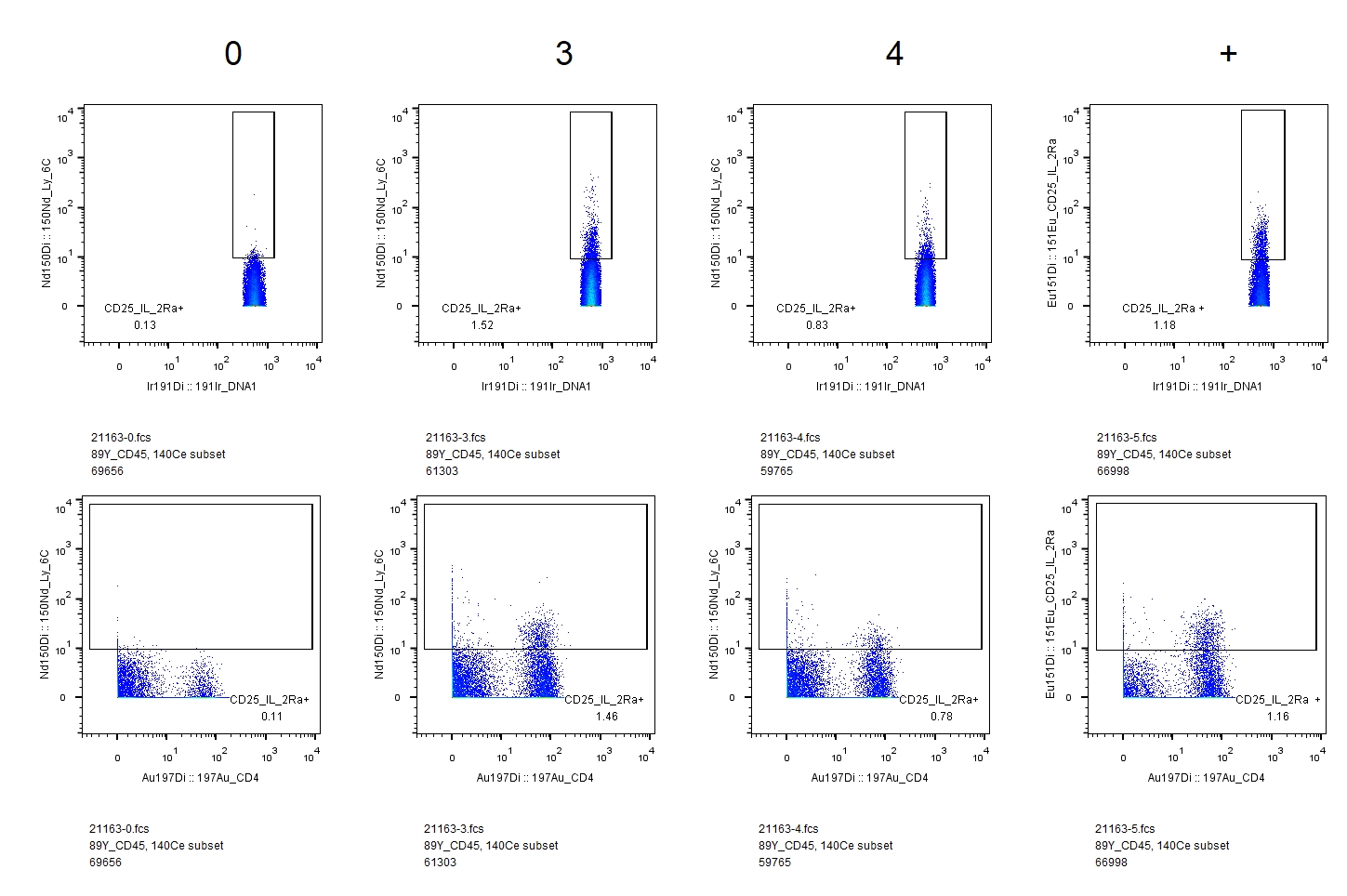

Supplement: Supplementary file 2 [file SupplementaryFile1.zip › 抗体测试结果图/021163-150-CD25-IL-2Ra.jpg]

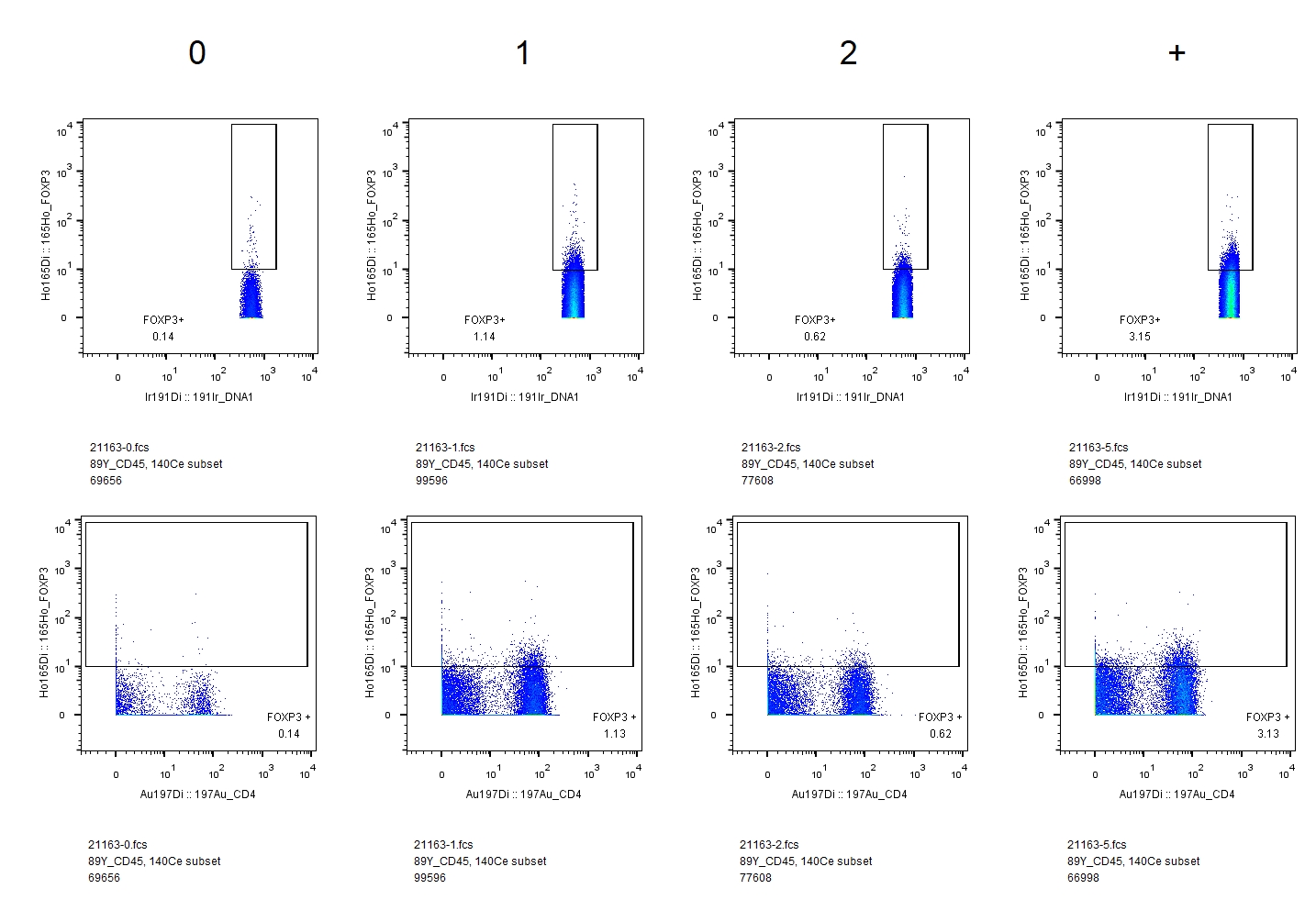

Supplement: Supplementary file 2 [file SupplementaryFile1.zip › 抗体测试结果图/021163-165-FOXP3.jpg]

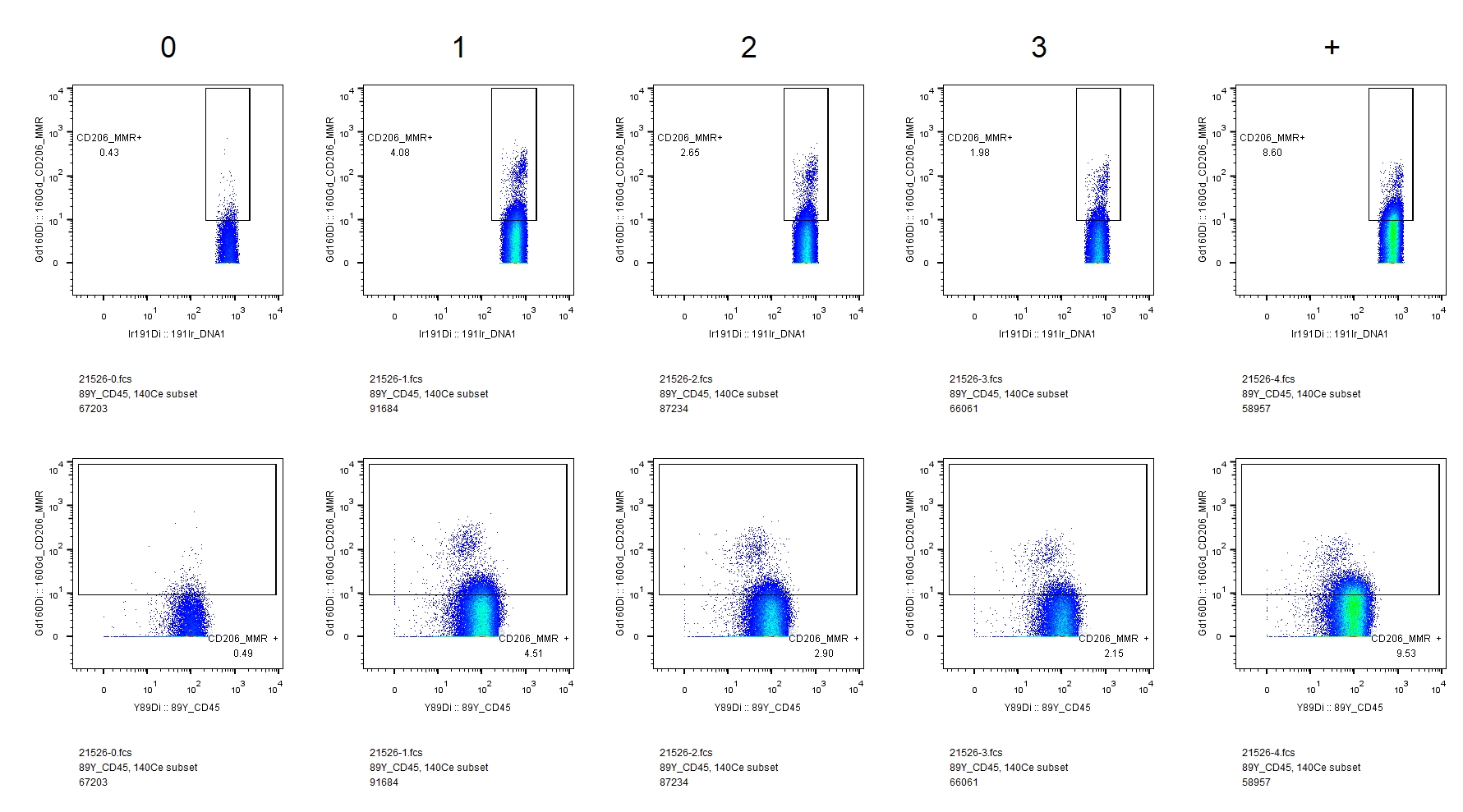

Supplement: Supplementary file 2 [file SupplementaryFile1.zip › 抗体测试结果图/021526-160-CD206-MMR.jpg]

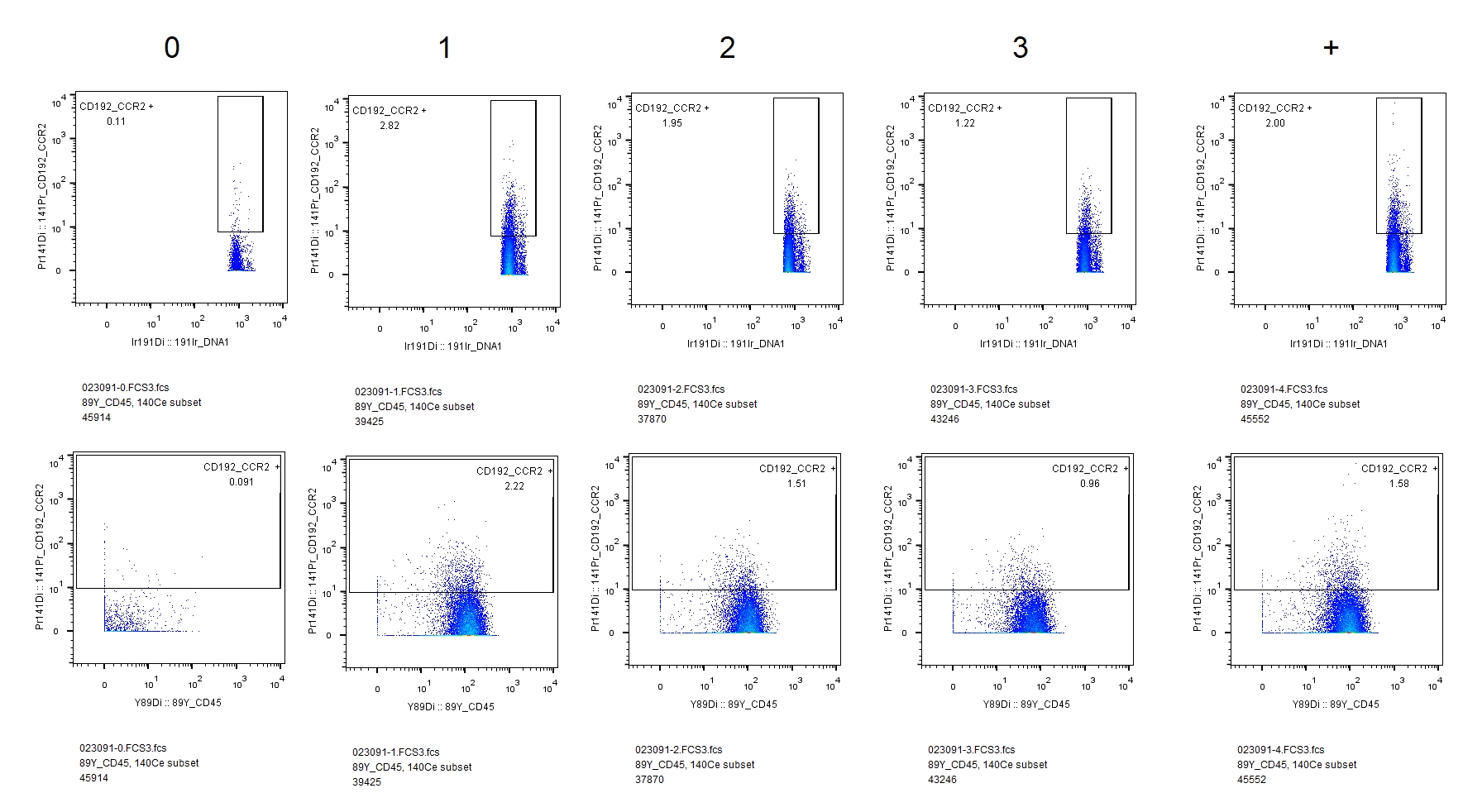

Supplement: Supplementary file 2 [file SupplementaryFile1.zip › 抗体测试结果图/023091-141Pr-CD192-CCR2.jpg]

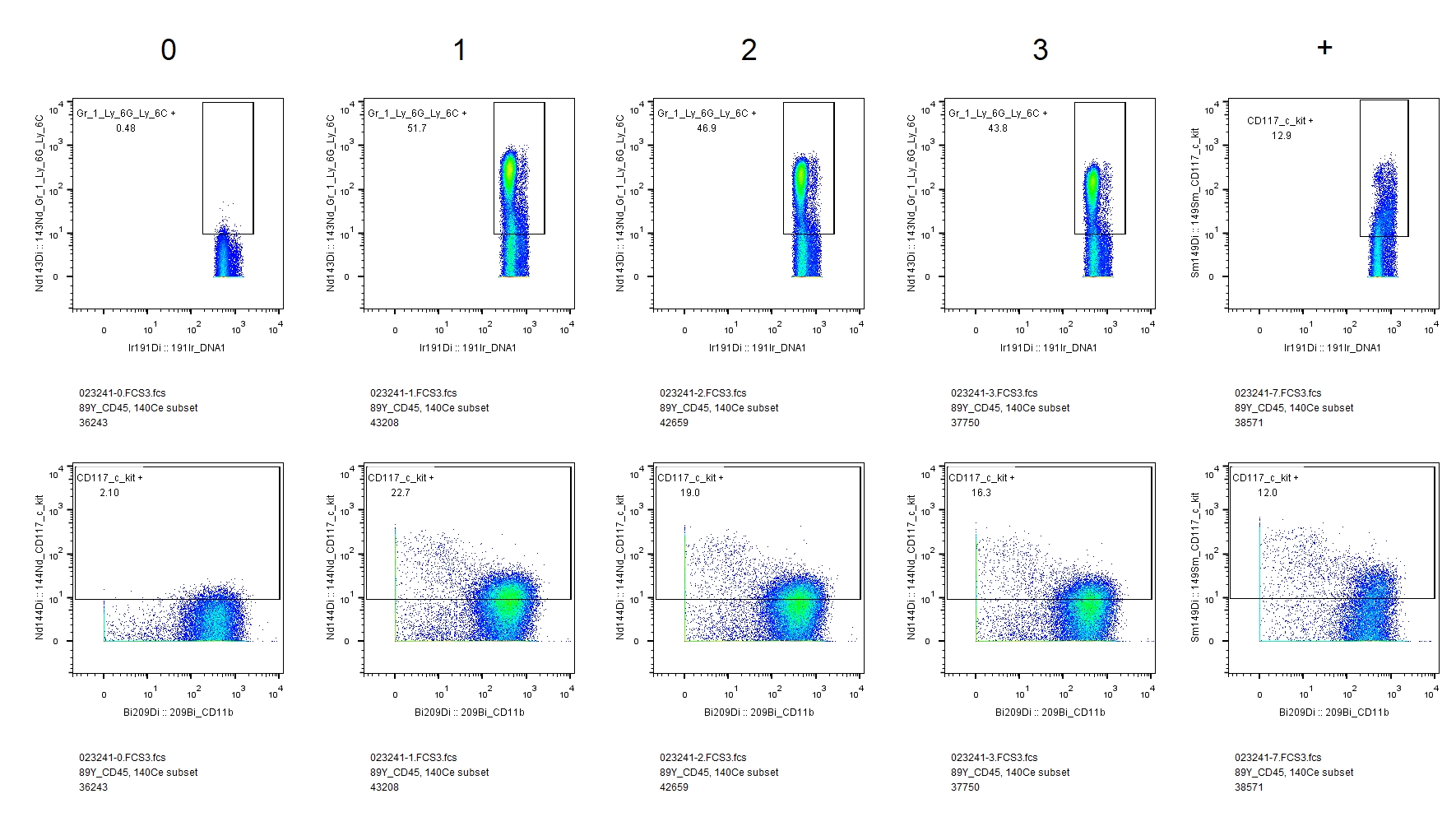

Supplement: Supplementary file 2 [file SupplementaryFile1.zip › 抗体测试结果图/023241-144Nd-CD117-c-kit.jpg]

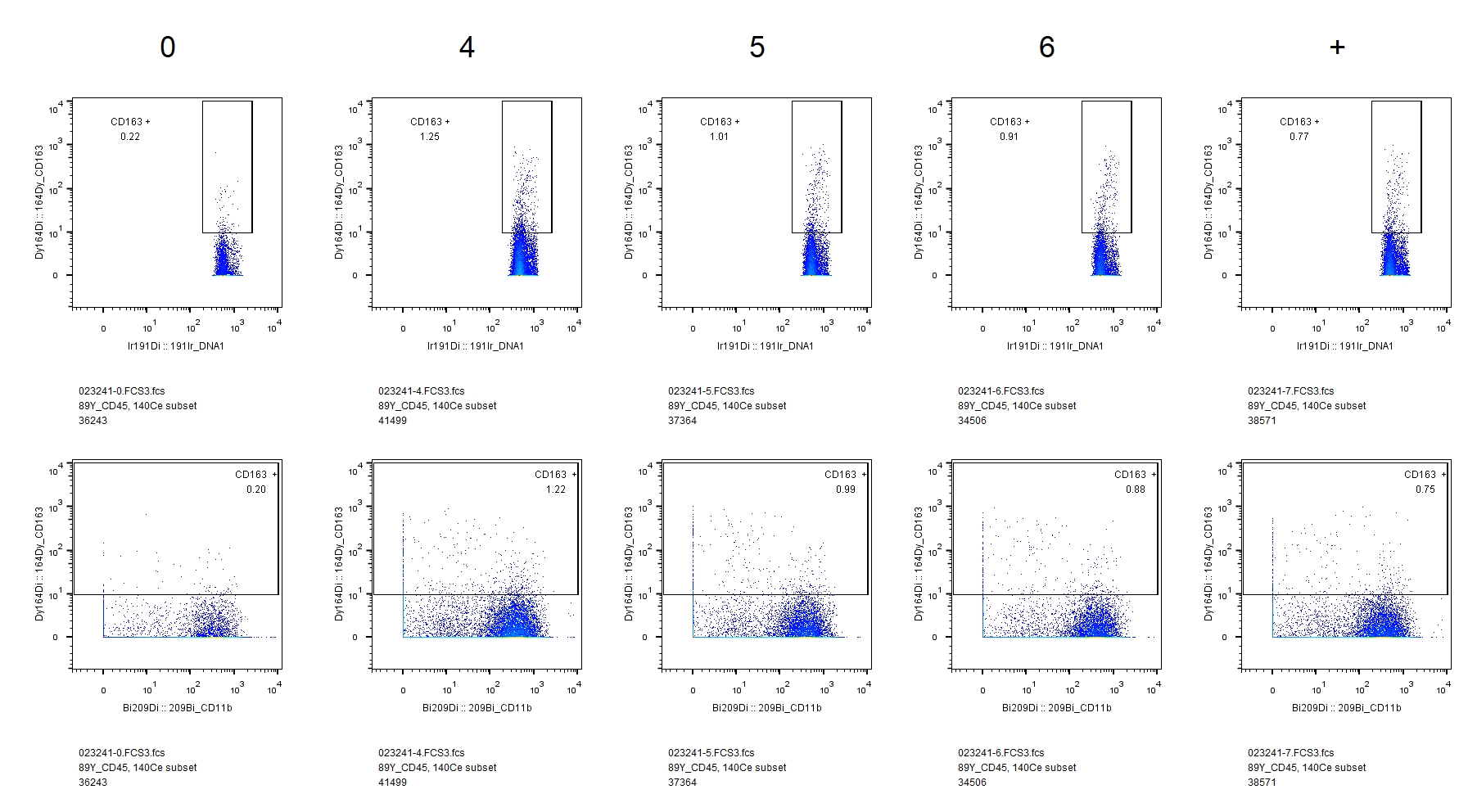

Supplement: Supplementary file 2 [file SupplementaryFile1.zip › 抗体测试结果图/023241-164Dy-CD163.jpg]

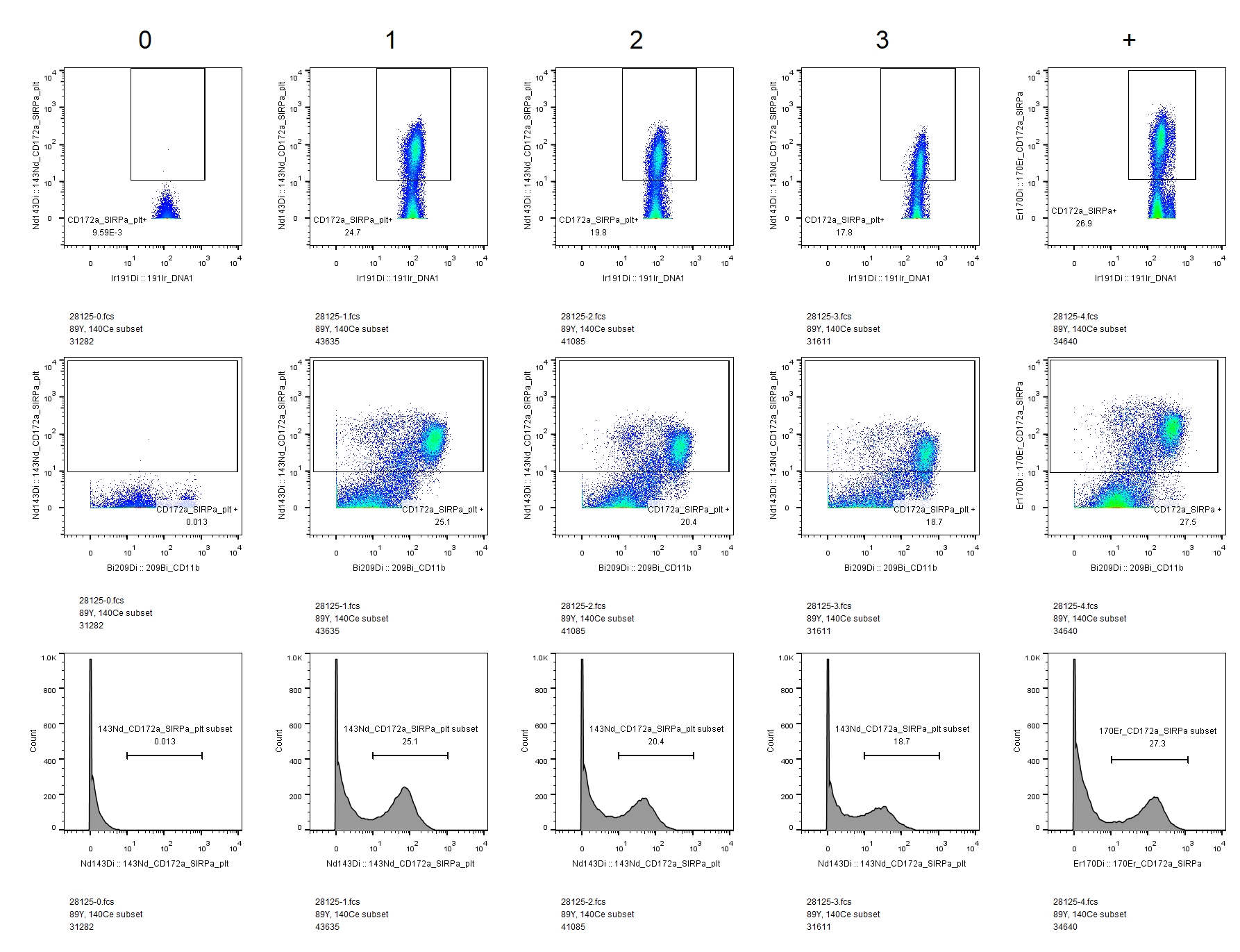

Supplement: Supplementary file 2 [file SupplementaryFile1.zip › 抗体测试结果图/028125-143-CD172a-SIRPa-plt.jpg]

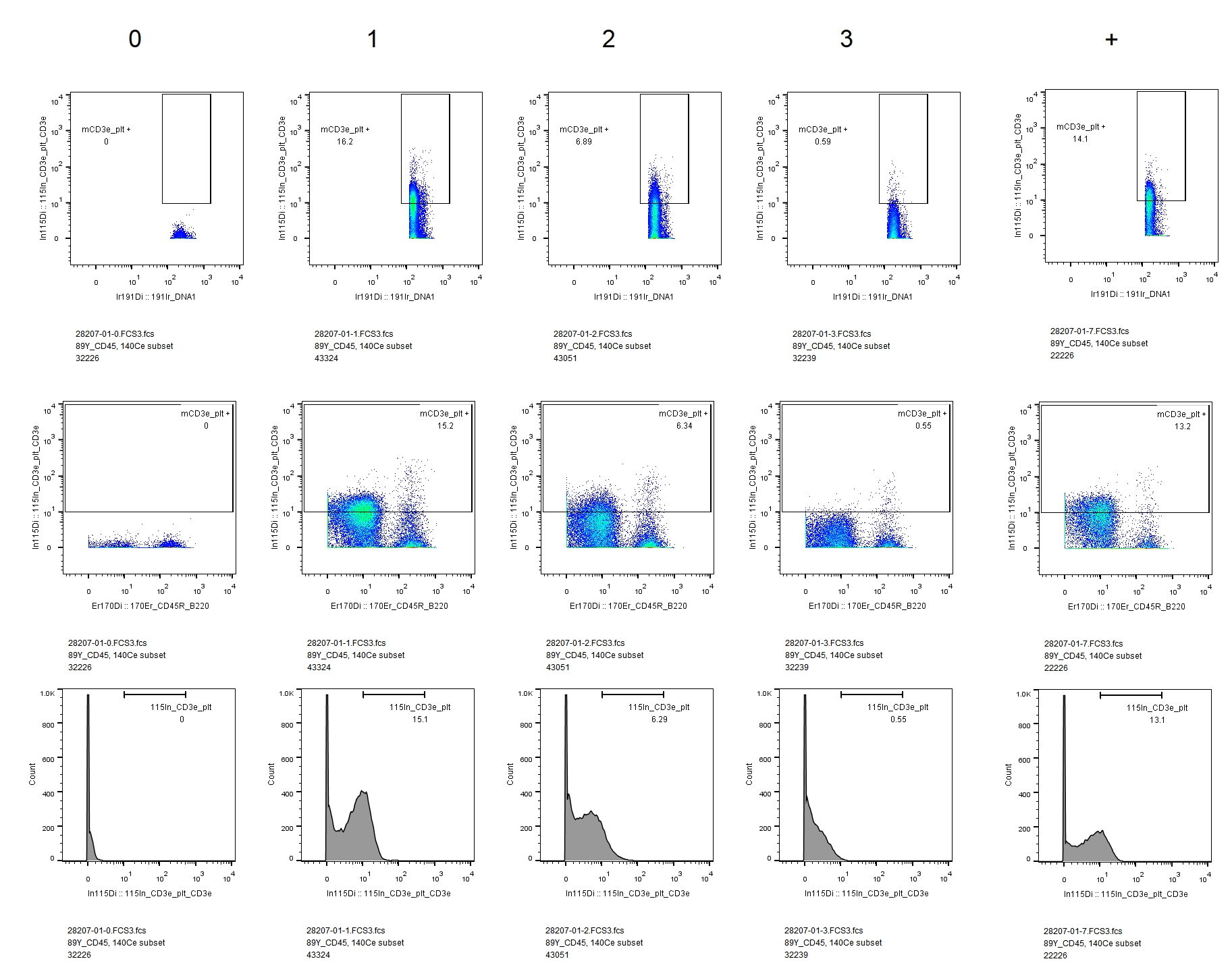

Supplement: Supplementary file 2 [file SupplementaryFile1.zip › 抗体测试结果图/028207-01-115-mCD3e-plt.jpg]

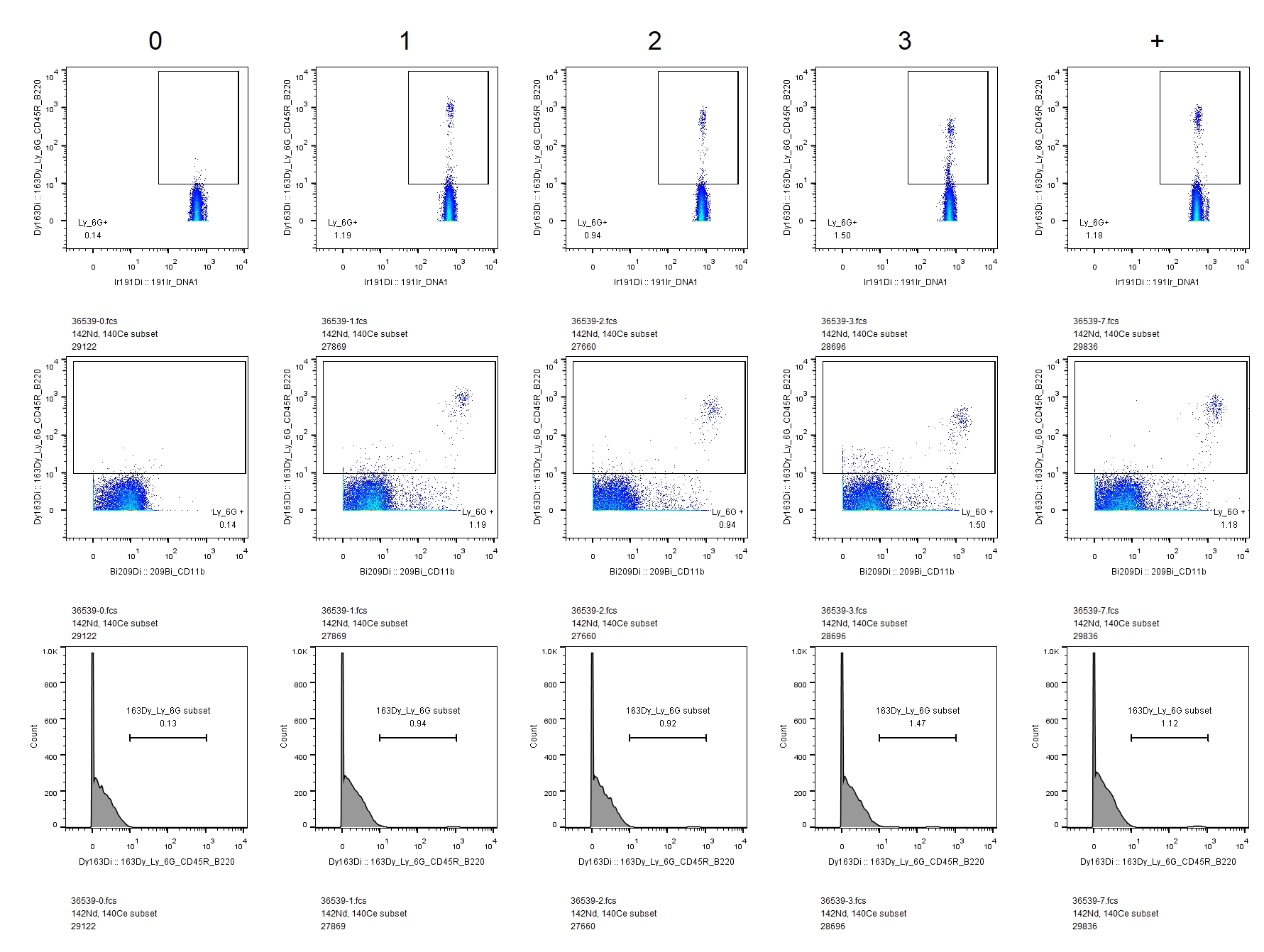

Supplement: Supplementary file 2 [file SupplementaryFile1.zip › 抗体测试结果图/036539-163-mLy-6G-plt.jpg]

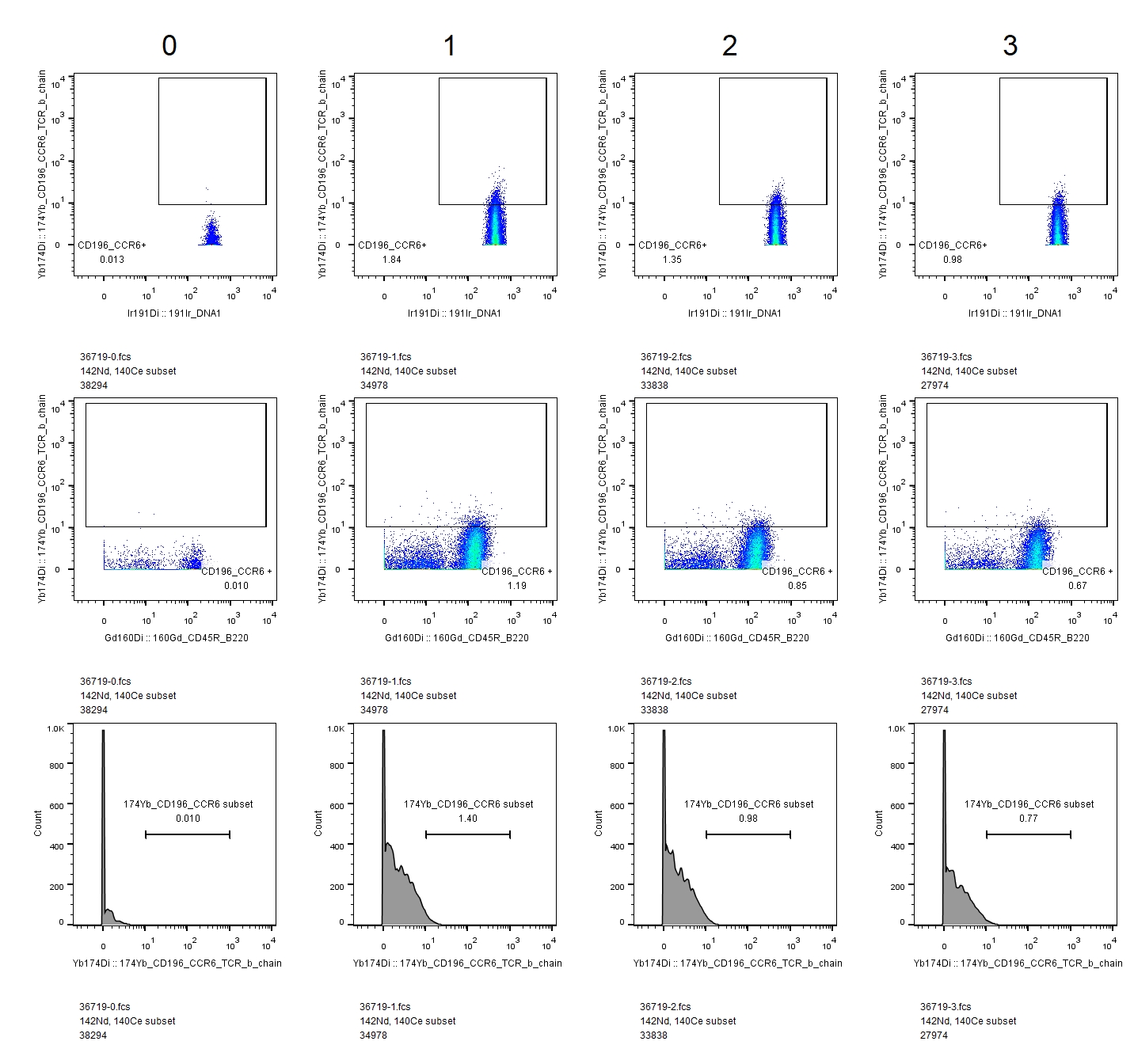

Supplement: Supplementary file 2 [file SupplementaryFile1.zip › 抗体测试结果图/036719-123-174-mCD196-CCR6-plt.jpg]

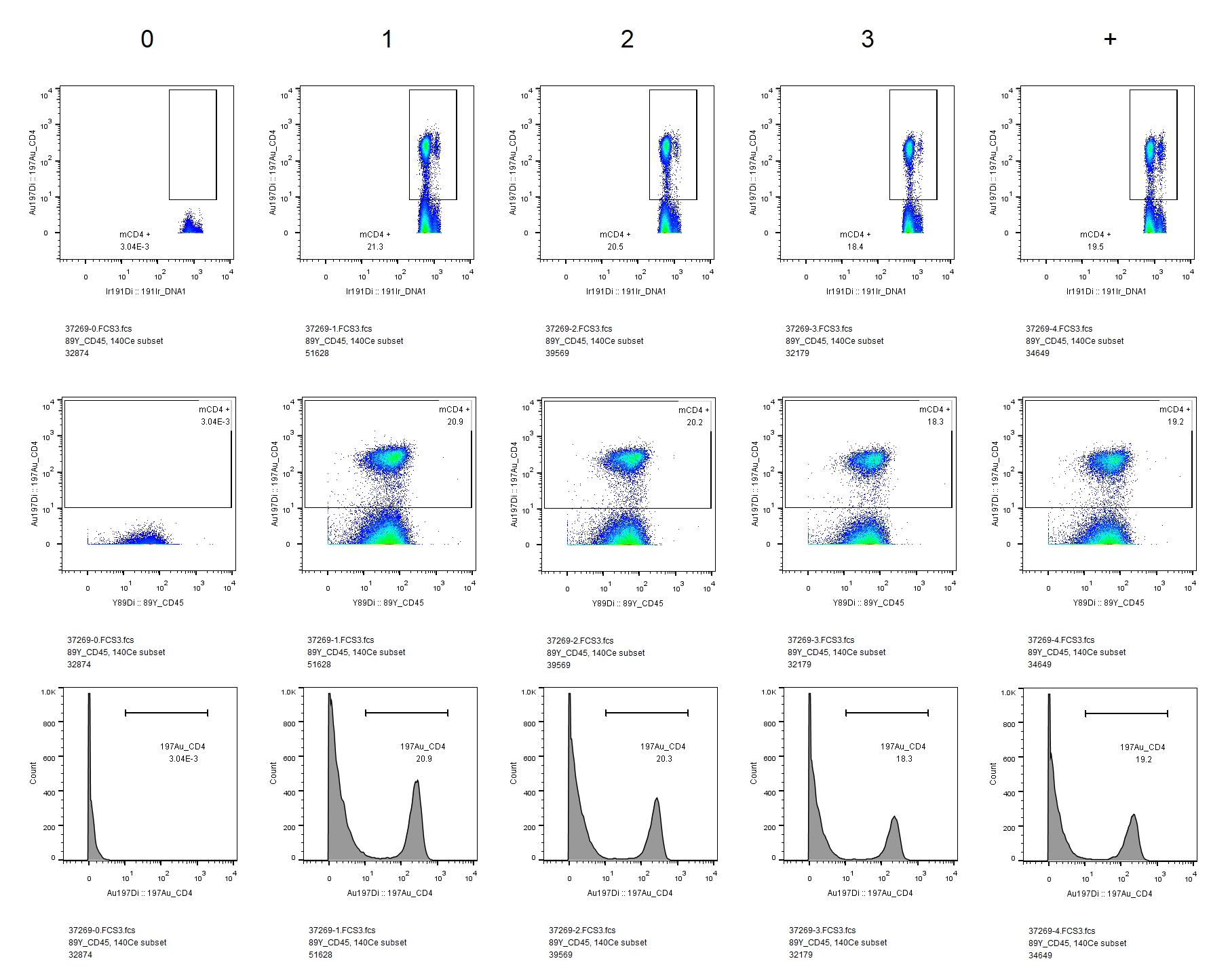

Supplement: Supplementary file 2 [file SupplementaryFile1.zip › 抗体测试结果图/037269-197-mCD4.jpg]

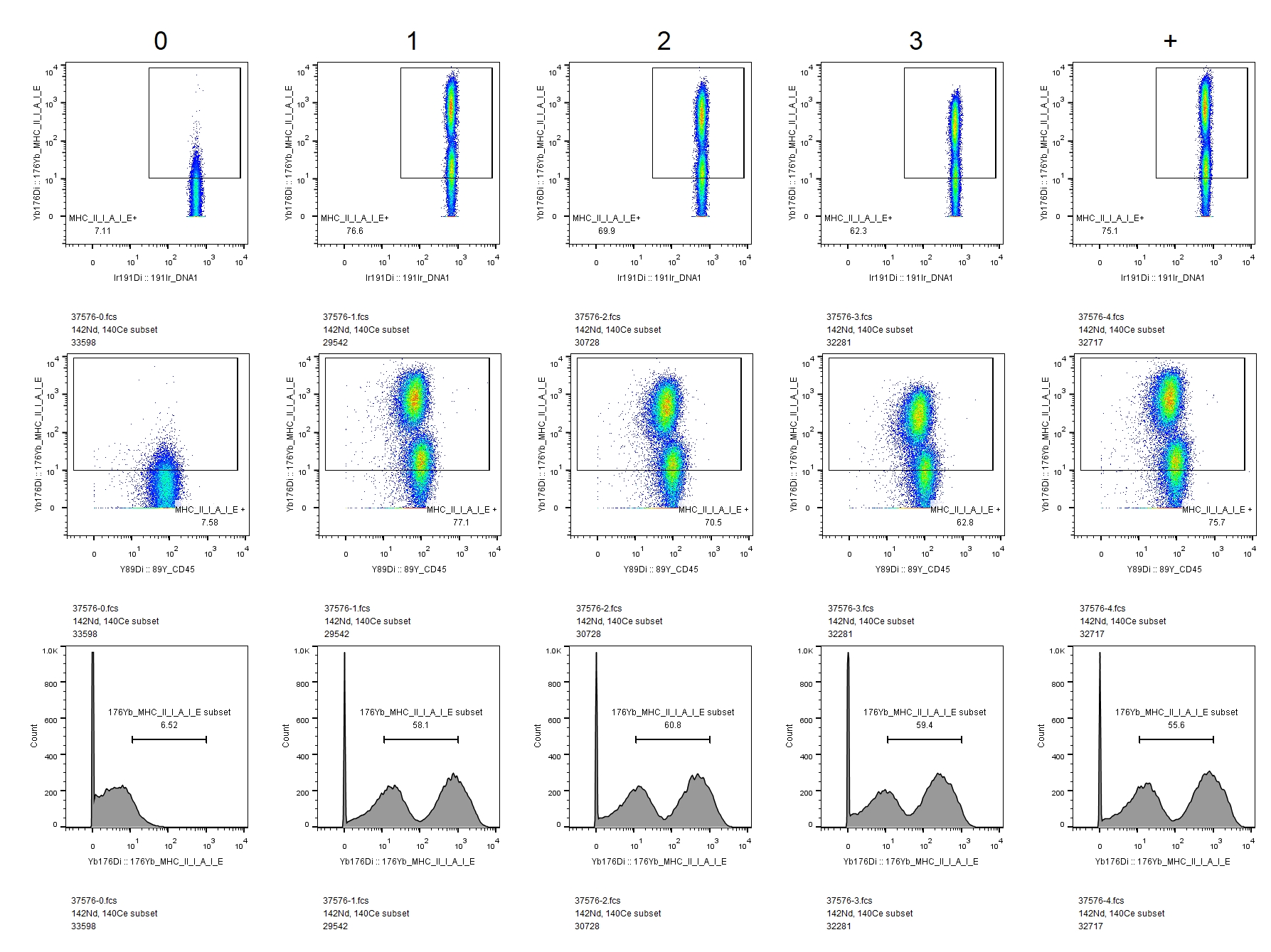

Supplement: Supplementary file 2 [file SupplementaryFile1.zip › 抗体测试结果图/037576-176-mMHC-plt.jpg]

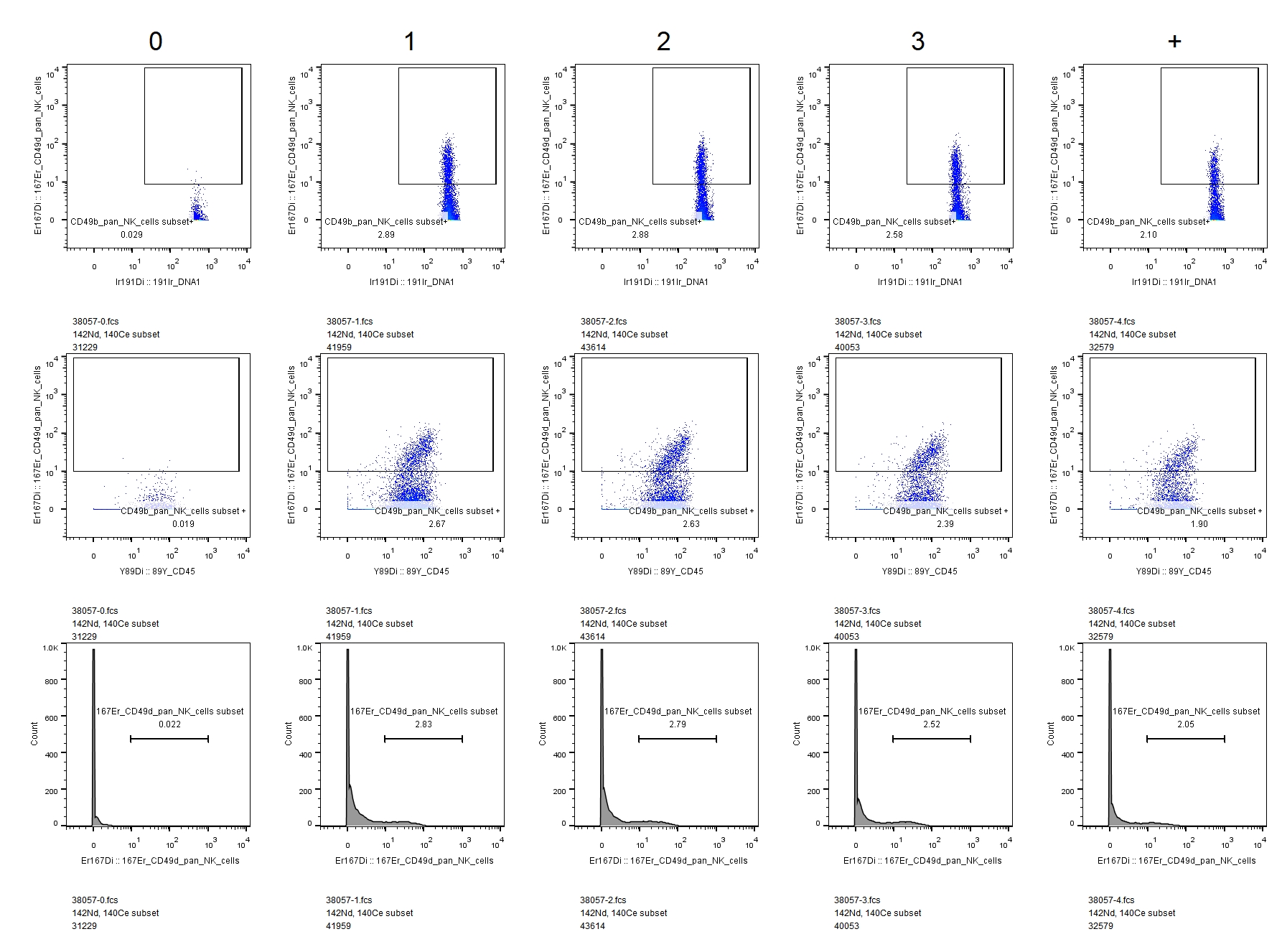

Supplement: Supplementary file 2 [file SupplementaryFile1.zip › 抗体测试结果图/038057-167-mCD49b-plt.jpg]

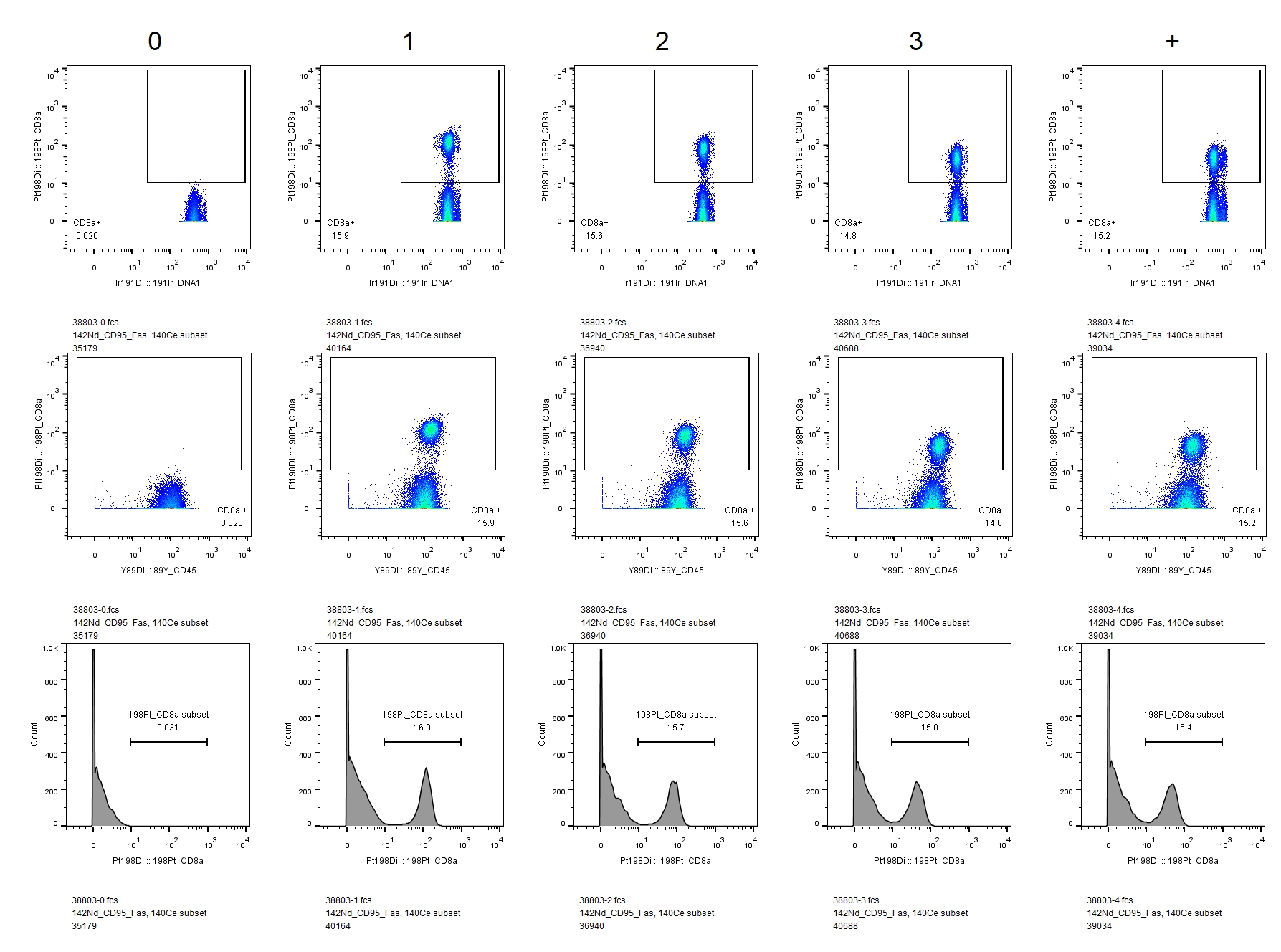

Supplement: Supplementary file 2 [file SupplementaryFile1.zip › 抗体测试结果图/038803-198-mCD8a.jpg]

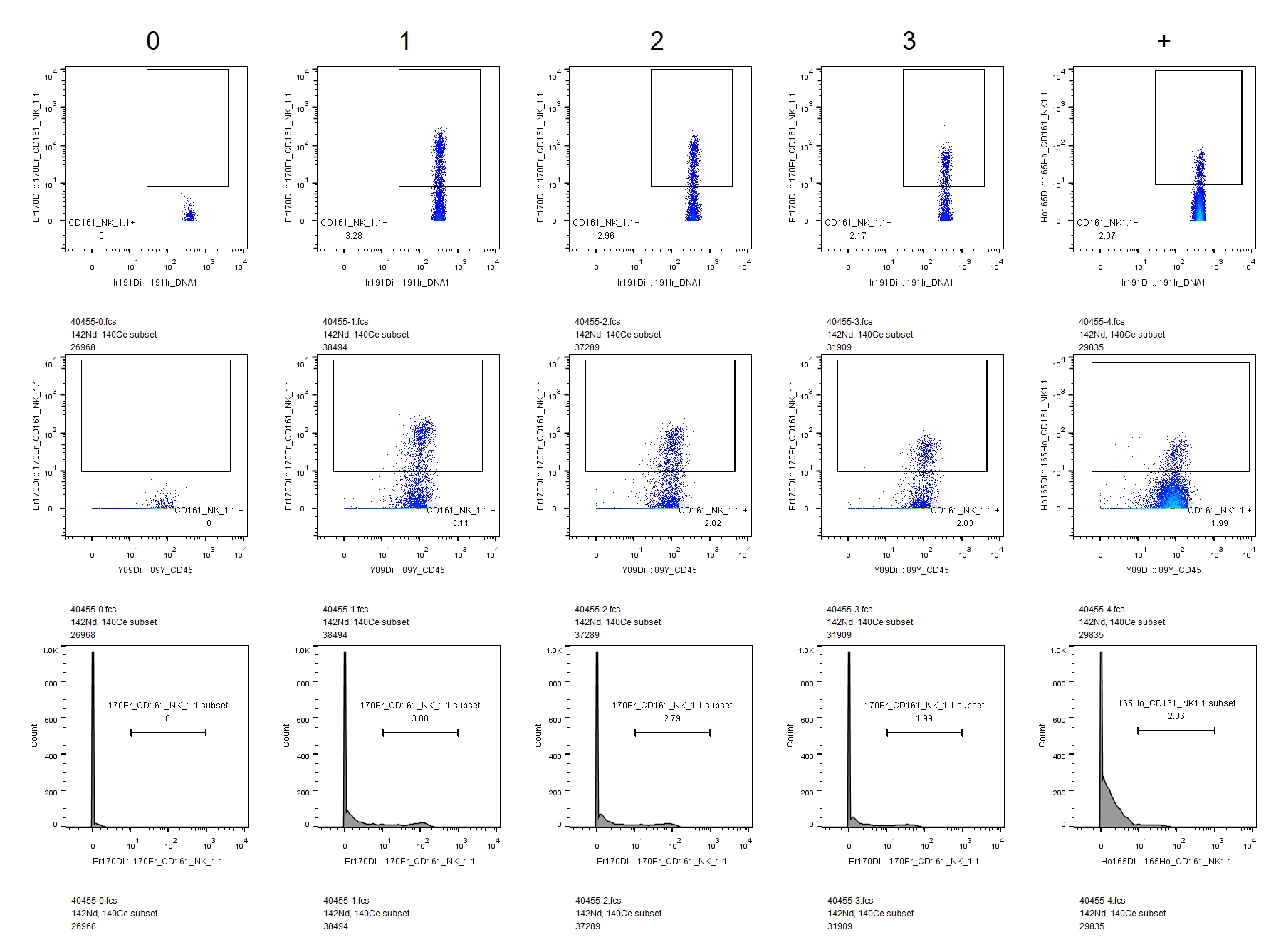

Supplement: Supplementary file 2 [file SupplementaryFile1.zip › 抗体测试结果图/040455-170-mCD161-NK-1.1-plt.jpg]

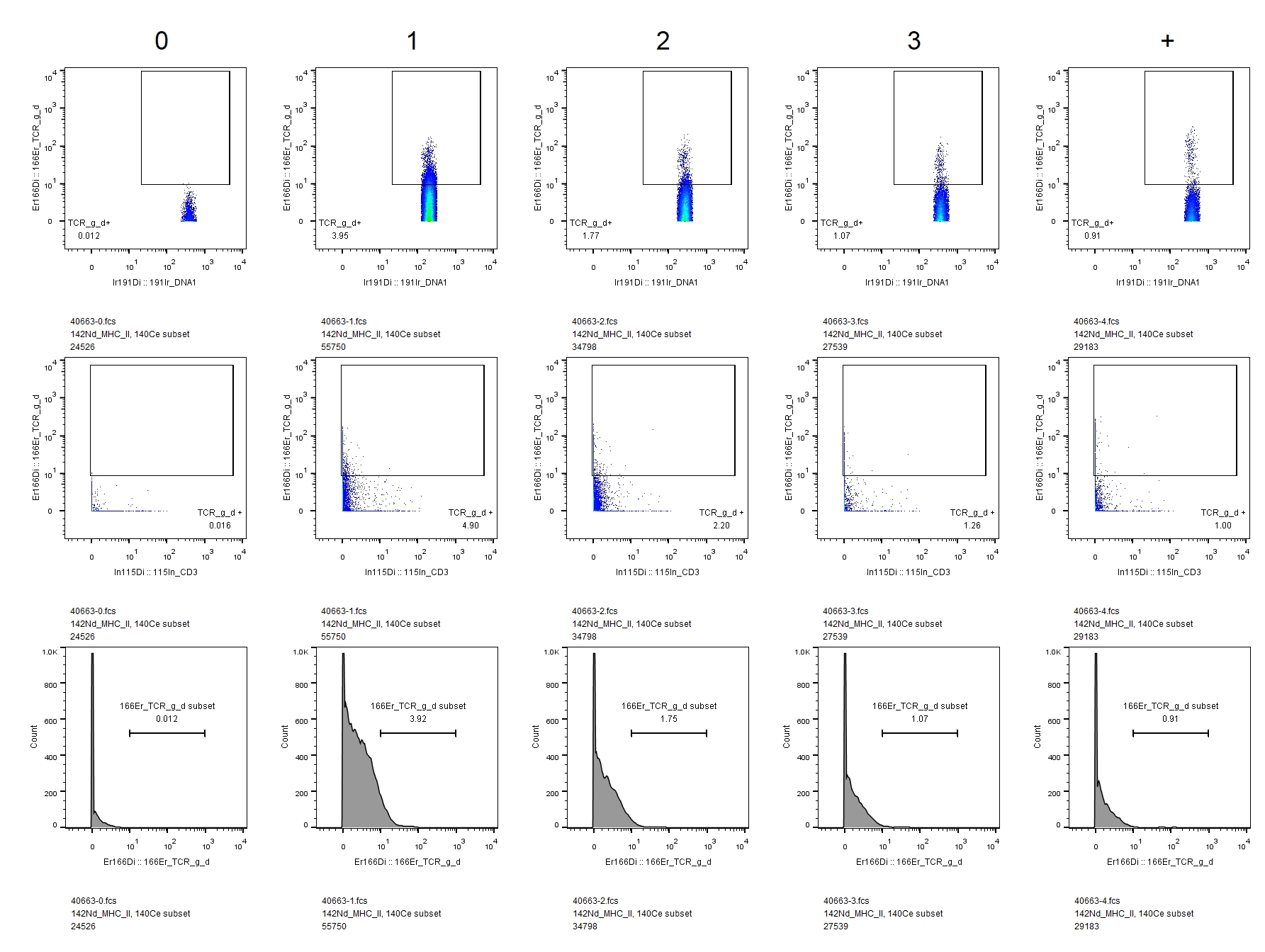

Supplement: Supplementary file 2 [file SupplementaryFile1.zip › 抗体测试结果图/040663-166-mTCRgd-plt.jpg]

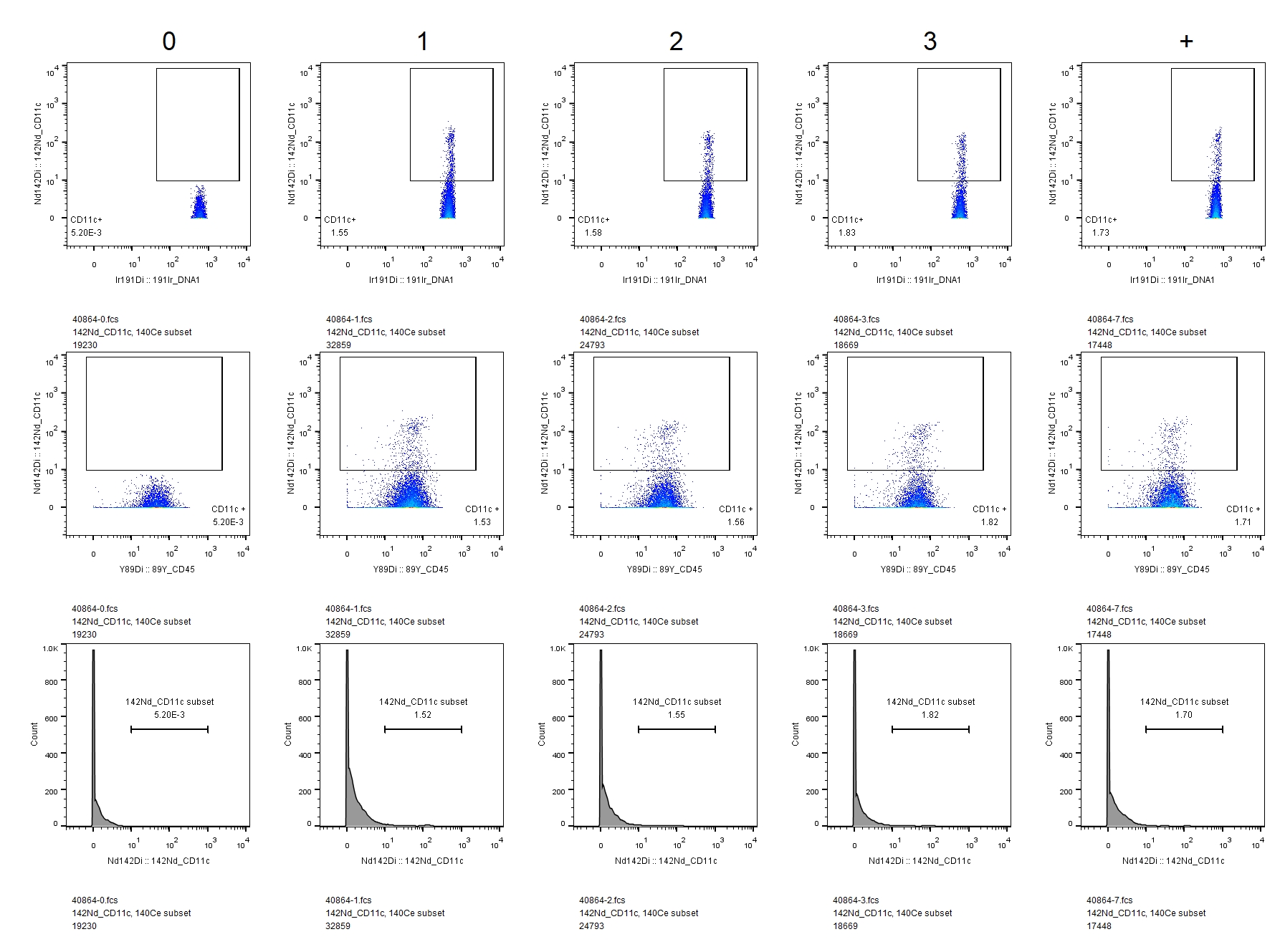

Supplement: Supplementary file 2 [file SupplementaryFile1.zip › 抗体测试结果图/040864-142-mCD11c-plt.jpg]

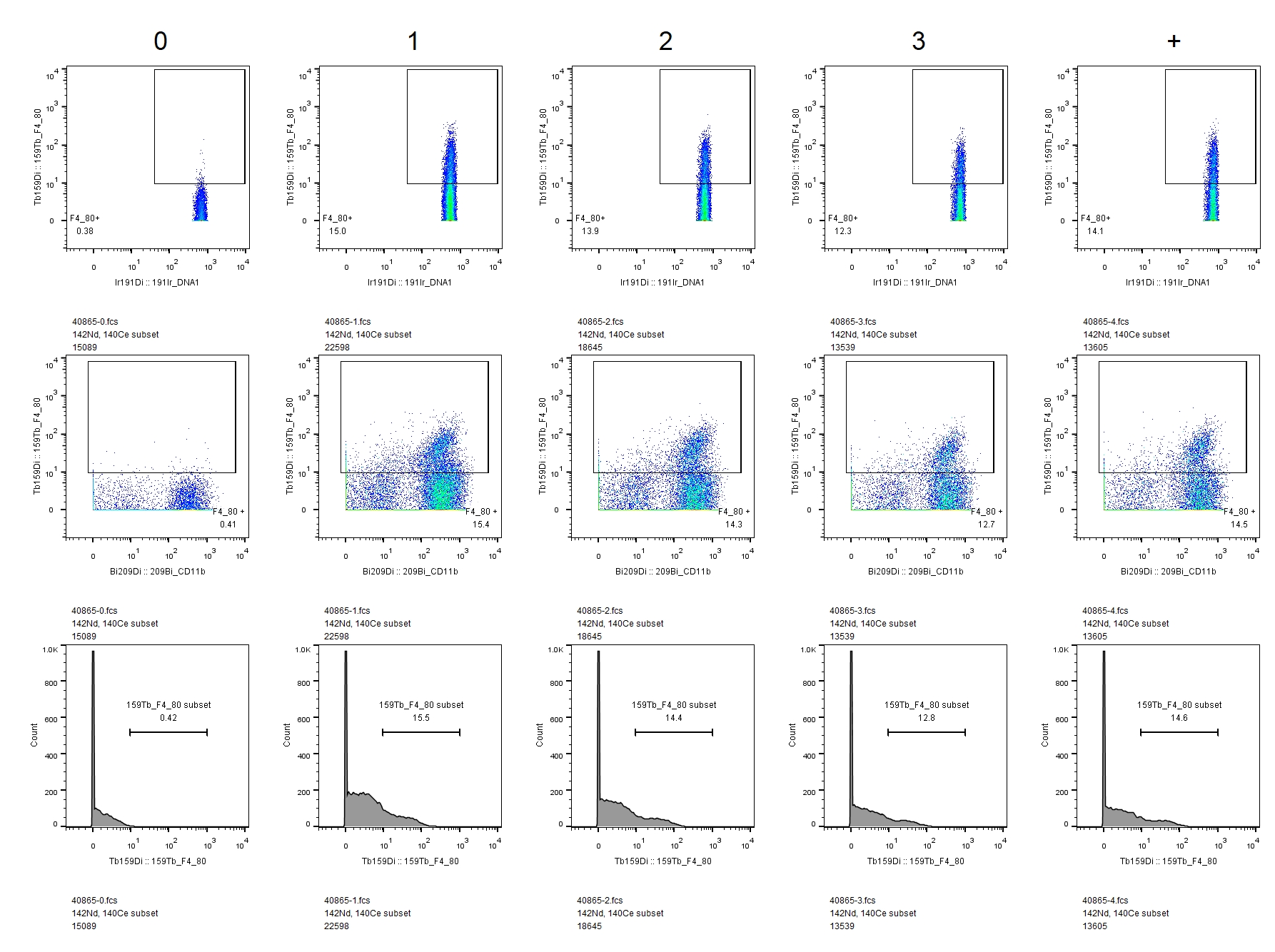

Supplement: Supplementary file 2 [file SupplementaryFile1.zip › 抗体测试结果图/040865-159-mF4-80-plt.jpg]

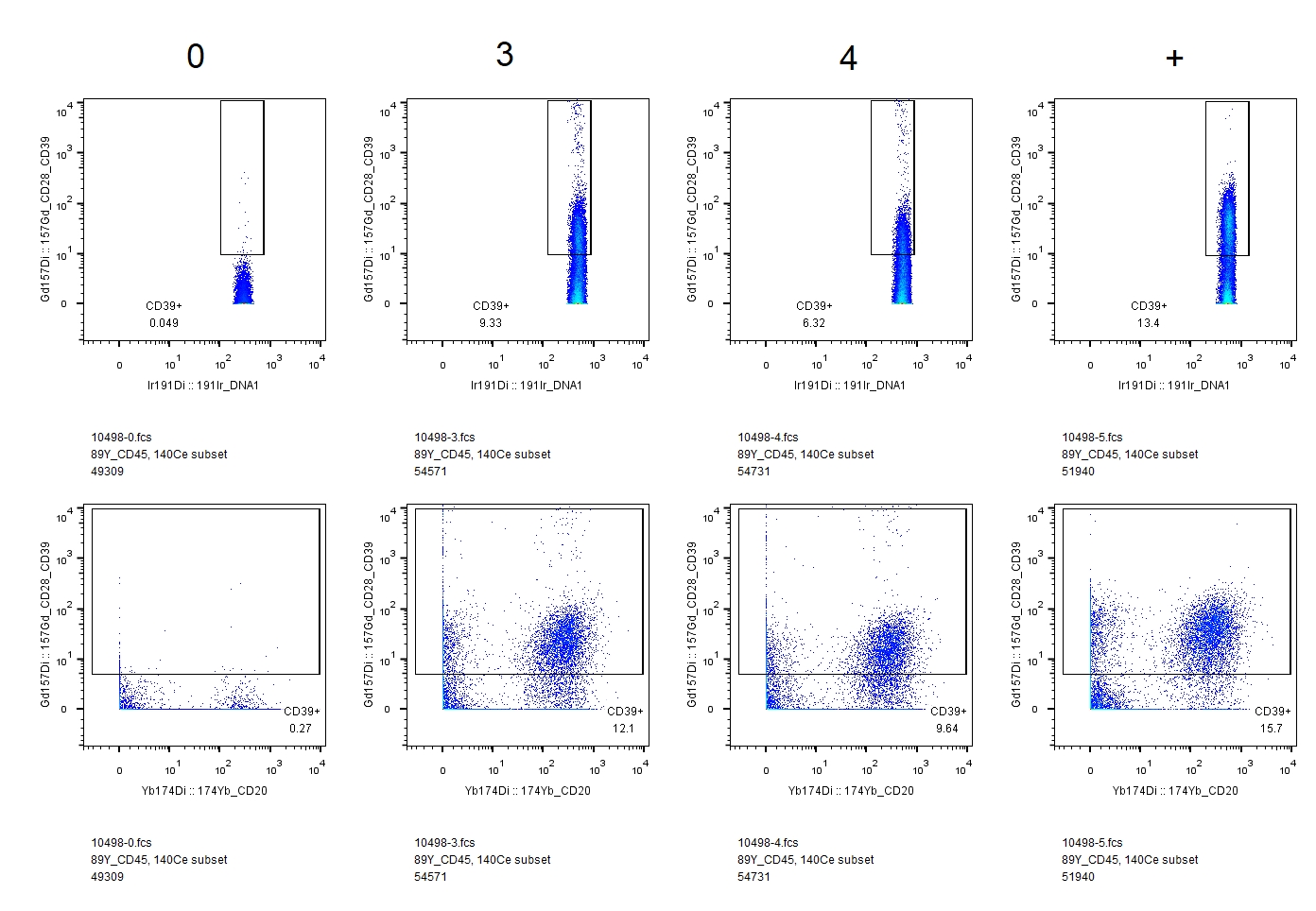

Supplement: Supplementary file 2 [file SupplementaryFile1.zip › 抗体测试结果图/10498-157-CD39.jpg]

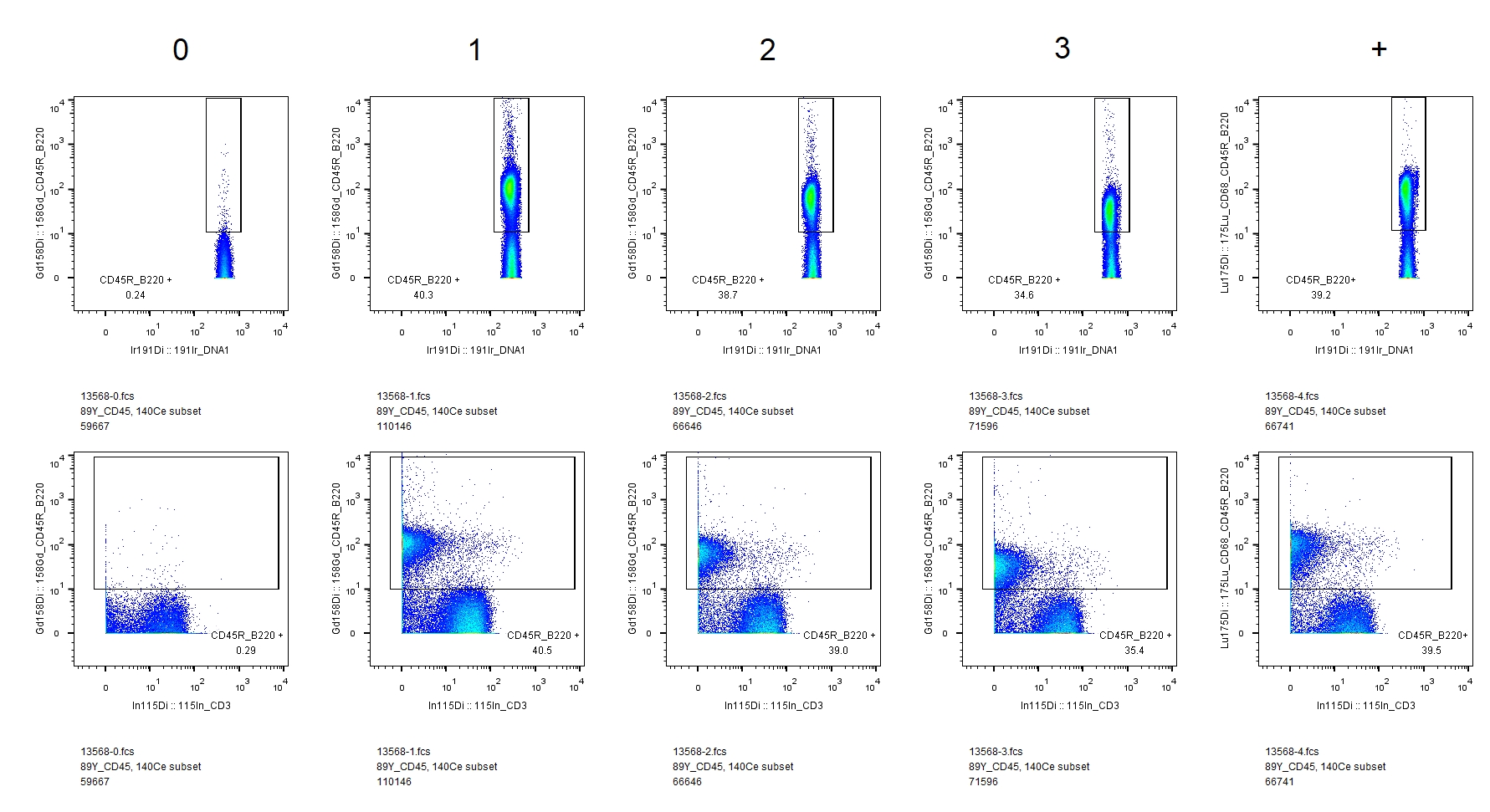

Supplement: Supplementary file 2 [file SupplementaryFile1.zip › 抗体测试结果图/13568-158-CD45R-B220.jpg]

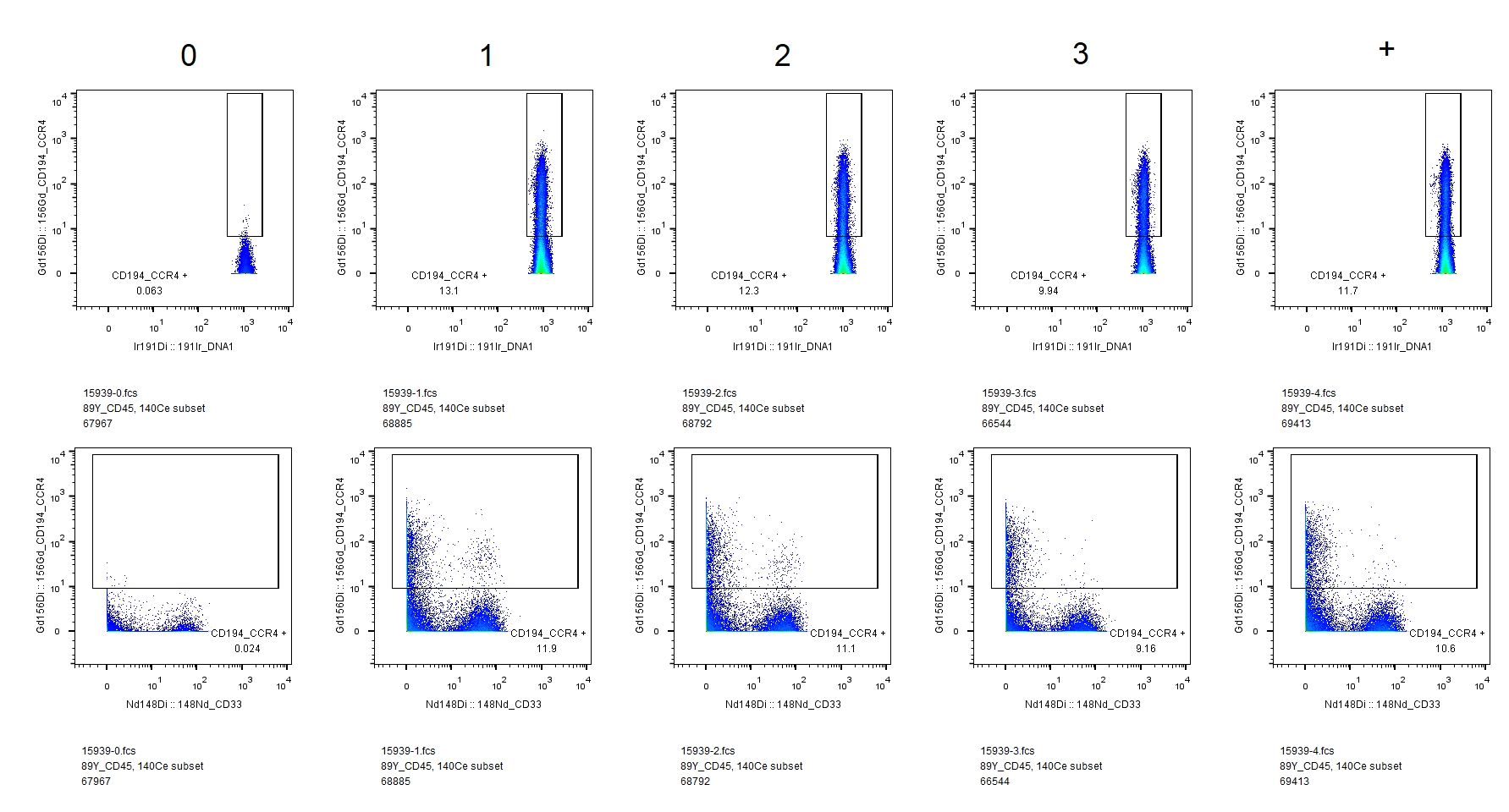

Supplement: Supplementary file 2 [file SupplementaryFile1.zip › 抗体测试结果图/15939-156-CD194-CCR4.jpg]

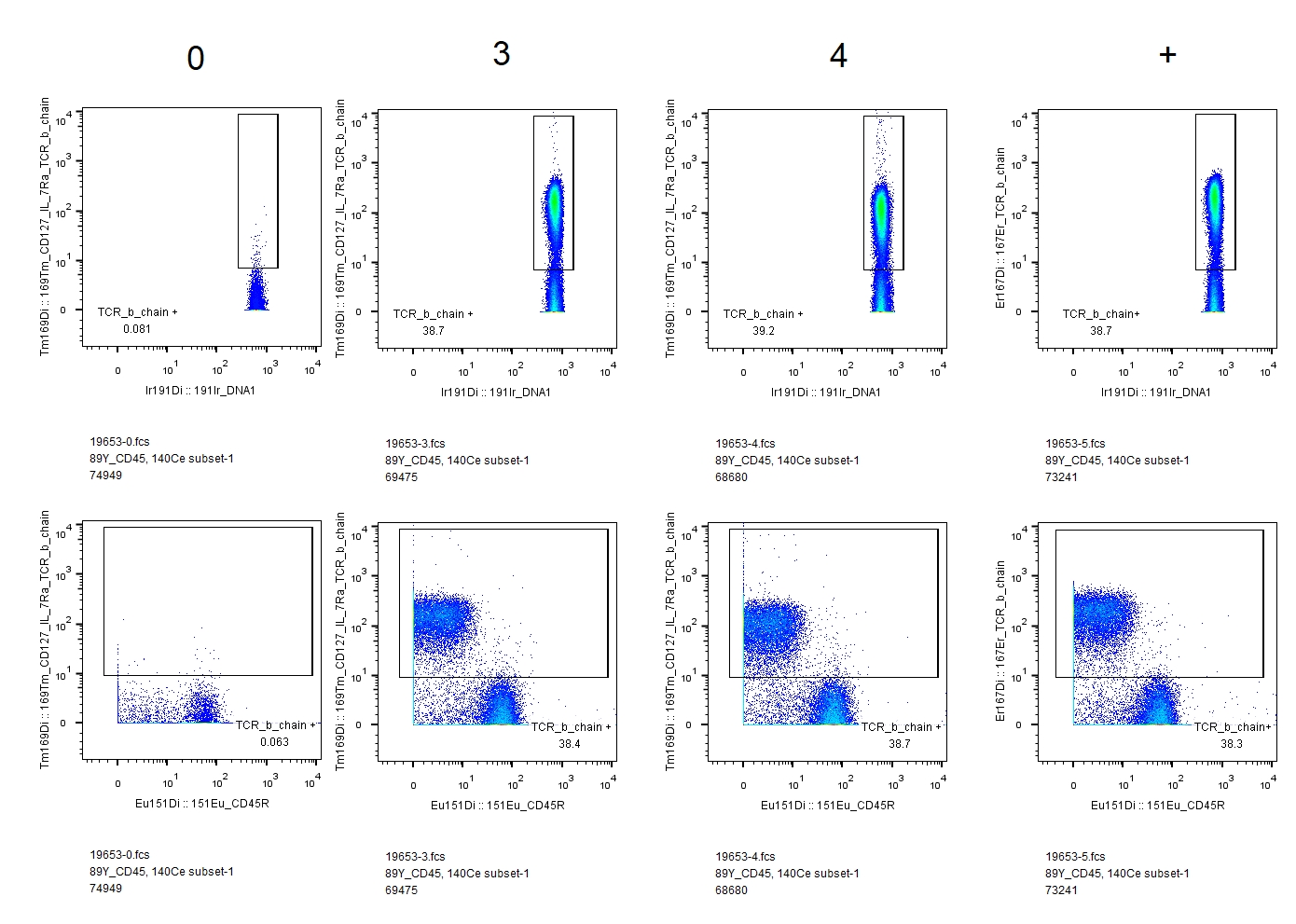

Supplement: Supplementary file 2 [file SupplementaryFile1.zip › 抗体测试结果图/19653-169-mTCR-b-chain.jpg]

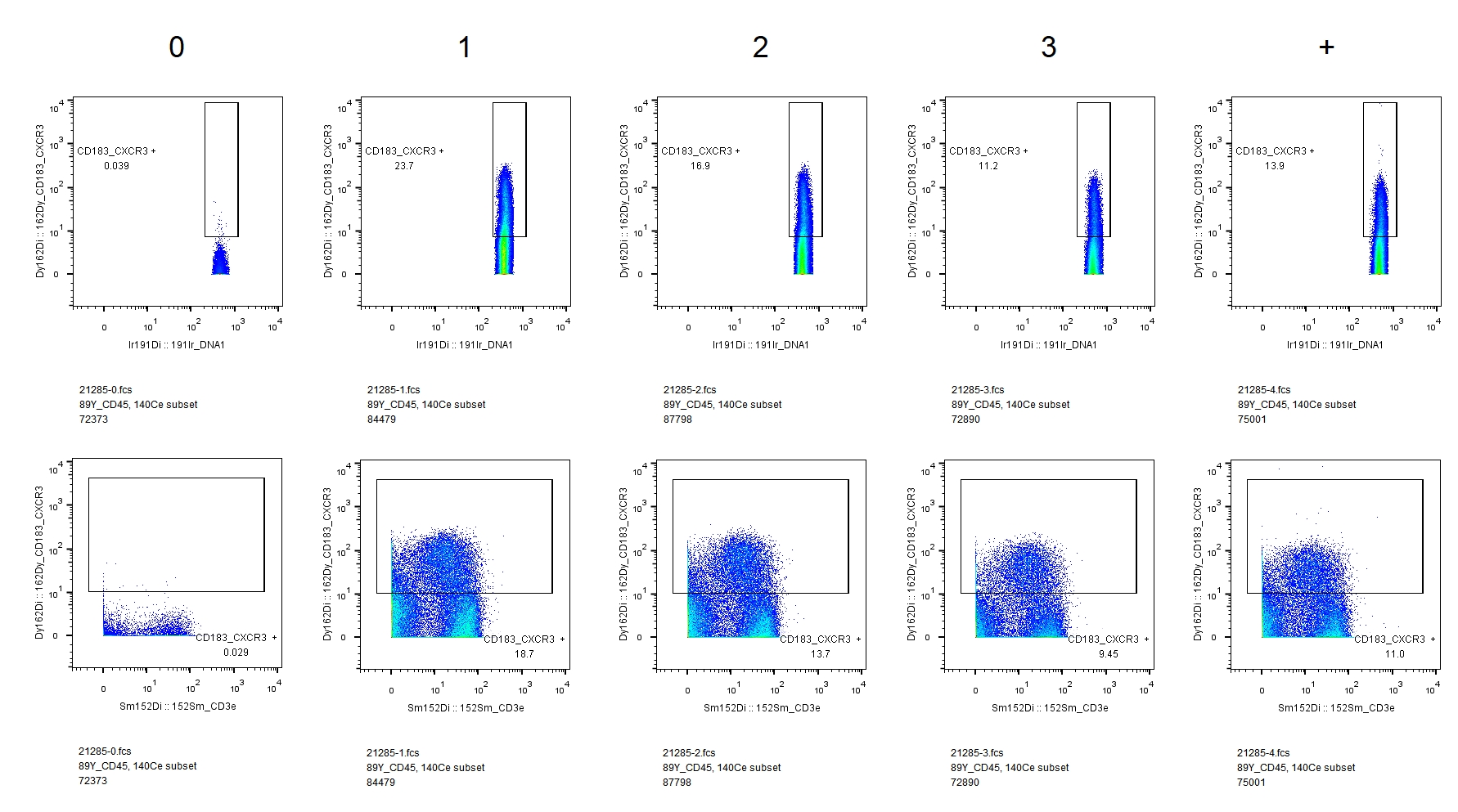

Supplement: Supplementary file 2 [file SupplementaryFile1.zip › 抗体测试结果图/21285-162-CD183CXCR3.jpg]

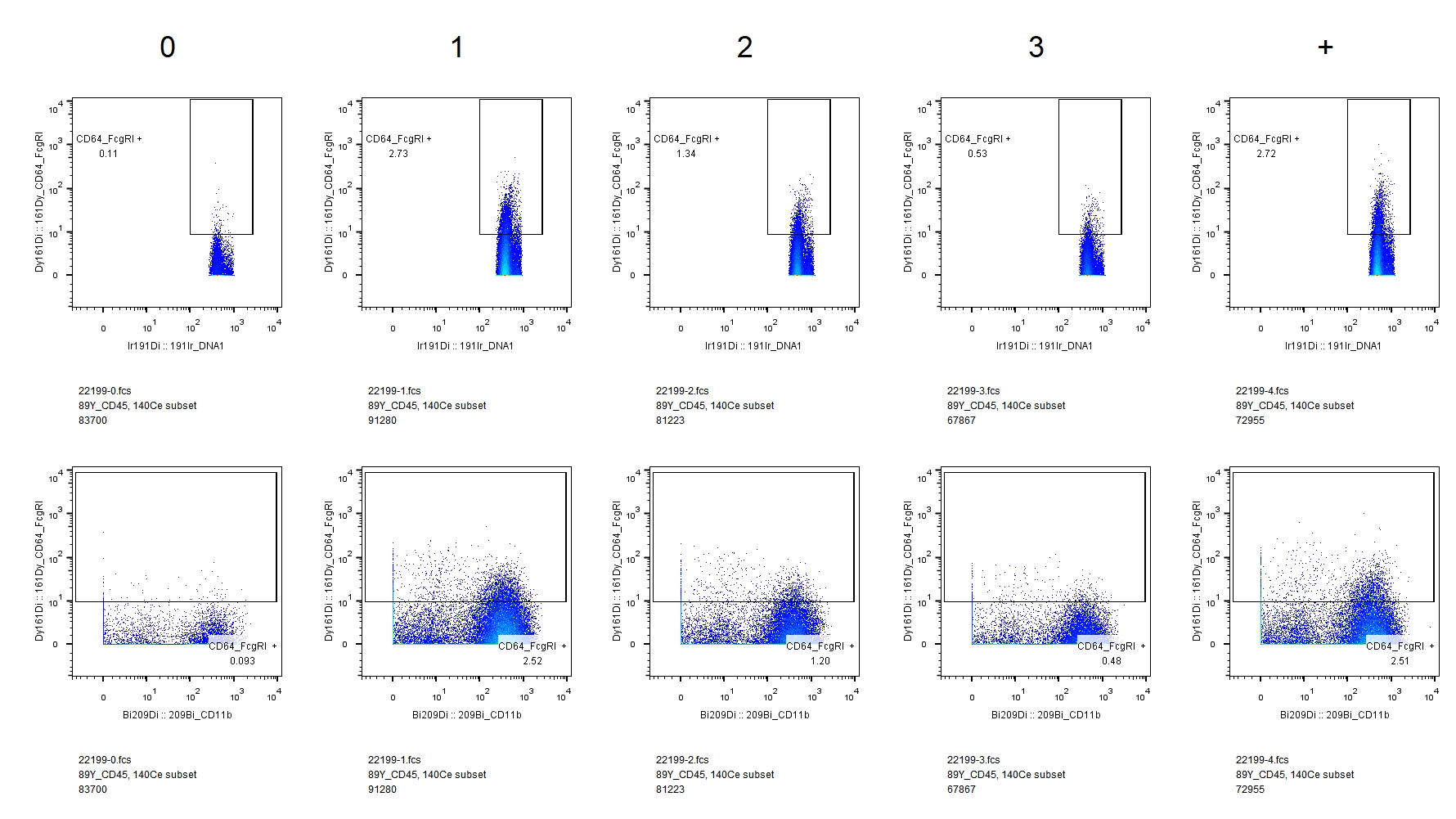

Supplement: Supplementary file 2 [file SupplementaryFile1.zip › 抗体测试结果图/22199-161-CD64-FcyRI.jpg]

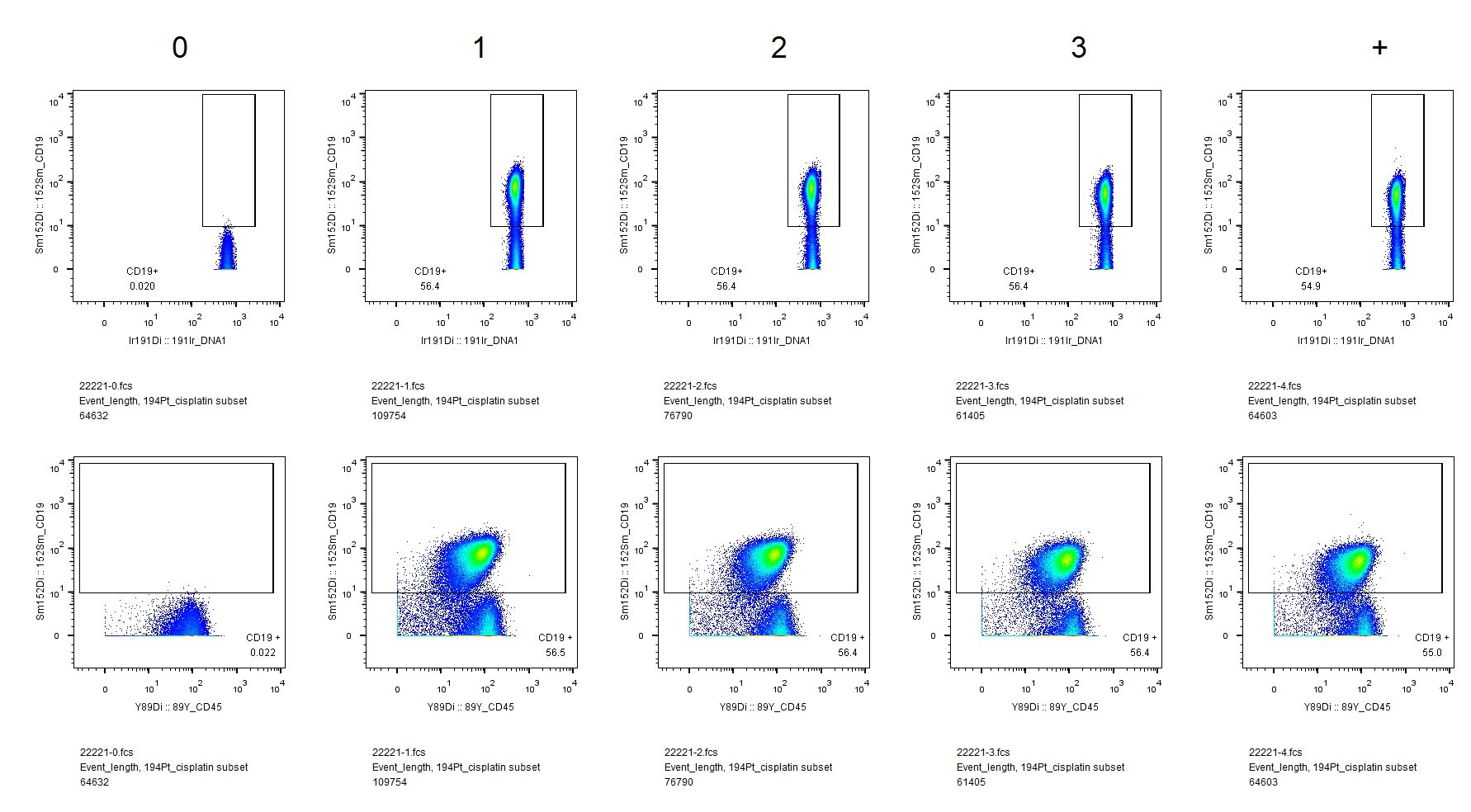

Supplement: Supplementary file 2 [file SupplementaryFile1.zip › 抗体测试结果图/22221-152-CD19.jpg]

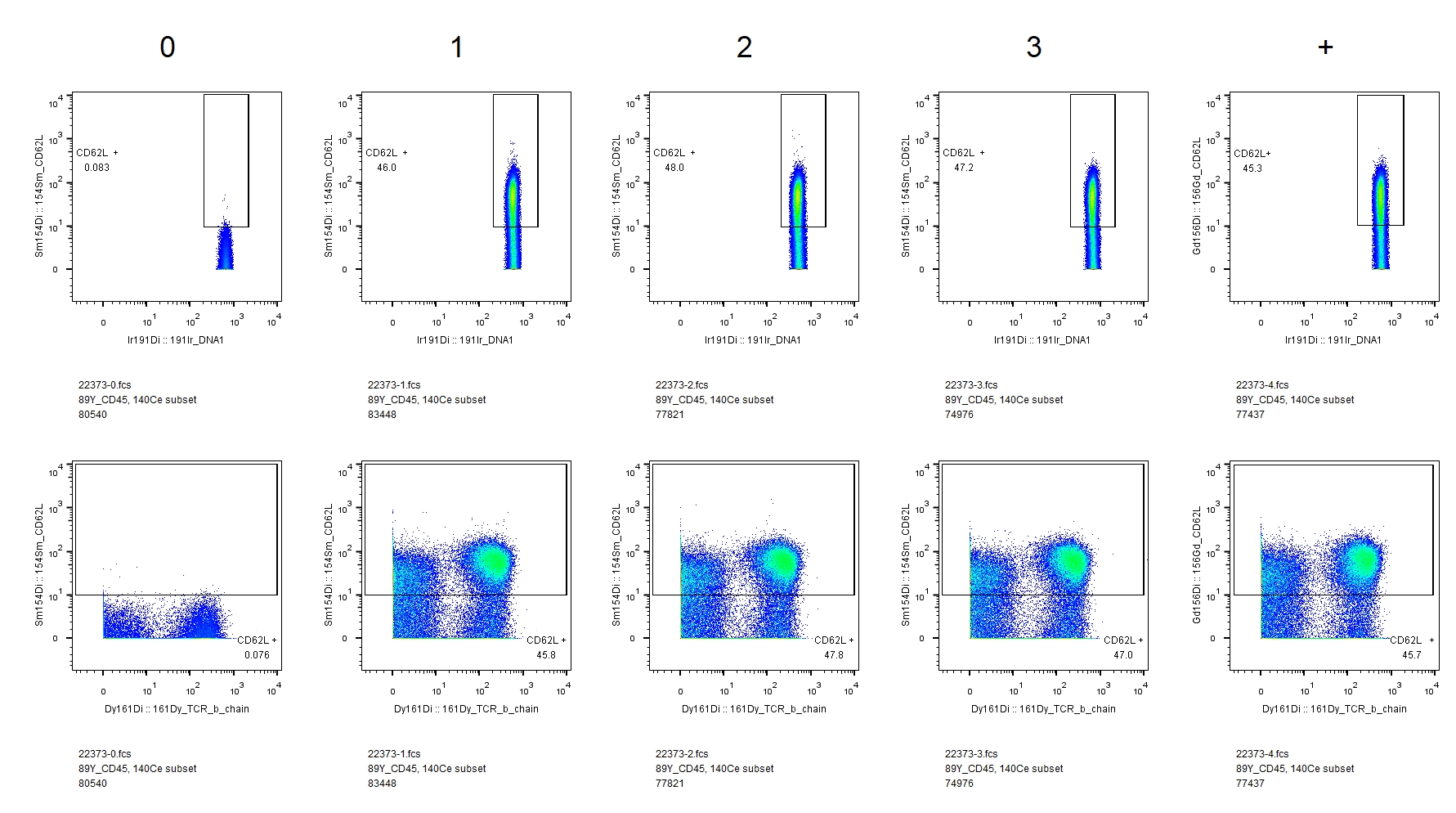

Supplement: Supplementary file 2 [file SupplementaryFile1.zip › 抗体测试结果图/22373-154-CD62L.jpg]

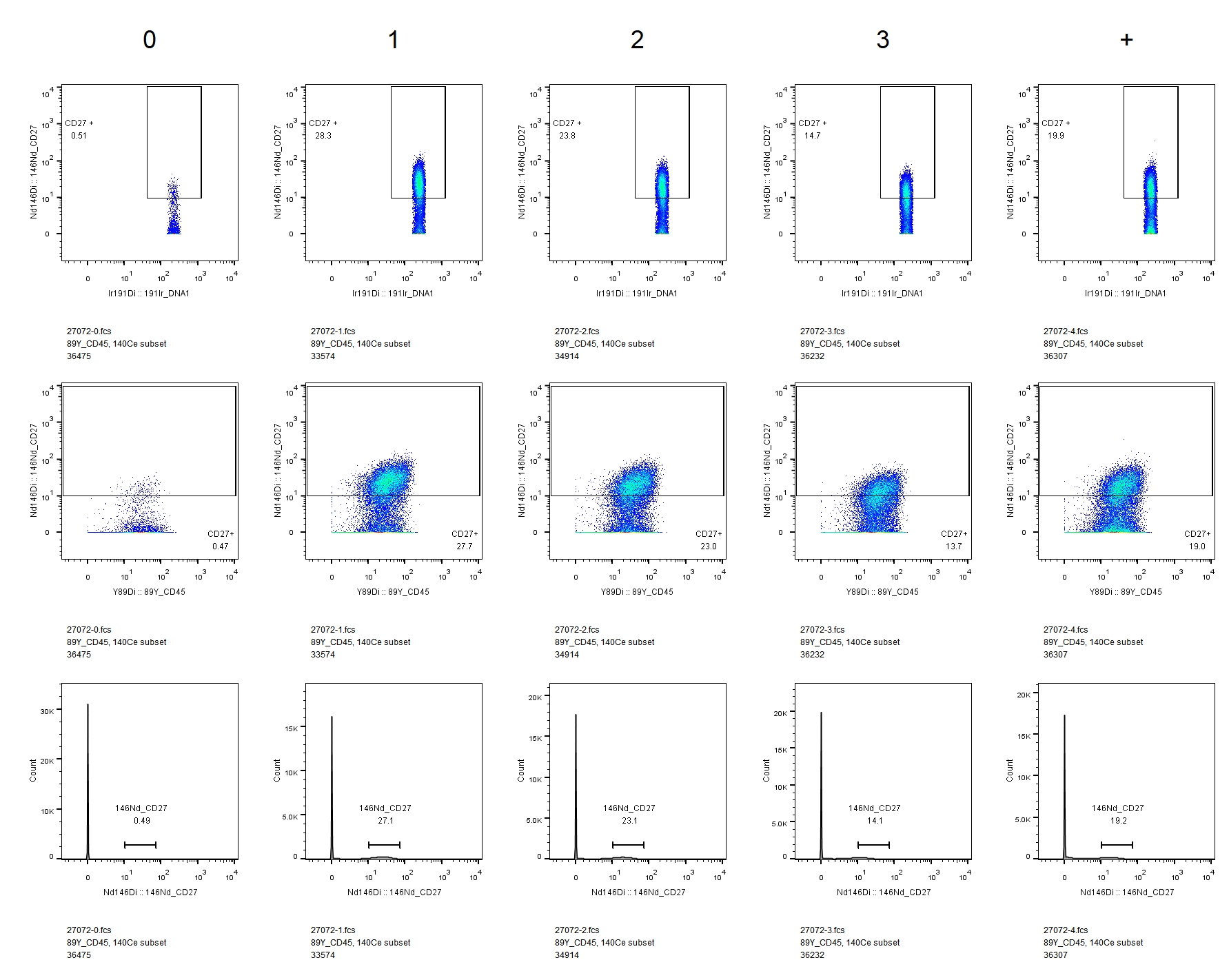

Supplement: Supplementary file 2 [file SupplementaryFile1.zip › 抗体测试结果图/27072-146-CD27.jpg]

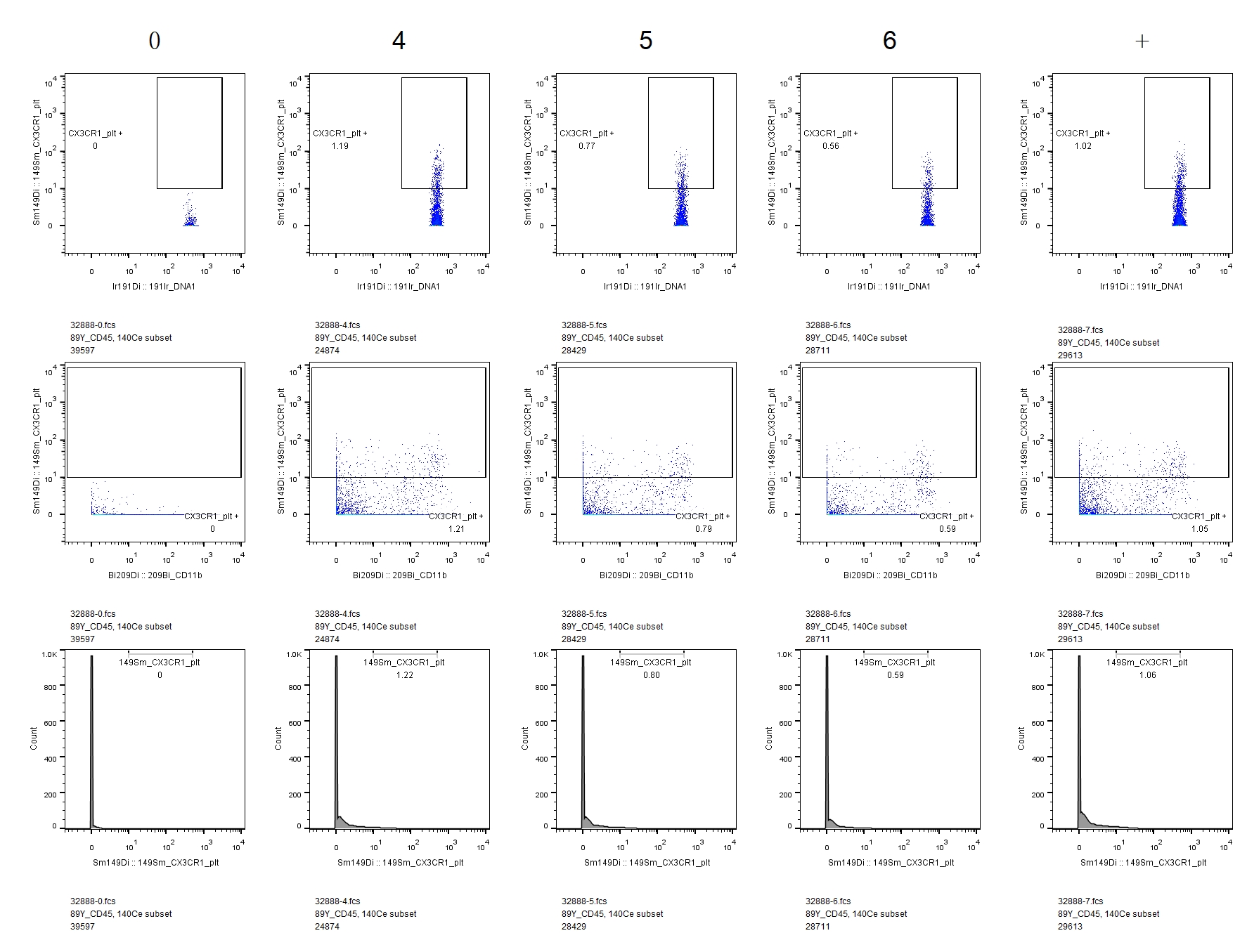

Supplement: Supplementary file 2 [file SupplementaryFile1.zip › 抗体测试结果图/32888-149-mCX3CR1_plt.jpg]

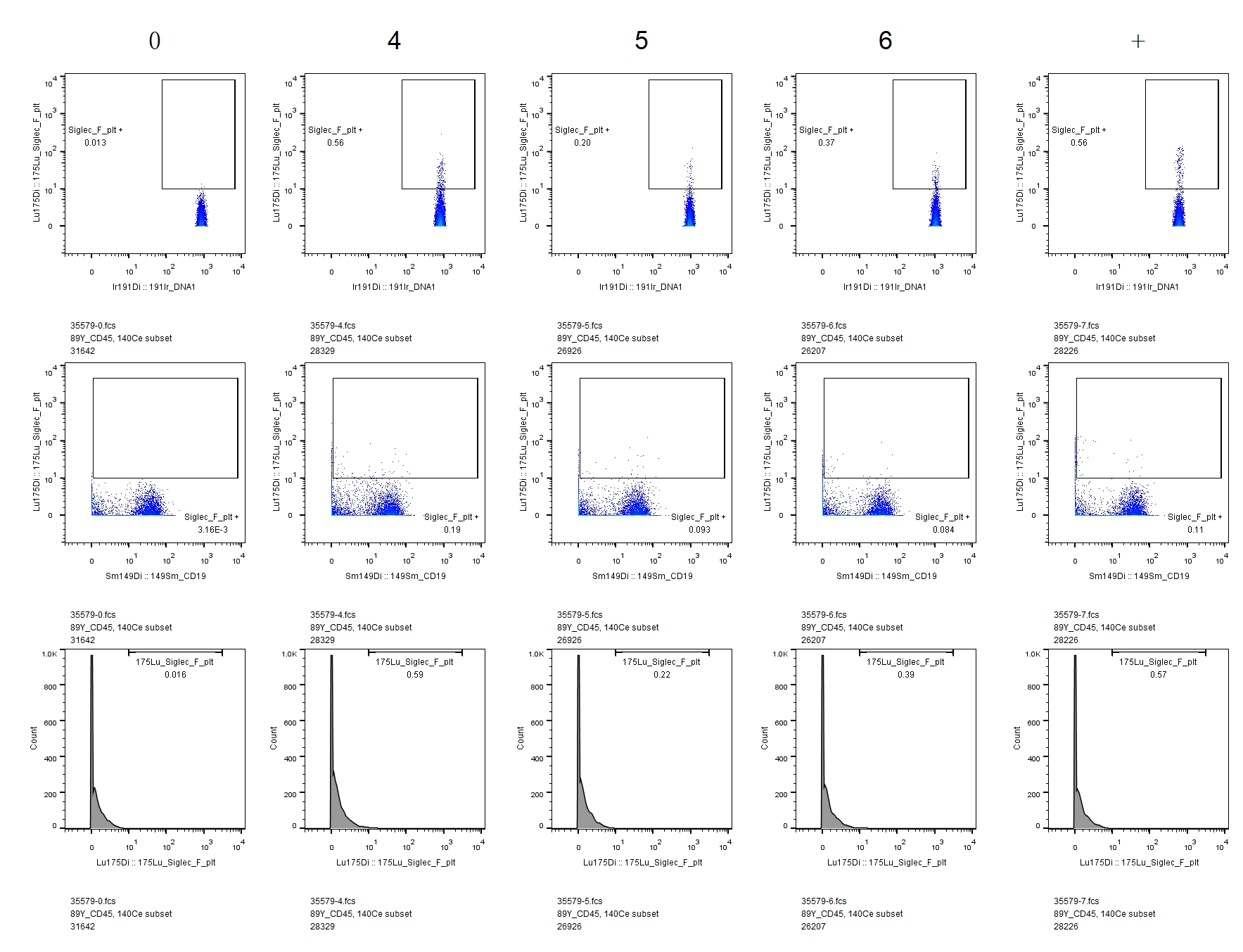

Supplement: Supplementary file 2 [file SupplementaryFile1.zip › 抗体测试结果图/35579-175-mSiglec-F_plt.jpg]

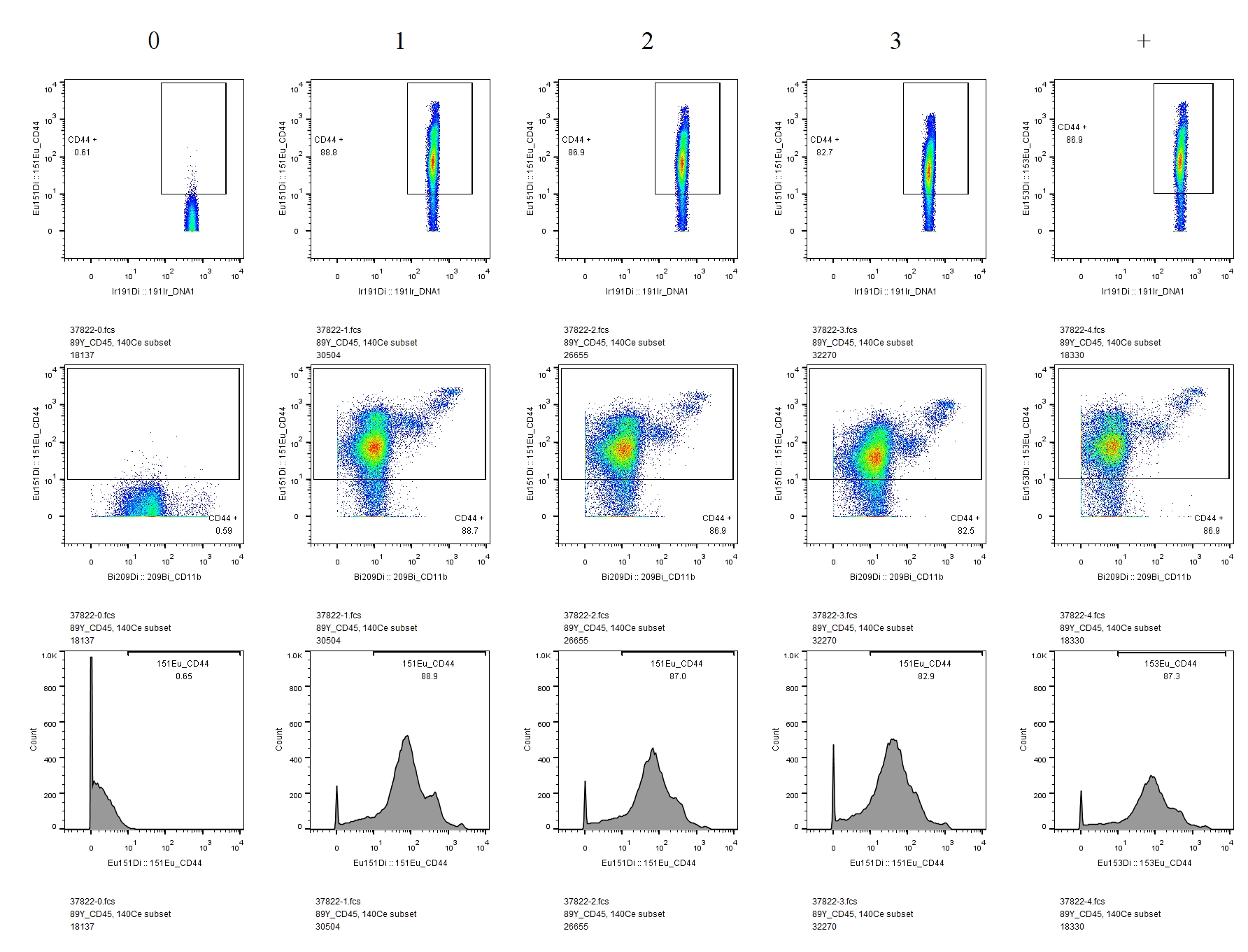

Supplement: Supplementary file 2 [file SupplementaryFile1.zip › 抗体测试结果图/37822-151-mCD44_plt.jpg]

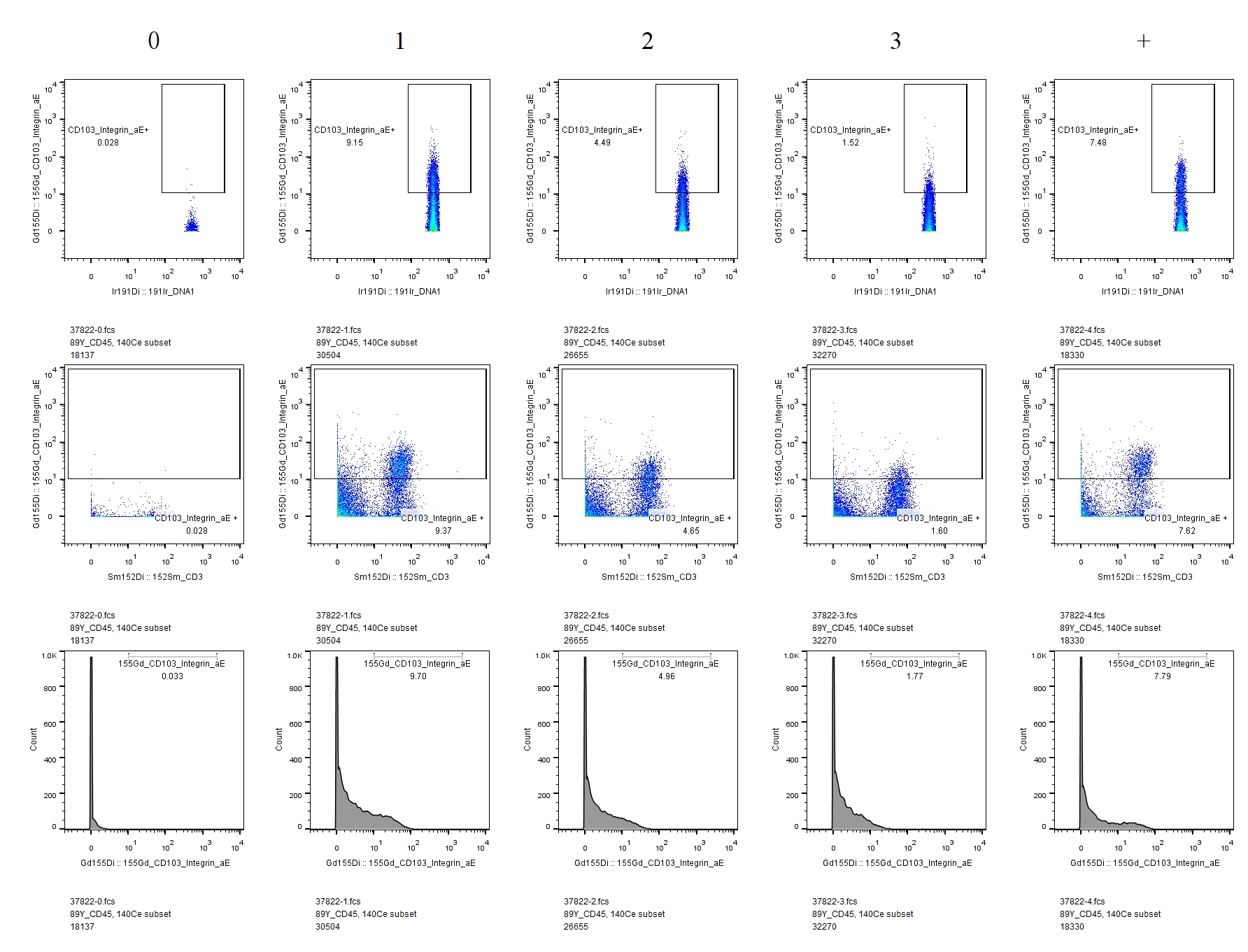

Supplement: Supplementary file 2 [file SupplementaryFile1.zip › 抗体测试结果图/37822-155-mCD103-Integrin-aE_plt.jpg]

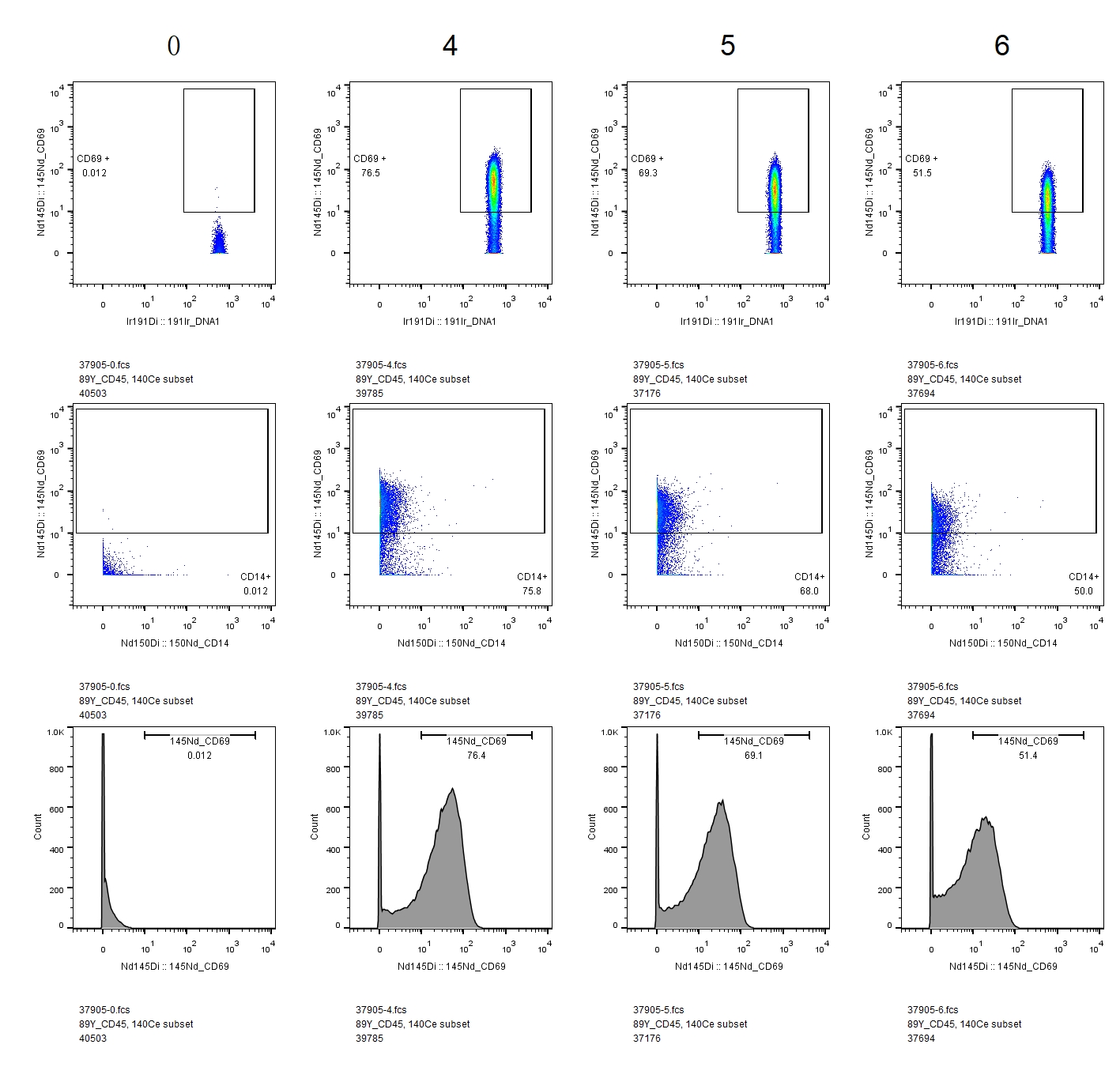

Supplement: Supplementary file 2 [file SupplementaryFile1.zip › 抗体测试结果图/37905-145-mCD69_plt.jpg]

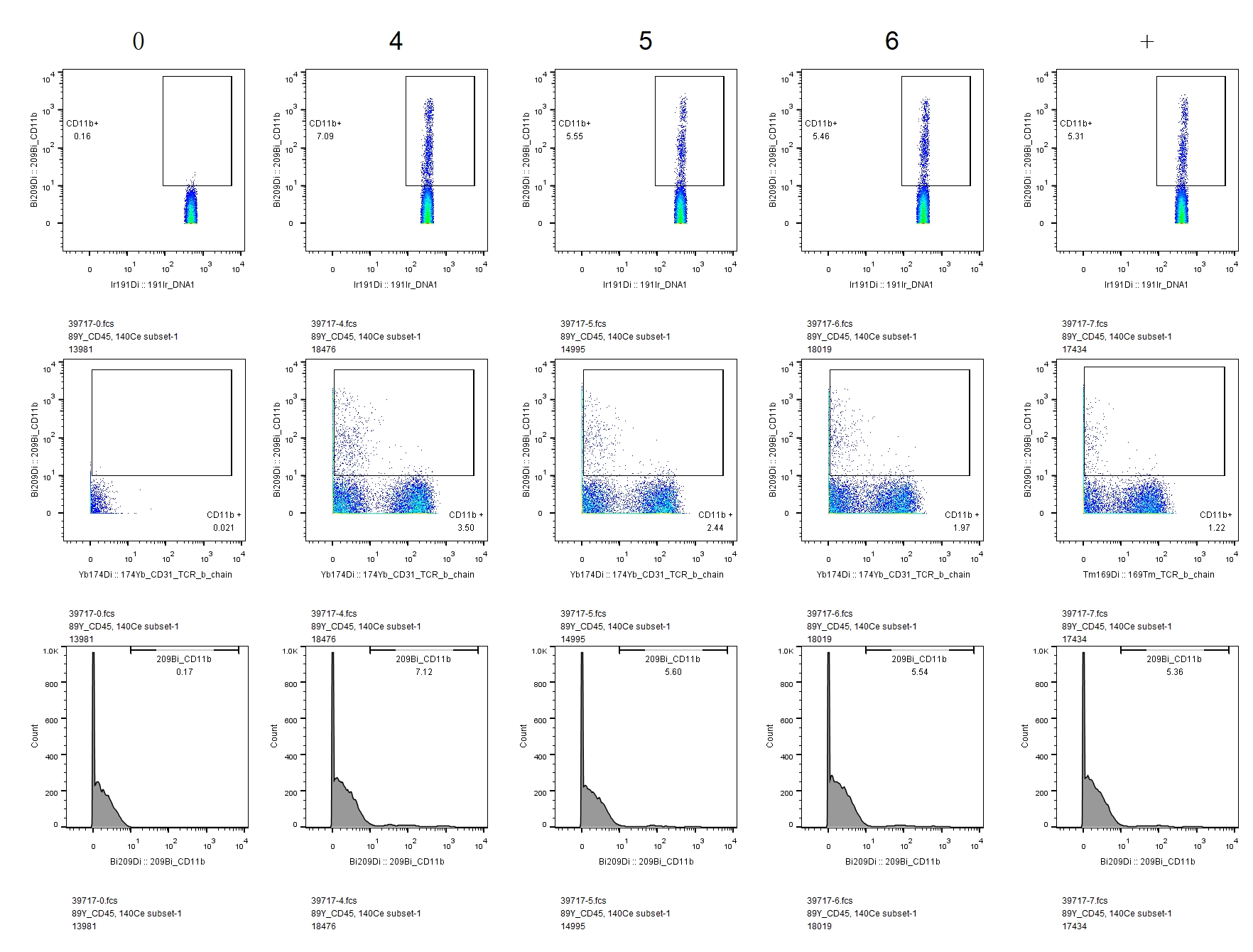

Supplement: Supplementary file 2 [file SupplementaryFile1.zip › 抗体测试结果图/39717-209-CD11b_plt.jpg]

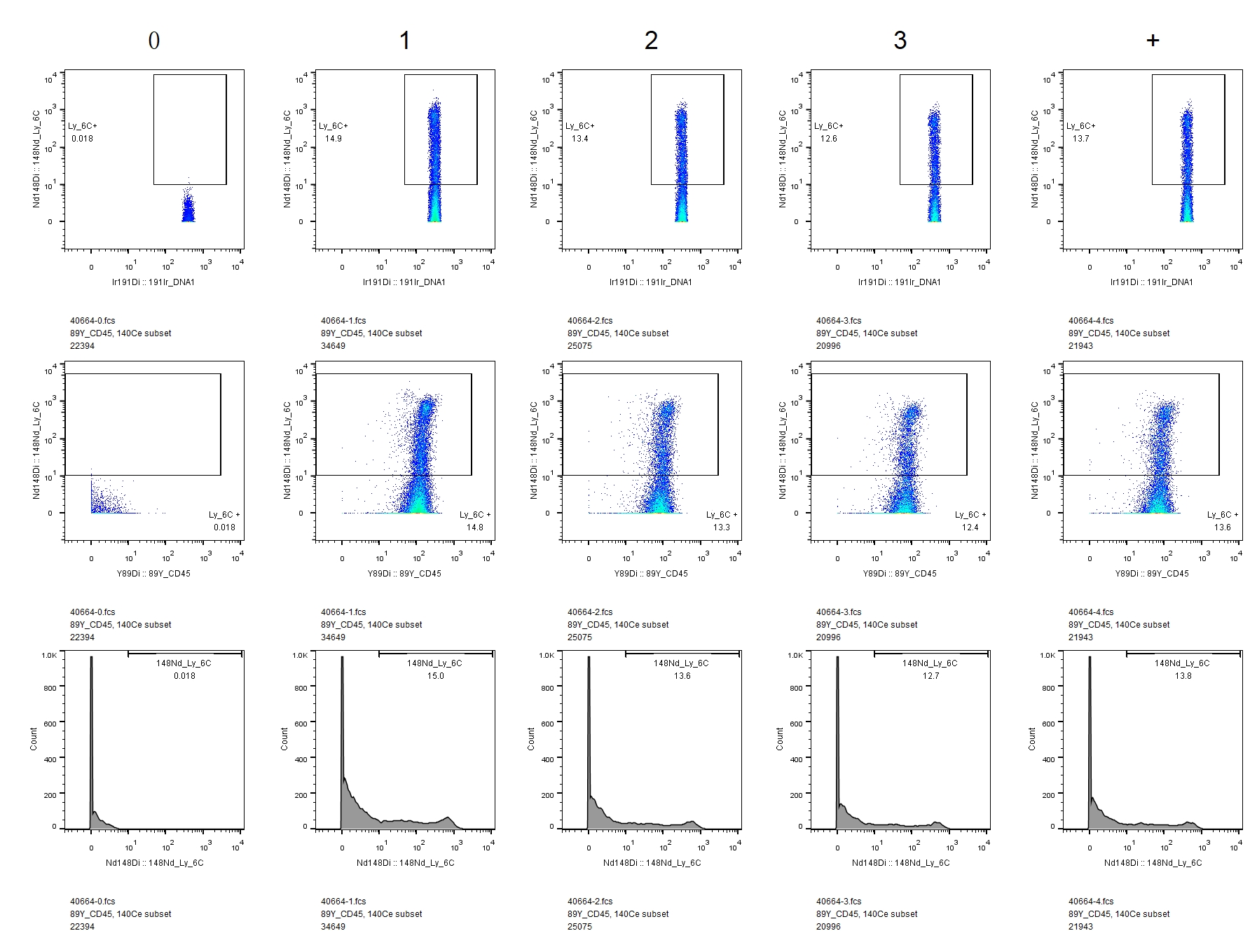

Supplement: Supplementary file 2 [file SupplementaryFile1.zip › 抗体测试结果图/40664-148-mLy-6C_plt.jpg]

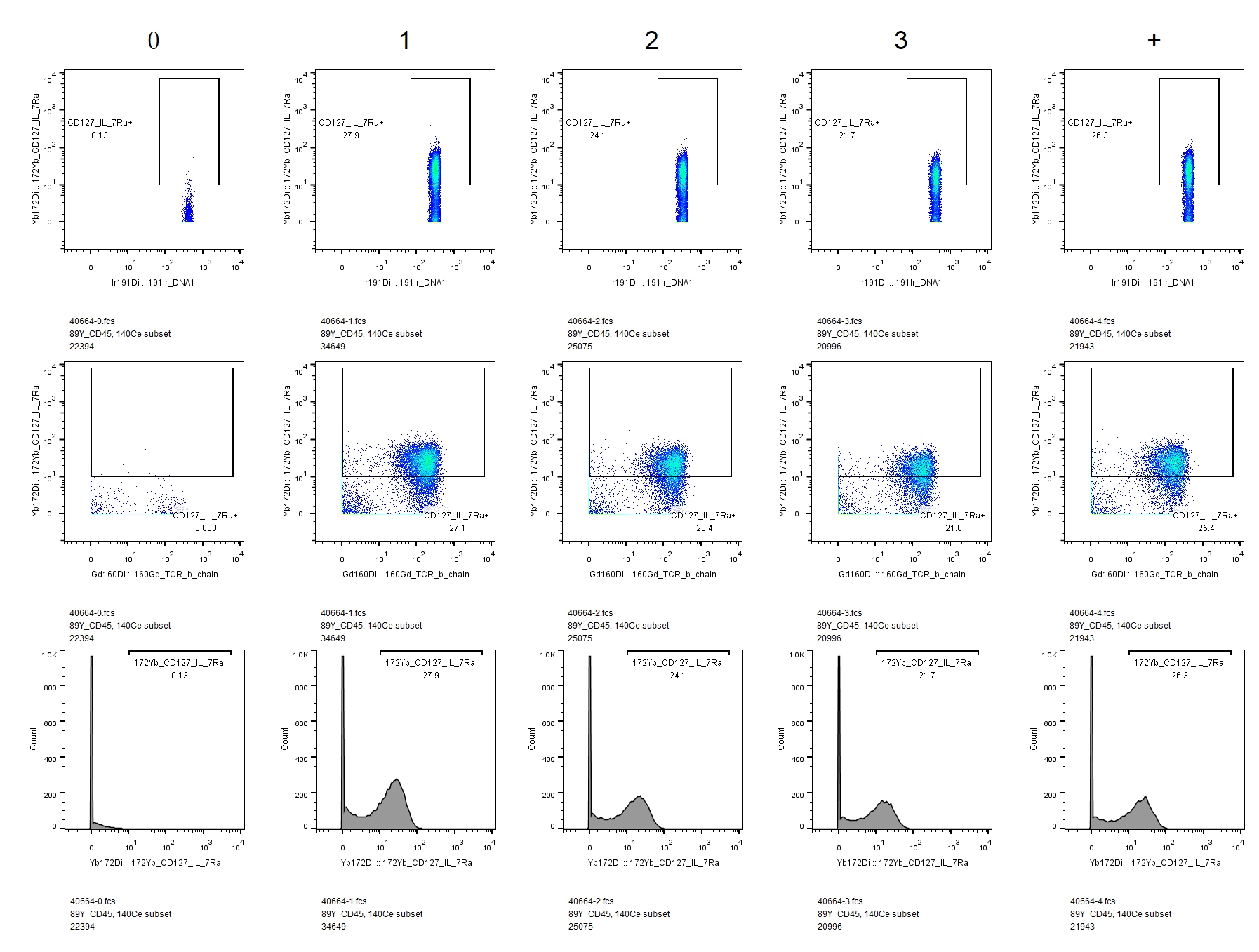

Supplement: Supplementary file 2 [file SupplementaryFile1.zip › 抗体测试结果图/40664-172-mCD127-IL-7Ra_plt.jpg]

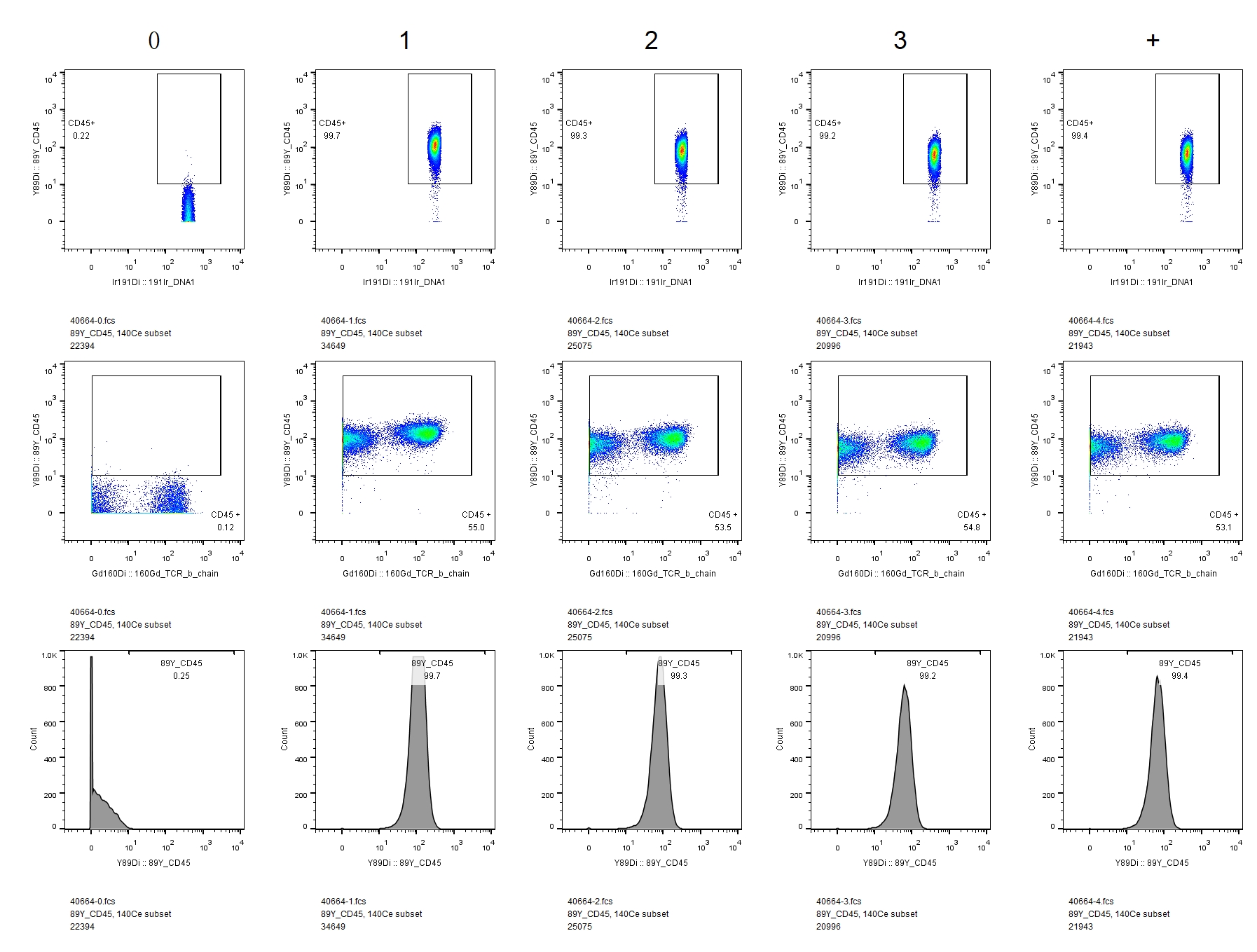

Supplement: Supplementary file 2 [file SupplementaryFile1.zip › 抗体测试结果图/40664-89-mCD45_plt.jpg]

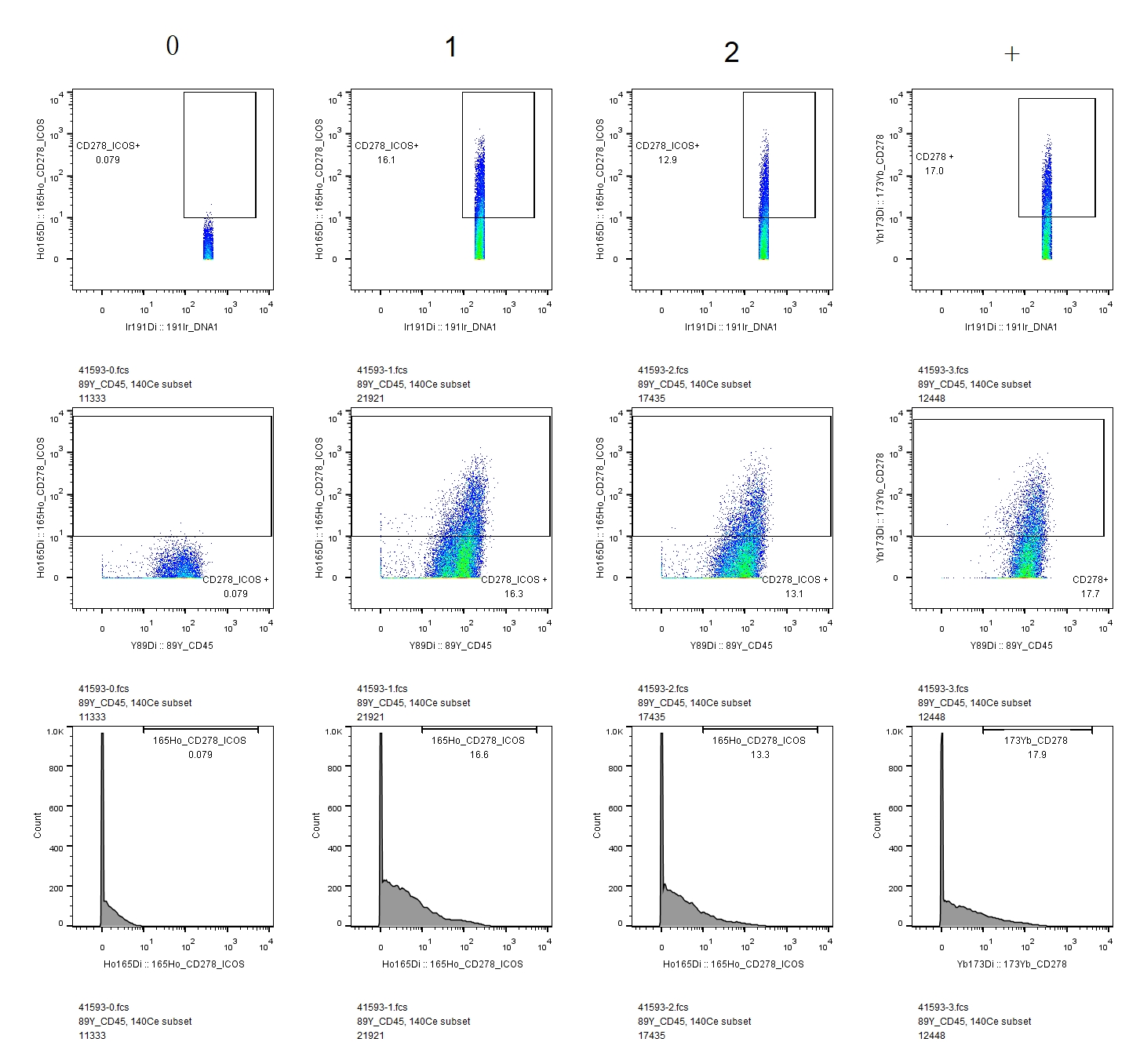

Supplement: Supplementary file 2 [file SupplementaryFile1.zip › 抗体测试结果图/41593-173-mCD278-ICOS.jpg]

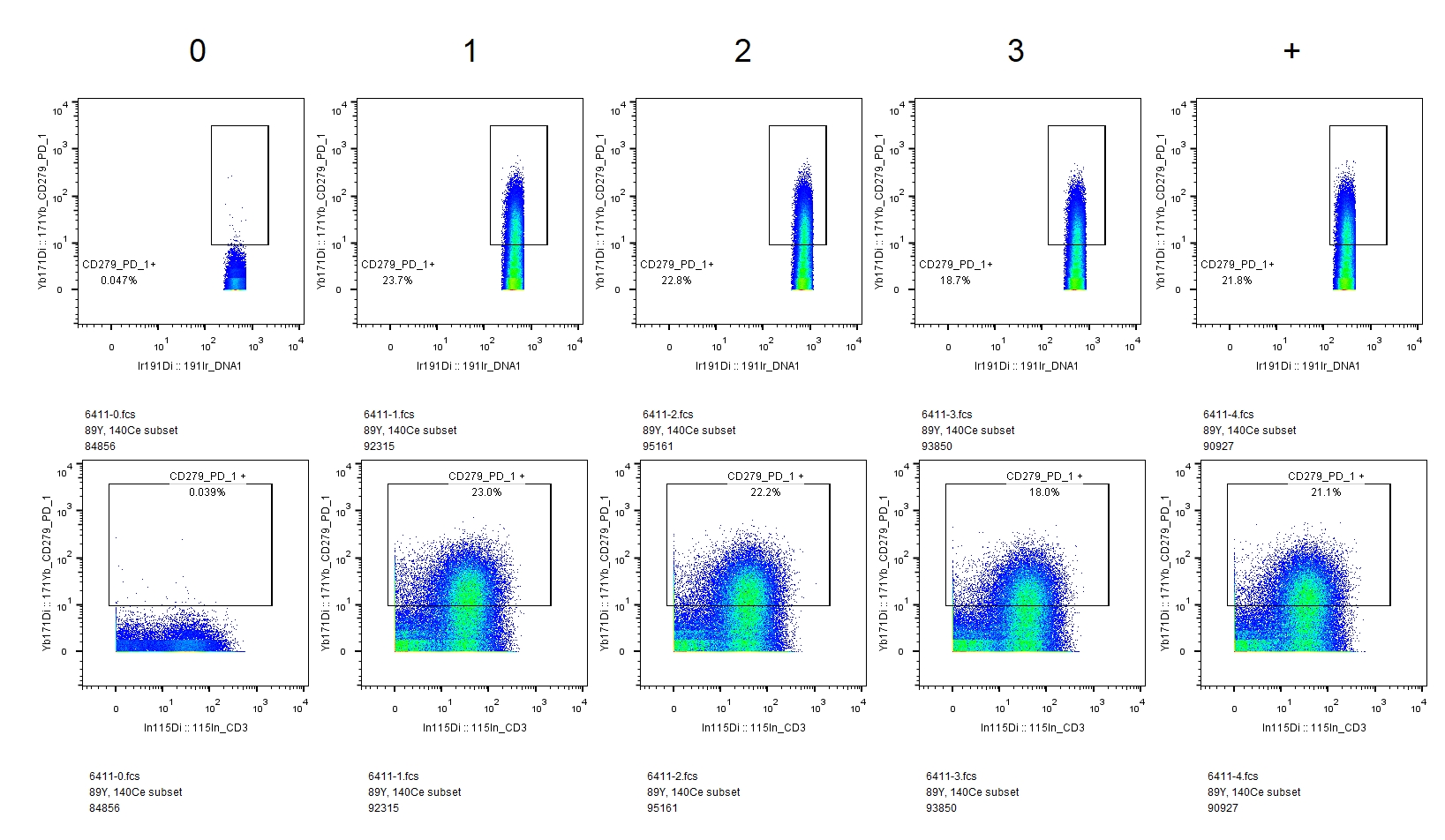

Supplement: Supplementary file 2 [file SupplementaryFile1.zip › 抗体测试结果图/6411-171-CD279-PD-1.jpg]

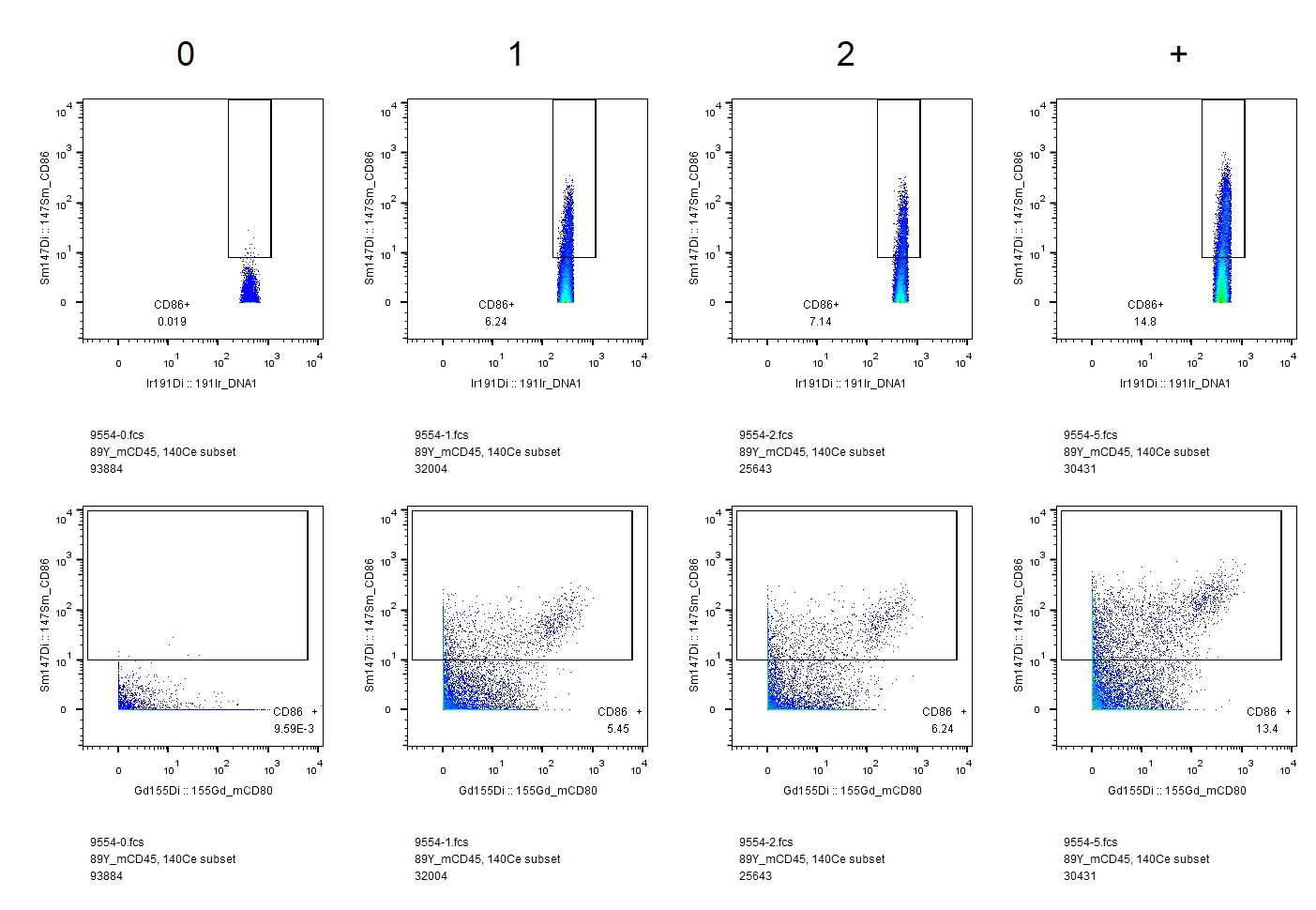

Supplement: Supplementary file 2 [file SupplementaryFile1.zip › 抗体测试结果图/9554-147-CD86.jpg]
